# Heterojunction‐Driven Stochasticity: Bi‐Heterojunction Noise‐Enhanced Negative Transconductance Transistor in Image Generation

**DOI:** 10.1002/adma.202505150

**Published:** 2025-06-25

**Authors:** Youngmin Han, Ryun‐Han Koo, Jaechan Song, Chang‐Hyun Kim, Eun Kwang Lee, Wonjun Shin, Hocheon Yoo

**Affiliations:** ^1^ Department of Electronic Engineering Hanyang University 222 Wangsimni‐ro Seoul 04763 Republic of Korea; ^2^ Inter‐University Semiconductor Research Center Seoul National University Seoul 08826 South Korea; ^3^ Department of Electrical and Computer Engineering Seoul National University Seoul 08826 South Korea; ^4^ Department of Artificial Intelligence Semiconductor Engineering Hanyang University 222 Wangsimni‐ro Seoul 04763 South Korea; ^5^ School of Electrical Engineering and Computer Science University of Ottawa Ottawa ON K1N 6N5 Canada; ^6^ Department of Chemical Engineering Pukyong National University Busan 48513 Republic of Korea; ^7^ Department of Semiconductor Convergence Engineering Sungkyunkwan University Suwon 16419 South Korea

**Keywords:** Bi‐heterojunction, low‐frequency noise, multi‐valued logic, negative transconductance, noise enhancement, stochastic electronics

## Abstract

Reliable true‐random number generator (TRNG) hardware demands amplified intrinsic noise and multi‐bit entropy output, which are difficult to achieve in conventional single‐device TRNG implementation. A bi‐heterojunction noise‐enhanced negative transconductance (BHN‐NTC) transistor is presented, incorporating an asymmetric PTCDI‐C13 layer into an NTC transistor. This design enhances electron injection, expanding the NTC region (19 → 27 V) and increasing negative transconductance (−0.036 µS at *V*_GS_ = −11 V → −0.073 µS at *V*_GS_ = −15 V) by reducing the electron injection barrier (≈2.13 eV → ≈0.41 eV). The bi‐heterojunction configuration introduces a strong correlation between noises, including trapping/detrapping and generation/recombination processes. This property enables a threefold higher entropy throughput in TRNG, achieving a 3‐bit output per sampling event. The BHN‐NTC‐driven TRNG leverages increased noise‐induced entropy to generate more diverse latent vectors, mitigating mode collapse and enabling the synthesis of high‐quality, realistic images. This significantly enhances StyleGAN2‐based image generation, improving performance metrics such as Frechet inception distance (FID) (18.7 → 8.3), kernel inception distance (KID) (0.024 → 0.009), inception score (IS) (6.5 → 9.2), and multi‐scale structural similarity (MS‐SSIM) (0.43 → 0.21). Consequently, the BHN‐NTC transistor establishes a scalable stochastic noise platform, advancing applications in secure electronics and probabilistic stochastic computing.

## Graphical abstract

This work engineered a bi‐heterojunction noise‐enhanced negative transconductance (BHN‐NTC) transistor using a half‐PTCDI‐C13 layer, achieving expanded and tunable noise characteristics. This advancement enables efficient multi‐bit TRNGs for AI‐driven image generation and enhances logic circuit applications.

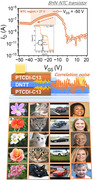

## 1 Introduction

Transistors are the fundamental building blocks of modern electronic devices and systems, and they have contributed essential switching elements in integrated circuits. The core functionality of a transistor is determined by its behavior to switch between conductive and insulating states depending on the applied gate voltage. In conventional transistors, the switching behavior is well‐defined: *p*‐type transistors turn on at negative gate voltages,^[^ ^1^ ^]^ while *n*‐type transistors turn on at positive gate voltages.^[^ ^2^ ^]^ This complementary operation forms the basis of complementary metal‐oxide‐semiconductor (CMOS) technology, which enables efficient digital logic, low power consumption, and stable circuit performance.^[^ ^3^ ^]^ The ability to selectively control charge carriers in this manner has long been the defining characteristic of transistor functionality, providing the foundation for nearly all electronic systems.

While traditional transistors exhibit a monotonic increase in source‐drain conductance with gate voltage, recent advances have challenged this paradigm by introducing non‐monotonic switching behaviors that enable new functional modalities. As a representative example, *pn*‐anti‐ambipolar transistors,^[^ ^4^ ^]^ which integrate *p*‐type and *n*‐type layers in series, have attracted significant attention. This architecture produces a peak‐and‐dip conductance profile, which has been harnessed for multi‐valued logic (MVL),^[^ ^5^ ^]^ neuromorphic computing,^[^ ^6^ ^]^ frequency multiplication,^[^ ^7^ ^]^ and optoelectronics.^[^ ^8^ ^]^ Building on this foundation, researchers have further explored *N*‐shaped transistors, where an additional parallel *p*‐type channel expands the operational landscape to include four distinct states: (i) off‐state, (ii) transistor‐on, (iii) negative transconductance (NTC) region, and (iv) a well‐defined on‐state, having a peak‐and‐valley conductance profile.^[^ ^9^ ^]^ This extended switching framework has been shown to enhance noise margins in ternary circuits^[^ ^10^ ^]^ and implement spiking neurons,^[^ ^11^ ^]^ underscoring its potential for next‐generation information processing.

NTC‐transistors operate through the simultaneous injection and transport of both holes and electrons, enabling a nonlinear conduction profile that departs from conventional transistor behavior. The degree to which these charge carriers are injected and interact fundamentally dictates the emergence of NTC behaviors, providing a tunable framework for optimizing device performance. At heterojunction interfaces where both hole and electron transport coexist, entirely new physical phenomena can emerge—phenomena that remain inaccessible in conventional single‐channel transistors. These junctions offer a platform for investigating charge dynamics that are not observed in conventional transistors, particularly in relation to noise characteristics influenced by carrier trapping,^[^ ^12^ ^]^ recombination,^[^ ^13^ ^]^ and interfacial fluctuations.^[^ ^14^ ^]^ This suggests that, beyond altering transport properties, NTC transistors could be utilized as controllable noise sources, offering potential applications in stochastic electronics and randomness‐based systems.

Parallel to NTC research, the past decade has witnessed rapid progress in hardware true‐random‐number generators (TRNGs) that harvest intrinsic device noise for cryptography and stochastic computing. Representative examples include diffusive Ag:SiO₂ memristors whose stochastic filament‐formation delay yields bias‐free entropy,^[^ ^15^ ^]^ h‐BN‐based RTN memristors,^[^ ^16^ ^]^ and floating‐body “cryptoristor” FinFETs that convert avalanche noise into random bits with low power consumption.^[^ ^17^ ^]^ Although these approaches demonstrate excellent cryptographic quality, they typically require external analog gain stages or output only one random bit per sampling event, thereby limiting entropy density and complicating circuit integration. Stochastic‐computing hardware also leverages device noise to accelerate probabilistic inference. However, existing implementations have predominantly relied on conventional CMOS ring‐oscillator RNGs or software‐derived seeds, leaving a gap between emerging device‐level entropy sources and application‐level AI workloads. Bridging this gap demands a noise generation platform that (i) amplifies entropy inside the device to avoid peripheral overhead and (ii) delivers multi‐bit output to match the bandwidth requirements of modern generative models.

**Figure 1:**
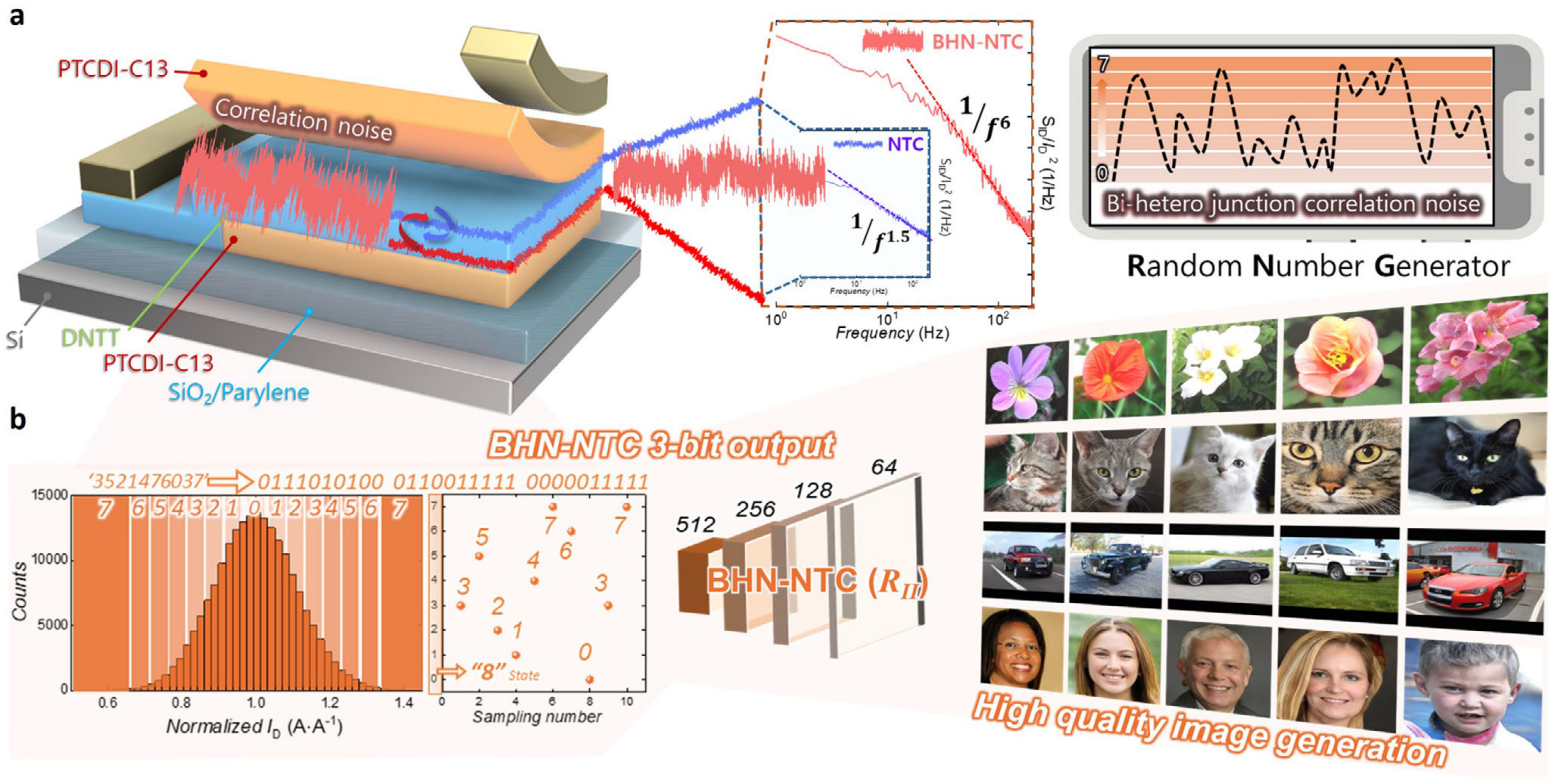
a) Comprehensive schematic diagram of research that utilizes the high‐level of noise from two heterojunctions in a BHN‐NTC transistor structure to implement a hardware‐based reliable TRNG. b) Based on the high noise fluctuations of the BHN‐NTC transistor extracted from *Region II*, the normalized *I*_D_ data was converted into 3‐bit outputs ranging from 0–7. These outputs were then used as an input source for the image generator, producing high‐resolution and accurate images of flowers, cats, cars, and people.

Reflecting the structural exploration of the previously described heterojunction interfaces, in this work, we introduce a bi‑heterojunction noise‑enhanced negative‑transconductance (BHN‑NTC) transistor, which extends the conventional NTC architecture by integrating an asymmetric PTCDI‑C13 layer. The dual heterojunction simultaneously widens the electron–hole overlap region and intrinsically amplifies noise through correlated carrier trapping and recombination, thereby supplying three statistically independent bits per sampling event without an external amplifier—an entropy density not yet reported for single‑device TRNGs. This structural modification enables enhanced electron injection, effectively widening the NTC region (*V*_valley_ – *V*_peak_) and increasing peak current levels. The bi‐heterojunction interfaces provide a new degree of control over hole and electron charge transport, leading to distinct noise characteristics that were not present in single‐junction NTC transistors (**Figure** **1**a). By means of this significantly amplified noise randomness, the BHN‐NTC transistor establishes a reliable hardware‐based TRNG. Furthermore, the high‐entropy noise source from the BHN‐NTC transistor enhances image generation quality in a StyleGAN2 framework, improving key performance metrics such as Frechet inception distance (FID) from 18.7 to 8.3 and inception score (IS) from 6.5 to 9.2. This richer stochastic response delivers a far more diverse latent vector than that of a single‐junction NTC device; consequently, StyleGAN2 produces natural, detailed images when seeded with BHN‐NTC noise. The enhanced stochastic response, driven by correlated carrier fluctuations at the heterojunctions, offers a scalable approach to high‐quality randomness, making the BHN‐NTC transistor a promising candidate for stochastic electronics (Figure 1b).

## 2 Results and Discussion

**Figure 2:**
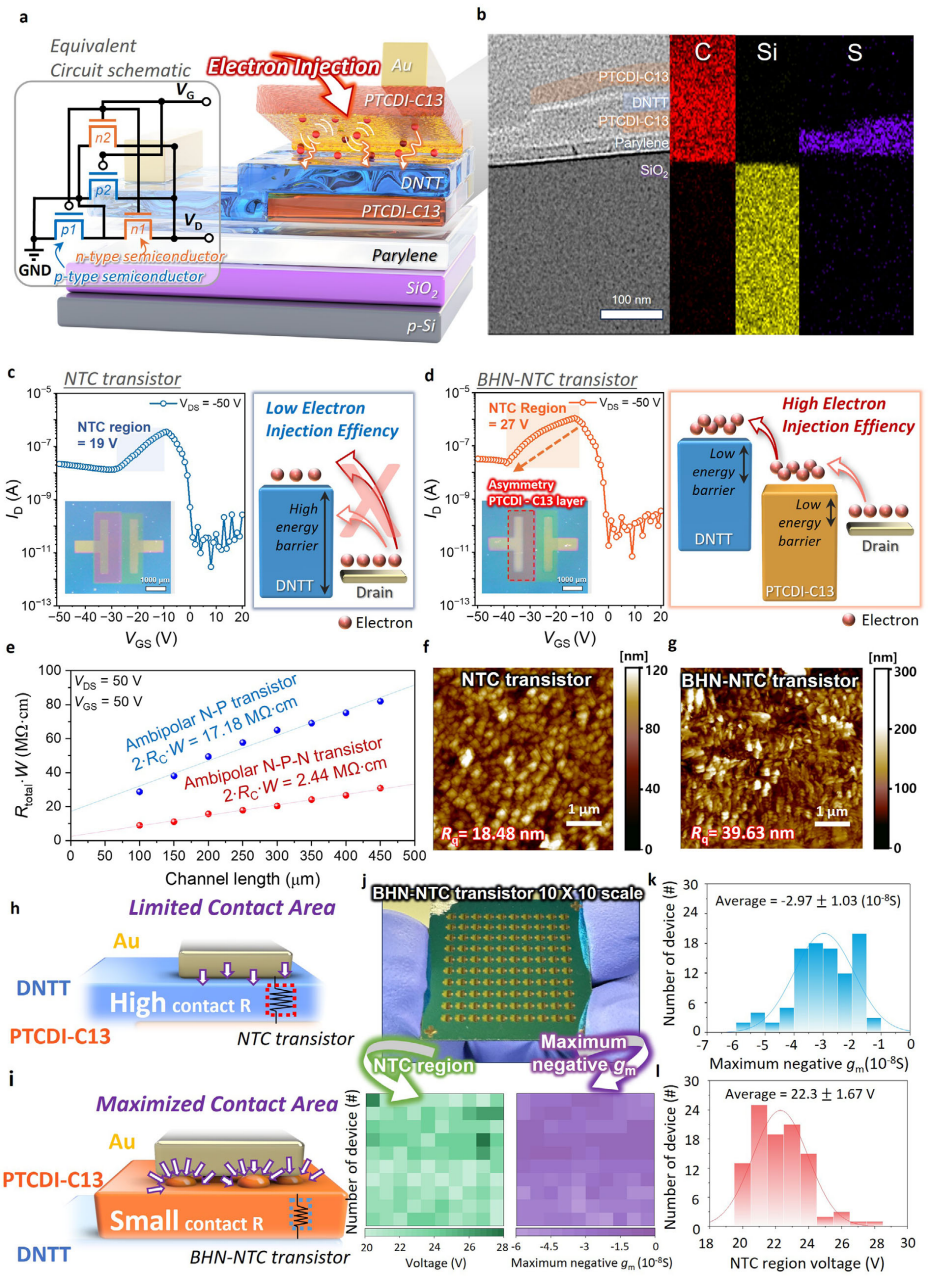
a) Schematic diagram of BHN‐NTC transistor with an equivalent circuit to explain the additional heterojunctions and electron injections. b) Cross‐section TEM and EDS element mapping image of the BHN‐NTC transistor to identify the sequential stacking of proposed structures. c) Transfer curve of NTC transistor with NTC region and schematic diagram of electron injections drain electrode to DNTT channels. The inset figure in the transfer curve displays VTC characteristics of complementary‐like ternary inverter circuits, where the NTC transistor functions as a PMOS and PTCDI‐C13 serves as an NMOS. The middle logic region exhibits 17 V∙V^−1^. d) Transfer curve of BHN‐NTC transistor with NTC region and schematic diagram of electron injections drain electrode to PTCDI‐C13/DNTT channels. The inset figure in the transfer curve displays VTC characteristics of complementary‐like ternary inverter circuits, where the BHN‐NTC transistor functions as a PMOS and PTCDI‐C13 serves as an NMOS. The middle logic region exhibits 27.5 V∙V^−1^. e) Contact resistance when electrons are injected from the drain electrode into DNTT (NTC transistor) and PTCDI‐C13 (BHN‐NTC transistor) in ambipolar transistor structures. These ambipolar transistors were fabricated with the same stacking order and materials as the NTC and BHN‐NTC transistors to identify contact resistance effects. AFM images of f) NTC transistor and g) BHN‐NTC transistor, showing the film morphology. R_q_ values were 18.48 and 39.63 nm, respectively. Schematic diagram of the contact area between h) Au‐DNTT (NTC‐transistor) i) Au‐PTCDI‐C13 (BHN‐NTC transistor). j) OM images of 10 **×** 10 scale BHN‐NTC transistors with NTC region, maximum negative *g*_m_ mapping data to investigate the device uniformity and reliability. Histogram data for statistical analysis of the electrical parameters k) maximum negative *g*_m_, l) NTC region of BHN‐NTC transistor on a 10 **×** 10 scale.

First, we fabricated the BHN‐NTC transistor by integrating an asymmetric PTCDI‐C13 layer, an *n*‐type semiconductor, a top the conventional NTC structure (**Figure** **2**a). Asymmetric PTCDI‐C13 layer refers to a structure in which PTCDI‐C13 is selectively deposited only beneath the drain electrode, rather than uniformly across the entire channel. This design enhances carrier injection and expands the noise spectrum for functional improvements. In conventional *p*‐type NTC transistors, hole injections from the source and electron injection from the drain yield distinct transfer characteristics. In this structure, due to the *p*‐type nature of both source‐ and drain‐side semiconductors, electron injection from the drain is inefficient. The BHN‐NTC transistor circumvents this limitation by incorporating a parallel PTCDI‐C13 half‐asymmetric layer, converting the drain‐side semiconductor to *n*‐type and significantly improving electron injection. Transmission electron microscopy (TEM) analysis (Figure 2b) confirms the structural integrity of the BHN‐NTC transistor, with TEM imaging and elemental mapping clearly distinguishing the SiO₂ layer and verifying the formation of both the NTC structure and the PTCDI‐C13 layer.

Prior to investigating the operating mechanism and characteristics of the NTC and BHN‐NTC transistors, it was considered beneficial to first investigate the characteristics of the anti‐ambipolar transistor (AAT), which features a series connection of *p*‐type and *n*‐type semiconductors, resembling the basic configuration of the NTC structure. Thus, AAT operating characteristics and underlying mechanism were analyzed using an equivalent circuit, and a detailed discussion is provided in Supporting Information (Figure S1 and Note S1, Supporting Information). To examine the enhanced electron injection in the BHN‐NTC transistor, we measured the electrical characteristics of both the conventional NTC transistor and the proposed BHN‐NTC transistor (Figure 2c,d). Based on the operating characteristics, the distinct operation regions of the NTC and BHN‐NTC transistors were defined and correlated with their respective equivalent circuit models. A detailed description of each operation region is provided in the Supporting Information (Figure S2, Supporting Information). *Region I* is the range where the current flowing through N_1_ and P_1_ reaches its maximum. In this region, N_1_ and P_1_, which are connected in series, are both turned on, achieving peak conductivity and resulting in the highest current. *Region II* is defined as the NTC region, where the current through P_1_ continues to increase, but the series‐connected N_1_ begins to turn off, causing an overall decrease in current. This results in a phenomenon where the current decreases despite an increasing gate voltage. *Region III* refers to the area beyond the valley point. In this region, N_1_ continues to turn off, while P_2_ turns on, making the current through P_2_ dominant. Based on these regions, we compared the characteristics of the NTC and BHN‐NTC transistors. The NTC transistor exhibited an NTC region of 19 V, negative transconductance (*g*_m_) of −0.036 µS at *V*_GS_ = −11 V, and a peak current of 0.352 µA at *V*_GS_ = −9 V (Figure S3a, Supporting Information). In contrast, the BHN‐NTC transistor, with the addition of the asymmetric half PTCDI‐C13 layer, showed an NTC region of 27 V, *g*_m_ of −0.073 µS at *V*_GS_ = −15 V and a peak current of 1.109 µA at *V*_GS_ = −13 V (Figure S3b, Supporting Information). This indicates a higher peak current and an extended NTC region compared to the NTC transistor. To analyze the underlying reason for this behavior, we illustrated a simplified mechanism schematic based on an energy‐band diagram (Figure 2c,d; Figures S4 and S5, Supporting Information). Figure S4 (Supporting information) is a band diagram designed to verify the electron injection mechanism of the BHN‐NTC transistor, while Figure S5 (Supporting information) presents the UV‐visible spectroscopy (UV–vis) measurement results used to determine the bandgap. The UV photoelectron spectroscopy (UPS) data of DNTT, PTCDI‐C13, and Au used in the flat‐band diagram schematic are based on previous research results.^[^ ^18^ ^]^

A conventional *p*‐type NTC transistor consists of a half *n*‐type semiconductor and a fully deposited *p*‐type semiconductor, where hole carriers are injected from the source and electron carriers are injected from the drain. In this case, electrons must move from the drain into the lowest unoccupied molecular orbital (LUMO) of the *p*‐type semiconductor. However, this process requires overcoming a high energy barrier (≈2.13 eV), making electron injections highly challenging (Figure 2c). Therefore, we designed the structure to facilitate much easier electron transport by introducing a PTCDI‐C13 layer with a lower LUMO between the drain and DNTT, thereby reducing the energy barrier difference.^[^ ^19^ ^]^ In the BHN‐NTC transistor, the addition of the PTCDI‐C13 layer (N_2_) significantly reduces the energy barrier to ≈0.41 eV, allowing for more efficient electron injections into the channel. To investigate the charge injection barrier characteristics under actual applied voltages and to confirm the charge injection efficiency enhancement mechanism of the BHN‐NTC transistor, we measured the electrical characteristics of both NTC and BHN‐NTC transistors under varying temperatures and performed Arrhenius plot analysis. As a result, a smaller charge injection barrier was observed in the BHN‐NTC transistor, and further details are provided in the Supporting Information (Figure S6 and Note S2, Supporting Information). These results indicate that the peak current of BHN‐NTC transistor increased due to the higher carrier injection compared NTC transistor.^[^ ^20^ ^]^ To further demonstrate the enhanced electron injection efficiency of the asymmetric PTCDI‐C13 layer, we conducted a theoretical simulation that helps to understand the electrostatics inside the two device systems using a standard finite‐element numerical solver (ATLAS, Silvaco). This tool solves the coupled Poisson's and drift‐diffusion equations over a discretized mesh structure. We built two metal‐insulator‐semiconductor (MIS) structures that represent the single‐ and bi‐heterojunction transistors in the simulation and calculated the electron concentration (*n*) inside their semiconductor regions at *V*_G_ = −20 V and *V*_D_ = −50 V, the condition that produces strong negative transconductance in both devices.

Figure S7a (Supporting information) shows that, in a single‐heterojunction system, a large energetic barrier between Au and *p*‐type DNTT prevents efficient injection, resulting in extremely low *n* over the DNTT thickness. Eventually, the electric field pointing from the gate to the drain accumulates electrons at the parylene/PTCDI‐C13 interface. The bi‐heterojunction system shows a clearly different trend. Figure S7b (Supporting information) shows that the top *n*‐type PTCDI‐C13 (on the left, in this diagram) improves electron injection from Au forming a first electron accumulation layer at its interface with DNTT. The *n* remains low in DNTT, while its base level is higher than that in Figure S7a (Supporting information). The parylene/PTCDI‐C13 interface is strongly accumulated with electrons with *n* considerably higher than that at the same interface in Figure S7a (Supporting information). Additionally, to further validate the BHN‐NTC transistor mechanism and demonstrate its applicability across a broader range of materials, we fabricated NTC and BHN‐NTC transistors based on PH‐BTBT‐10 and zinc tin oxide (ZTO). As a result, the PH‐BTBT‐10‐ and ZTO‐based BHN‐NTC transistors exhibited expanded NTC regions (Figure S8, Supporting Information). Due to this mechanism, the extended NTC behavior facilitates stable ternary logic operation. Further details are available in the Supporting Information (Figures S9 and S10 and Note S3, Supporting Information). To quantitatively support our findings and compare them with prior studies, we extracted and analyzed key parameters including the NTC region, middle logic length, and middle logic efficiency (Table S1, Supporting Information).

To analyze the impact of improved charge injections on the device, we measured contact resistance using the transmission line method (TLM). For TLM analysis, device structures with ambipolar PTCDI‐C13/DNTT and PTCDI‐C13/DNTT/PTCDI‐C13 junctions were prepared, each consisting of eight transistors with channel lengths ranging from 100 µm to 450 µm in 50 µm increments. This design allowed us to focus on evaluating the effect of introducing the PTCDI‐C13 layer on electron injection (Figure S11, Supporting Information). To extract the contact resistance, transfer characteristics were measured for each device, from which the total resistance (*R*_total_) was calculated and plotted as a function of channel length (*L*). From the linear fit, the contact resistance (2·*R*_c_) was extracted and then normalized by the device width (*W*) to obtain the area‐normalized contact resistance (2·*R*_c_·*W*). In the *n*‐type region measurements (*V*_GS_: −20 to 50 V, step voltage: +1 V), the NTC transistor exhibited a relatively high 2·*R*_c_·*W* = 17.18 MΩ·cm. In contrast, under the same measurement conditions, the BHN‐NTC transistor showed a significantly lower 2·*R*_c_·*W* = 2.44 MΩ·cm (Figure 2e). This reduction in contact resistance, attributed to enhanced electron injection, demonstrated the improved performance of the BHN‐NTC transistor.

In addition to electrical characterization, atomic force microscopy (AFM) and contact angle measurements were conducted to estimate the surface energy of various material configurations, including pristine PTCDI‐C13, pristine DNTT, PTCDI‐C13/DNTT bilayer, and PTCDI‐C13/DNTT/PTCDI‐C13 trilayer structures (Figure S12 and Note S4, Supporting Information). Surface roughness measurements showed a root mean square roughness (R_q_) of 18.48 nm for the NTC transistor and 39.63 nm for the BHN‐NTC transistor, more than doubling in the latter (Figure 2f,g). Increased roughness enhances the contact area with the source and drain electrodes, reducing contact resistance.^[^ ^21^ ^]^ The smoother NTC surface results in higher contact resistance while the rougher BHN‐NTC surface improves charge injection efficiency and lowers contact resistance (Figure 2h,i). To experimentally demonstrate that increased surface roughness enhances both the charge injection area and injection efficiency, three types of buffer transistors were fabricated and electrically characterized (Figure S13 and Note S5, Supporting Information). These findings provided experimental evidence for the complementary mechanisms of injection area expansion and enhanced electron injection via reduced energy barriers.

To further investigate the reliability of these findings, we conducted a uniformity of NTC and BHN‐NTC transistors. Uniformity tests are crucial for assessing the reproducibility and consistency of device characteristics. Figure 2j presents an optical microscopy image(top) and NTC region(bottom), maximum *g*_m_ mapping data(bottom) of BHN‐NTC transistor. We measured the transfer curves of 100 NTC and BHN‐NTC transistors and extracted key parameters such as the NTC region and negative maximum *g*_m_, respectively (Figures S14 and S15, Supporting Information). Through the result, the BHN‐NTC transistor exhibited an average negative maximum *g*_m_ of −2.97 ± 1.03 (10^−8^S) and an average NTC region of 22.3 ± 1.67 V, demonstrating a broader NTC region and higher *g*_m_ compared to the NTC transistor (Figure 2k,l). Figures S16 and S17 (Supporting information) present the device uniformity and long‐term stability of BHN‐NTC transistors utilizing a CYTOP interfacial trap prevention and encapsulation layer, respectively (Figures S16 and S17, Supporting Information). The higher hydrophobicity of CYTOP compared to parylene reduces the influence of interfacial traps and improves air stability. Thus, the CYTOP interfacial trap layer‐based #15 BHN‐NTC transistors exhibited improved uniformity, with Δpeak voltage = 8.7%, Δvalley voltage = 3.4%, and ΔNTC region voltage = 3.87%. In addition, improved air stability was observed in long‐term measurements due to the CYTOP encapsulation layer. These findings suggest that the electrical characteristics of the BHN‐NTC transistor can be selectively engineered through material selection and structural design, depending on specific device and application requirements.

**Figure 3:**
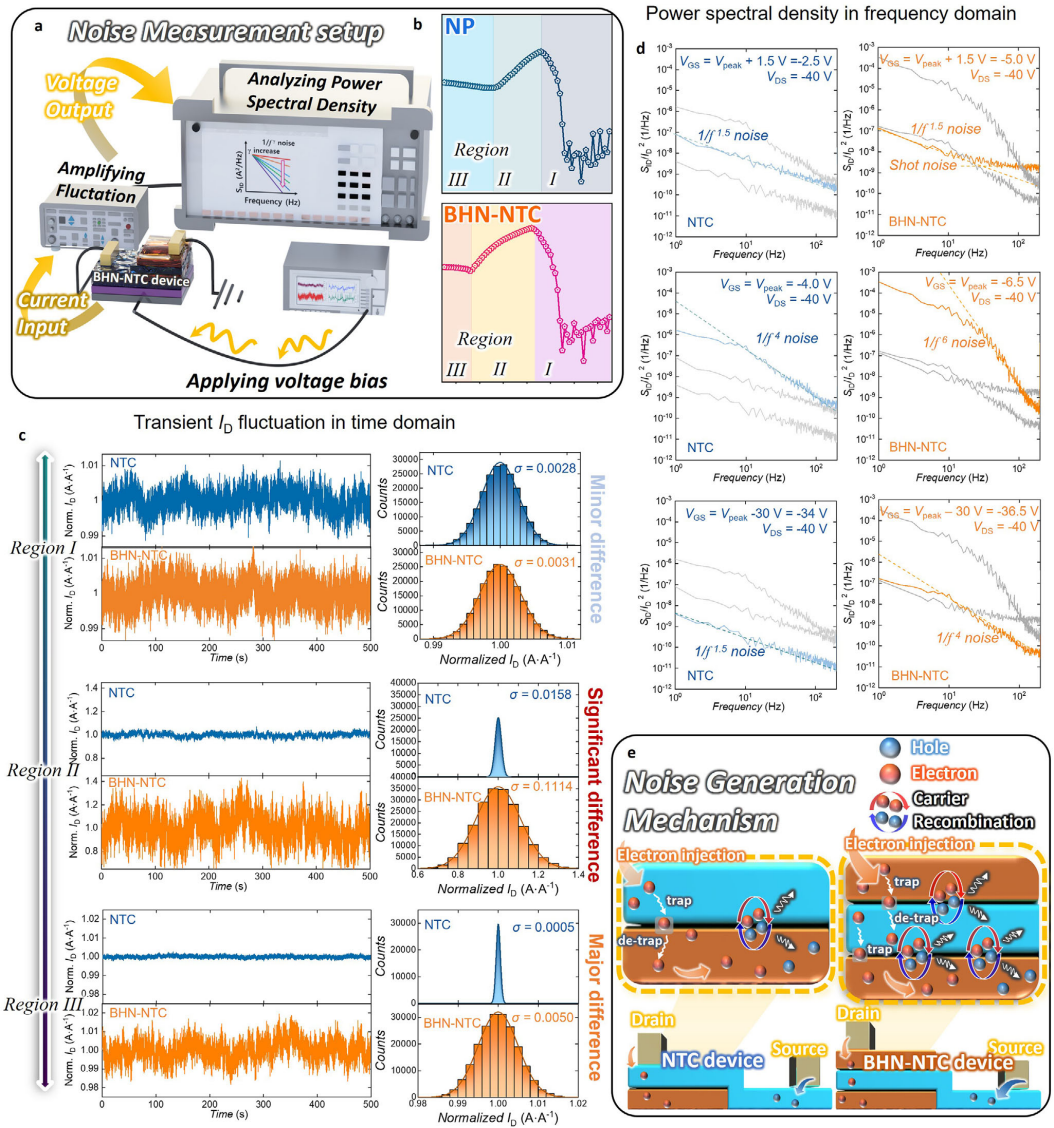
a) Schematic diagram of the LFN measurement setup used to characterize the stochastic behavior of NTC and BHN‐NTC transistors. The measurement system includes a low‐noise amplifier and power spectral density analyzer for accurate time‐domain and frequency‐domain assessments. b) Definition of three operating regions for NTC and BHN‐NTC transistors, facilitating a systematic evaluation of their nonlinear conduction behavior and noise characteristics. c) Time‐domain drains current fluctuations and corresponding Gaussian distributions for NTC and BHN‐NTC transistors. In *Region I*, the noise amplitudes are comparable. However, in *Regions II* and *III*—particularly *Region II*, where the NTC behavior is most prominent—the BHN‐NTC transistor exhibits substantially larger current fluctuations. d) PSD of the NTC and BHN‐NTC transistors across different operating regions, confirming that the LFN of BHN‐NTC transistor is markedly enhanced in *Region II*. e) Schematic illustration of the noise generation mechanism. The interplay between trapping/detrapping and carrier generation/recombination events is more strongly correlated in BHN‐NTC transistors, resulting in significantly increased current noise relative to NTC devices.

As another merit of the proposed device, we explore NTC transistors as a reliable stochastic source for TRNG implementation. Both NTC and BHN‐NTC devices are heterojunction‐based NTC structures, but BHN‐NTC devices incorporate two different heterojunctions, which we expect to enhance noise behavior. This suggests their potential as a robust hardware‐level stochastic source for high‐performance TRNGs. We performed low‐frequency noise (LFN) measurements in both NTC and BHN‐NTC configurations to evaluate the stochastic behavior of the transistors. The experimental setup for LFN characterization is shown in **Figure** **3**a. To systematically analyze the LFN characteristics across the operating region of the NTC transistor, we divided the operation into three regions (Figure 3b). This distinction allows for a comprehensive assessment of the stochasticity of NTC transistors in different operating states. Figure 3c shows the current value distributions of NTC and BHN‐NTC transistors in operating Regions *I*, *II*, and *III*. Note that the current values are obtained from DC transient measurements conducted at a temperature of 25 °C with a sampling rate of 500 Hz over a duration of 500 s. The corresponding distributions of DC current values across these regions are also shown in the left panel. All data points exhibit Gaussian distributions, as indicated by the solid fitting lines. Note that the Gaussian distribution of the current values is particularly advantageous for TRNG implementation, as it ensures a well‐defined and predictable stochastic behavior, which is crucial for generating high‐quality random numbers with minimal bias. In *Region I*, where the drain current monotonically increases with decreasing gate voltage, both NTC and BHN‐NTC transistors display similar current distributions. However, in *Region II*, characterized by the NTC effect, the BHN‐NTC transistor exhibits significantly greater current fluctuations compared to the NTC transistor. Especially, these fluctuations influence electrical parameters such as the subthreshold swing (SS); however, such parameters are not central to our entropy‐generating approach. Instead, the broader NTC region provides a larger and more stable voltage window for noise extraction without adverse effects. This enhanced stochasticity in the BHN‐NTC configuration, combined with its Gaussian nature, provides a robust and reliable random source for TRNG applications. This increased variance in the BHN‐NTC transistor remains evident in *Region III*, highlighting its consistent distinction from the NTC transistor across different operating regions and further reinforcing its utility for reliable hardware‐based randomness generation.

To investigate the origin of the differences in noise magnitude across the operating regions, we analyzed the power spectral density (PSD) of the current fluctuation in the frequency domain by performing a Fourier transform (Figure 3d). The PSD analysis reveals a consistent 1/𝑓^𝑟^ noise (r = −ln(*S*_ID_)/ln(*f*)) characteristic across all operating regions. Note that the LFN of the devices are characterized reliably, which was demonstrated by the repeated PSD measurements (Figure S18, Supporting Information). Conventional transistors generally exhibit 1/𝑓^𝑟^ noise behavior, where the exponent 𝑟 in the PSD slope typically falls within the range of 1 to 2. This noise is primarily attributed to defects either in the gate oxide or within the semiconductor channel.^[^ ^22^ ^]^ Specifically, when a single defect dominates the conduction process, the PSD slope approaches 𝑟 = 2. In contrast, when multiple defects collectively influence the noise without correlation, the PSD represents a superposition of individual contributions, resulting in a slope closer to 𝑟 = 1. Additionally, the bias dependence of noise behavior has been well‐documented in prior studies on transistors.^[^ ^23^ ^]^ As the bias increases, resulting in a higher drain current, the noise magnitude generally decreases. This trend occurs because, at elevated current levels, the influence of a single noise source on overall current fluctuations diminishes proportionally.^[^ ^14a^ ^]^ For NTC transistors, the value of 𝑟 and the magnitude of noise vary significantly across different operating regions, exhibiting distinct behavior from conventional single‐channel transistors previously reported in the literature.^[^ ^23^, ^24^ ^]^ In *Region I*, both NTC and BHN‐NTC transistors display a 1/𝑓 noise characteristic, indicating that the noise originates from defect‐related fluctuation stemming from the gate oxide or the channel, consistent with typical transistor mechanisms.^[^ ^11^ ^]^ In contrast, *Region II* demonstrates a substantial increase in noise magnitude and a change in the slope of the PSD, highlighting a conduction mechanism distinct from conventional transistors. Recent studies suggest that in transistors where channels of different materials form a heterojunction, noise sources from these materials can exhibit correlation effects.^[^ ^4^, ^25^ ^]^ Such correlations can result in higher‐order noise components, unlike superposition of non‐correlated noise sources, which results in a 1/*f* noise. For the NTC and BHN‐NTC devices, the carrier trapping/detrapping (noise source 1) and carrier generation/recombination (noise source 2) can occur at a single defect, resulting in a strong correlation between these noise sources.

These interactions amplify both the noise magnitude and the PSD slope. In the case of BHN‐NTC transistors, the bi‐heterojunction formed by three distinct semiconductor materials introduces even stronger correlations among the noise sources, leading to significantly larger noise components than NTC transistors. This enhanced stochastic behavior is a result of the complex interplay between the multiple heterojunctions.^[^ ^13^, ^25^ ^]^ In *Region III*, as the drain current increases, the correlations among noise sources weaken, resulting in a decrease in both noise magnitude and the PSD slope. This transition highlights the dynamic nature of noise generation and correlation in NTC transistors, providing insight into their unique stochastic properties across different operating regions. In summary, the LFN spectrum can be divided into three regimes depending on their slopes: (i) channel‐scattering regime (1/*f* ^1‐1.5^) whose behavior is characteristic of carrier mobility fluctuations in the channel bulk, (ii) shot‐noise shoulder (1/*f* ^0‐0.5^) whose near‐white noise behavior arises from carrier scatterings at the *p*‐*n* junction barriers, where discrete carrier‐injection events produce weakly frequency‐dependent fluctuations, and (iii) heterojunction‐correlation regime (1/*f* ^4–6^) where the heterojunction trapping/detrapping time constant—the spectrum steepens dramatically, exhibiting a roll‐off between 1/*f* 4 and 1/*f* 6.

Figure 3e presents a schematic diagram of noise source 1 and noise source 2, originating from the heterojunctions of NTC and BHN‐NTC transistors in *Region II*. This illustration highlights that BHN‐NTC transistors, with their bi‐heterojunction structures, generate significantly higher noise levels than NTC transistors. Figures S19–S21 (Supporting information) shows the length‐dependence of LFN in BHN‐NTC transistors, confirming that the enhanced stochastic behavior is intrinsic to the device structure and robust against moderate variation in overlap geometry (Figures S19–S21, Note S6, Supporting Information). Figure S22 (Supporting information) shows device‐to‐device noise variation, confirming that noise characteristics are robust to device‐to‐device variation (Figure S22 and Note S7, Supporting Information).

Inherent high noise that originates from the bi‐heterojunction in an BHN‐NTC transistor is exploited for hardware‐based TRNG implementation. In particular, operating voltage optimization maximizes the trapping/detrapping and carrier generation/recombination processes (*Region II*). In addition, adding a *pn* heterojunction to convert an NTC transistor into an BHN‐NTC transistor through structural optimization maximizes noise. This approach enables the implementation of reliable and efficient hardware‐based TRNG. Note that NTC‐region TRNG studies remain extremely limited; the only prior report relies on shot noise from a single *p*‐*n* junction, necessitating external amplification to achieve even a single bit per sampling event.^[^ ^11^ ^]^ In contrast, our dual‐junction BHN‐NTC transistor expands the NTC window and enhances correlated noise amplitude by more than an order of magnitude, representing the first demonstration of direct multi‐bit entropy extraction from an NTC‐based device without external amplification or conditioning circuits.

**Figure 4:**
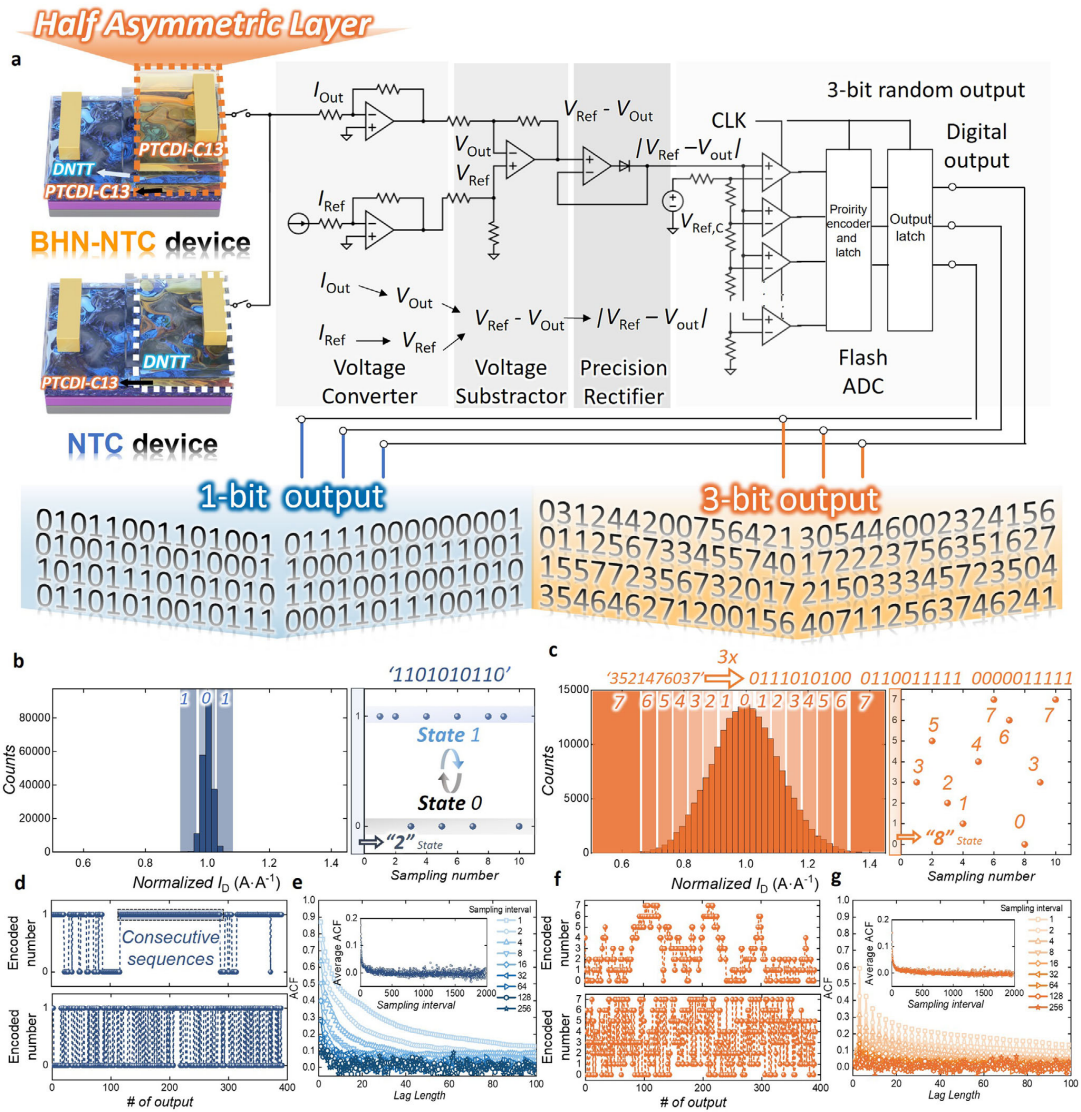
a) Circuit diagram of the hardware‐based TRNG using an NTC, BHN‐NTC transistor. b) Histogram of normalized transient current responses for the NTC transistor configuration measured at 400 Hz (200000 samples) in *Region II*. The right panel shows output states over 10 consecutive sampling events, illustrating state transitions. c) Histogram of normalized transient current responses for the BHN‐NTC transistor configuration measured under identical conditions as in (b). The right panel demonstrates output states across 10 consecutive sampling events, highlighting enhanced state diversity compared to NTC. d) ADC‐encoded outputs of transient current responses for the NTC transistor configuration, generating a 1‐bit random signal under threshold conditions (0.67σ; 0.101). The upper panel shows continuous outputs due to insufficient sampling intervals relative to low‐frequency noise; the lower panel illustrates improved randomness when the sampling interval is increased by a factor of 128. e) ACF analysis for the NTC configuration at various lag lengths. The inset displays average ACF versus sampling interval, indicating a decrease in correlation with increasing interval. f) ADC‐encoded outputs for the BHN‐NTC transistor configuration, generating a 3‐bit random signal under identical threshold conditions as in (d). Similar to (d), the upper panel demonstrates continuous outputs at short sampling intervals, while the lower panel highlights improved randomness at optimized (128×) sampling intervals. g) ACF analysis for the BHN‐NTC configuration at various lag lengths. The inset presents average ACF versus sampling interval, demonstrating significantly faster decorrelation and superior randomness compared to the NTC configuration.

**Figure** **4**a shows the circuit diagram of a hardware‐based TRNG that uses an NTC transistor along with a schematic diagram of the corresponding TRNG output. The noisy current output (*I*_Out_) from the NTC transistor passes through a voltage converter built from an op‐amp and converts into voltage fluctuations (*V*_Out_). At the same time, the reference current (*I*_Ref_) from a current source pass through a voltage converter to produce a reference voltage (*V*_Ref_). A voltage subtractor then produces a voltage signal in the form of *V*_Ref_ – *V*_Out_, and a precision rectifier composed of an op‐amp and a rectifier extracts the absolute value of *V*_Ref_ – *V*_Out_. Next, a *n*‐bit flash ADC converts the absolute value into an *n*‐bit digital signal, where *n* is selected after noise characterization; in our measurements *n* = 1 for the NTC transistor and *n* = 3 for the BHN‑NTC transistor using the same ADC resolution. In this design, increasing the number of bits in the random signal increases the information stored in a fixed‐length bit stream, thereby enabling a TRNG with higher throughput. It is important to note that the flash ADC resolution has limits.^[^ ^26^ ^]^ External noise and voltage margins prevent the resolution from being reduced indefinitely. In practice, high‐resolution ADCs demand more comparators, larger chip area, and higher power consumption, while lower resolutions may restrict the granularity of the randomness and limit throughput. Therefore, under a fixed ADC resolution, it is crucial to increase the noise of the device to increase the number of bits in the random signal.^[^ ^26^ ^]^ Figure 4b,c show histograms of the transient current response for two different NTC transistor structures (Figure 4b for NTC and Figure 4c for BHN‐NTC). A sample frequency of 400 Hz is used, with 200000 samples measured. It is important to note that to enhance TRNG performance, measurements occur in *Region II*—the region where the NTC characteristics emerge at *V*_peak_, as determined from previous low‐frequency noise analysis. In Figure 4b, c the sigma of the normalized current response histogram for the BHN‐NTC configuration is significantly larger than that for the NTC configuration. This difference occurs because the BHN‐NTC configuration includes one additional heterojunction interface compared to the NTC configuration. The large fluctuation enables the implementation of four times (2–8 states) as many states with a flash ADC of fixed resolution. Consequently, if the NTC transistor produces a 1‐bit random signal, the BHN‐NTC configuration produces a 3‐bit random signal. When this 3‐bit signal is converted into a binary bitstream, the throughput becomes three times larger over the same time interval. The right panel of Figures 4b,c shows the output states over 10 consecutive sampling events. Figures 4d,f show the output encoded by a flash ADC of fixed resolution for the measured transient current responses of the NTC (Figure 4d) and BHN‐NTC (Figure 4f) configurations. The ADC resolution is determined based on the NTC transistor generating a 1‐bit output. Based on measurements indicating that the transient current follows a normal distribution, the threshold parameter “a” is set so that the probability of a measurement falling between –a and a is 50%. Specifically, if the measured value deviates by less than 0.67σ (*a* = 0.101) from the mean, the ADC outputs 0; if it deviates more, the output is 1. For a fair comparison, the same threshold value is applied to the BHN‐NTC configuration; however, because the sigma in the BHN‐NTC configuration is significantly larger, the ADC partitions the state into 3‐bits rather than the 1‐bit output observed in the NTC configuration. The graph in the upper panel of Figures 4d,f demonstrates that both configurations produce continuous output sequences because the sampling time is too short relative to the low‐frequency noise, causing the state to remain unchanged before a new sample is captured. Therefore, optimizing the sampling time is necessary. In Figures 4d and f, the lower panels show the output when the sampling interval is increased by a factor of 128 relative to the upper panels. Adjusting the sampling time to match the time constant at which low‐frequency noise is maximized increases the randomness of the signal. Figures 4e,g show the autocorrelation function (ACF) calculated for various lag lengths as a function of the sampling interval for both NTC (Figure 4e) and BHN‐NTC (Figure 4g) configurations.

The ACF of a time series is defined as follow:^[^ ^27^ ^]^

(1) $$\mathrm{ACF}\;\left(k\right)=\frac{\sum_{t\;=1}^{N-k}\left(x_{t}-\mu \right)\left(x_{t+k}-\mu \right)}{\sum_{t=1}^{N}{\left(x_{t}-\mu \right)}^{2}}$$

where *k* denotes the lag (i.e., the number of time steps by which the series is shifted), *x*_t_ denotes the value of the time series at time *t*, *µ* denotes the meaning of the time series, and *N* denotes the total number of samples in the time series. In both cases, increasing the sampling interval causes the ACF to approach zero across all lag lengths. Specifically, when the 3‐bit signal is converted to a 1‐bit signal (see Figure 4e,g) repeated identical inputs at low sampling intervals create a periodic pattern with a period of three bits. This periodic repetition causes elevated ACF values at lag multiples of three. However, as the sampling interval increases, the influence of this periodic behavior diminishes, and the ACF converges to zero for all lag lengths. The inset in Figure 4e, g shows the relationship between the average ACF (across all lag lengths) and the sampling interval. The NTC configuration reaches an ACF below 0.01 at a sampling interval of 100, whereas the BHN‐NTC configuration reaches an ACF below 0.01 at a sampling interval of 50. This finding indicates that the BHN‐NTC configuration can operate with a shorter sampling interval.

Building on the noise‐to‐random value analysis, the BHN‐NTC configuration not only generates a 3‐bit random output rippling the throughput compared to a 1‐bit output at the same sampling interval—but also operates at a shorter sampling interval, enabling faster random signal generation. **Table** **1** presents the NIST test results for the BHN‐NTC configuration, where the transient current response was encoded as a 3‐bit output and later converted to 1‐bit across various sampling intervals. As the sampling interval increases, the number of passed tests also rises. At a sampling step of 500, the system successfully passes all 13 NIST tests applicable within the available signal length, demonstrating strong randomness. The BHN architecture not only forms the dual N‐P‐N stack needed for three‐bit entropy extraction, but it also simultaneously lowers the electron‐injection barrier and expands the NTC window. The higher electron flux that now meets the hole flux at the heterojunction increases the number of defects experiencing simultaneous trapping and recombination, while the wider bias range keeps the device in this correlated regime for a larger portion of its operating swing. Together these effects broaden the current‐fluctuation envelope that the flash‐ADC digitizes, allowing the transistor to deliver a large, correlation‐amplified noise signal—and hence three independent random bits—without any external gain circuitry. In our BHN‐NTC transistor, the large stochastic noise is generated by correlated carrier trapping and recombination within the N‐P‐N heterojunction, not from applying a high electric field. The ±20–30 V bias used in this study is required only because of the thick (20 nm) parylene gate dielectric. Replacing that layer with a thin high‐κ oxide—or an all‐inorganic stack—would lower the operating voltage without reducing the noise. Because the correlation amplifies the fluctuation inside the device, no external analog gain stage is needed, allowing the TRNG to operate at low supply voltage and low power. To further enhance these noise‐amplifying effects, bulk heterojunctions—where *p*‐type and *n*‐type materials form an interpenetrating network throughout the active layer—offer a dramatically increased interfacial area and a more uniform distribution of trap and recombination sites compared to planar heterojunctions. Such a volumetric interface could, in principle, further amplify correlated noise processes by providing a higher density of interconnected noise sources, potentially leading to even stronger stochastic behavior in NTC transistors.

**Table 1:** NIST test results of the TRNG output obtained by converting the transient noise measured in *Region II* of the BHN‐NTC device into 3‐bit data.

NIST 800–22	P‐value	Result
Frequency	0.035080	Pass
Block Frequency	0.129389	Pass
Runs	0.341969	Pass
Longest Runs	0.427959	Pass
Matrix Rank	0.412076	Pass
Discrete Fourier Transform (DFT)	0.811152	Pass
Non‐Overlapping Template	0.999983	Pass
Overlapping Template	Not enough length	
Maurer's Universal Statistical Test	Not enough length	
Linear Complexity	Not enough length	
Serial	0.250380	Pass
Approximate Entropy	0.241119	Pass
Cumulative Sums (Forward)	0.065373	Pass
Cumulative Sums (Backward)	0.065373	Pass
Random Excursions	0.085020	Pass
Random Excursions Variant	0.097254	Pass

**Figure 5:**
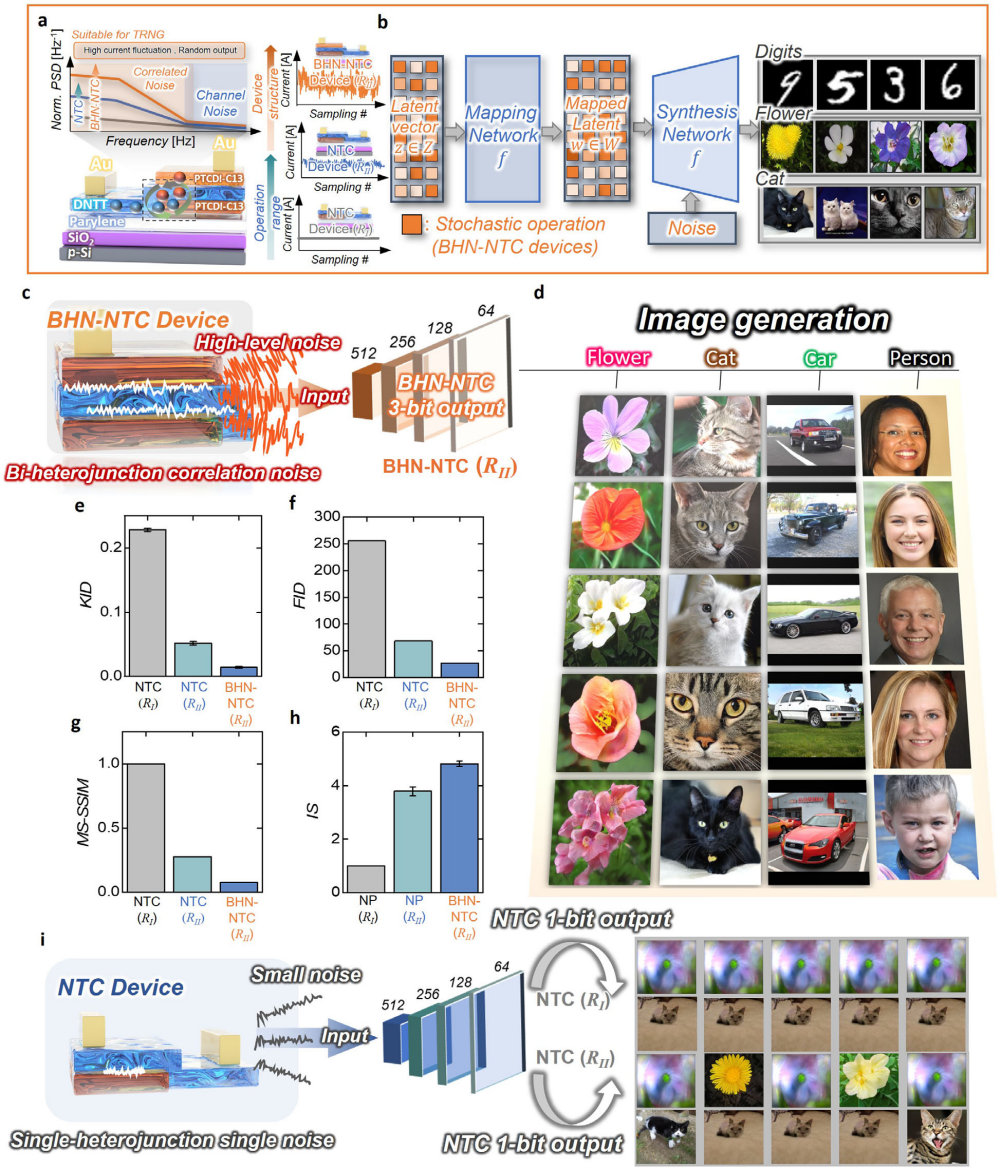
a) Schematic diagram of two noise enhancement approaches: one optimizing the operating region of an NTC transistor in *Region II*, and the other converting an NTC transistor into an BHN‐NTC transistor by introducing an additional NTC heterojunction to further amplify noise. b) Schematic diagram of the GAN architecture for image generation. c) Schematic diagram of image generation using an BHN‐NTC transistor in StyleGAN2, along with representative generated images. d) Generated images of flowers, cats, cars, and people using BHN‐NTC devices in StyleGAN2 demonstrate that higher noise levels can produce a diverse set of high‐quality images that are virtually indistinguishable from real ones. e) KID scores comparing image similarity for NTC and BHN‐NTC devices in *Regions I* and *II*, f) FID scores quantifying proximity to real image distribution for NTC and BHN‐NTC devices in *Regions I* and *II*, g) MS‐SSIM values assessing image diversity for NTC and BHN‐NTC devices in *Regions I* and *II*, and h) IS values evaluating image quality and diversity for NTC and BHN‐NTC devices in *Regions I* and *II*. i) Schematic diagram and generated results from image synthesis using an NTC‐based TRNG in StyleGAN2, showing that the lower noise level in the configuration results in low‐quality, indistinguishable images that are different from real images.

The following shows the process of image generation, utilizing the inherent random noise from the heterojunctions in NTC transistors. The generation of high‐quality images using generative models relies critically on the randomness and diversity of input latent vectors.^[^ ^28^ ^]^ Insufficient randomness in latent vectors can result in detrimental outcomes such as mode collapse, where generated images become repetitive and lack variability, or biased image distributions that inaccurately represent the target dataset. However, generating sufficiently random latent vectors directly from hardware‐based TRNG poses substantial practical challenges. High‐dimensional latent vectors require extensive hardware resources, making full hardware implementation demanding due to factors such as increased device complexity, higher power consumption, and scalability limitations. To address these issues, we optimized both the structure and operational conditions of a NTC transistor. The TRNG output is fed into a StyleGAN2 network for the generation of a variety of images, including flowers and cats, with a focus on comparing the performance differences between the NTC and BHN‐NTC configurations. **Figure** **5**a shows a schematic diagram of two innovative approaches for noise enhancement. One approach optimizes the operating region of an NTC transistor by analyzing its low‐frequency noise, selecting *Region II*, where NTC characteristics are maximized and trapping/detrapping as well as carrier generation/recombination processes yield the highest noise level. The other approach employs device structure optimization, converting an NTC transistor into a BHN‐NTC transistor by introducing an additional NTC heterojunction. This structural transformation further amplifies the correlation between the trapping/detrapping and carrier generation/recombination processes, thereby significantly enhancing the overall noise. Figure 5b presents a schematic diagram of GAN architecture. To validate our proposed hardware‐based TRNG approach, we conducted a Python‐based simulation. Specifically, the 512‐dimensional latent vector required by StyleGAN2 is constructed by directly using the encoded transient responses from the BHN‐NTC transistors as the random noise input seeding the GAN. The continuous transient noise is first quantized into discrete levels, forming a digital latent vector that is then fed into the StyleGAN2 generator. The code further loads a pretrained StyleGAN2 model using a legacy loader, processes the latent vectors in batches, and synthesizes images. This entire pipeline, based on actual device measurement results, is implemented in Python and integrated with PyTorch and torchvision, demonstrating the effective integration of hardware‐based random number generation into the image synthesis process. The performance of the TRNG is evaluated by comparing different operating regions (*Region I* and *Region II* in the NTC device) as well as different device structures (NTC and BHN‐NTC devices operating in *Region II*). First, Figure 5c shows a schematic of image generation where the noise source from *Region II* of the BHN‐NTC device is converted to a 3‐bit output and utilized as input to StyleGAN2. The BHN‐NTC‐based TRNG produces randomized potential vectors with significantly higher noise amplitudes, and this increased noise can be converted into a high quality and highly variable image at the GAN output, successfully generating images that are virtually indistinguishable from the real image. Using the BHN‐NTC TRNG as input, we can see that the system generates image elements of flower, cat, car, and person with remarkable quality (Figure 5d). We further extracted four metrics to evaluate the quality and diversity of the generated images: Fréchet inception distance (FID), kernel inception distance (KID), inception score (IS), and multi‐scale structural similarity (MS‐SSIM). Figure 5e shows the KID scores for images generated under different device structures (NTC and BHN‐NTC) and operating regions (*Region I* and *Region II*), where KID measures the kernel‐based disagreement between image features—lower values are preferable. Figure 5f presents the FID, which quantifies the distance between the generated images and the true image distribution, with lower values indicating better performance. Figure 5g displays the MS‐SSIM, assessing the structural similarity between images; lower values denote higher diversity. Finally, Figure 5h illustrates the IS, which evaluates both the quality and diversity of the images generated, with higher scores favoring greater diversity.^[^ ^29^ ^]^ Figure 5i shows a schematic diagram of image generation using an NTC transistor in StyleGAN2, along with the generated image results. In the NTC transistor operating in *Region I*, the latent vectors generated are nearly zero‐valued; as a result, the StyleGAN2 network produces almost identical images and even experiences model collapse, yielding anomalous outputs. In contrast, when the NTC device operates in *Region II*, the latent vectors exhibit higher noise levels and are more diverse than those in *Region I*, although overlapping images and occasional model collapse are still observed, indicating that the noise level remains insufficient for consistently high‐quality image generation. Therefore, the results in Figure 5e–i, we confirmed that BHN‐NTC (*Region II*) > NTC (*Region I*) > NTC (*Region I*) on all metrics, confirming that the higher noise level in the BHN‐NTC structure enables a more effective exploration of the latent space and results in a higher throughput of diverse images. These findings demonstrate that stable image generation is achieved not only in the cat domain but also in the various domains (Figure S23, Supporting Information), indicating that the BHN‐NTC based image generator is applicable across a wide range of domains. We evaluated various metrics to quantify the quality of the generated images in different domains, as shown in (Figure S24, Supporting Information), demonstrating that the stochasticity from the BHN‐NTC transistors in operating *Region II* obtained the best performance in all domains. The applicability of the stochastic BHN‐NTC transistor‐based image generation is further demonstrated in the MNIST dataset (Figure S25, Supporting Information).

## 3 Conclusion

In summary, this study demonstrates that exploiting the inherent noise in heterojunction‐based NTC transistors can enable a reliable hardware‐based TRNG that markedly improves GAN‐based image generation. By integrating hardware‐derived randomness with advanced image synthesis techniques, the work illustrates broader potential for robust stochastic computing methods that enhance both quality and throughput in various application domains. The proposed BHN‐NTC transistor featured an asymmetric *n*‐type layer atop a conventional NTC transistor, and it offered two key advantages:

1. First, this structure maximizes electron injection efficiency. In conventional *p*‐type NTC transistors, electron injection from the drain is severely limited. By introducing an asymmetric *n*‐type semiconductor, we significantly improved electron injection, reducing 2·*R*_c_·W from 17.18 to 2.44 MΩ·cm. As a result, the NTC region expanded from 19 to 27 V, and negative *g*_m_ improved by −0.036 to −0.073 µS, confirming enhanced electrical performance.
2. Second, the introduction of additional *pn* junctions enables the realization of TRNG. While conventional NTC transistors exhibit a 1/*f* ^1.5^ noise profile due to weak carrier recombination/generation and trap/de‐trap processes occurring at a single *pn* junction, our BHN‐NTC transistors display an extremely high noise level of 1/*f*.^6^ This leads to considerable current fluctuations, providing the foundation for TRNG with a highly uniform Gaussian distribution in *Region II*, where current fluctuation differences are maximized. The stochastic performance of the BHN‐NTC transistor was demonstrated through NIST test, confirming its suitability as TRNG. When we sample these noise sources, we can generate eight distinct output states (0 to 7) in a 3‐bit system, unlike the 1‐bit system (0 to 1) produced by an NTC transistor. Extended output characteristics of BHN‐NTC transistors can be used as an image generator that receives a noise source as input. Furthermore, the output characteristics of the BHN‐NTC with powerful random noise characteristics can be used to generate various high‐quality images that are difficult to distinguish from the real image. Reflecting the above multi‐bit random numbers, we utilized the noise of BHN‐NTC transistors to generate high‐quality images. The image generation parameters, including KID, FID, MS‐SSIM, and IS, showed significant improvement, demonstrated that BHN‐NTC transistors enable high‐quality image generation compared with conventional NTC transistor noise image generation.

Based on these results, we believe that the proposed BHN‐NTC transistor not only significantly improves the electrical characteristics of conventional NTC transistors by optimizing charge injections but also demonstrates strong potential for hardware‐based reliable TRNG for robust image generation technologies.

## 4 Experimental Section

PTCDI‐C13 was purchased from Sigma‐Aldrich for thermal deposition. DNTT was purchased from DAEYEON CHEMICALS for thermal deposition. Parylene (dix‐SR) was purchased from KISCO for chemical vapor deposition (CVD).

### Fabrication of NTC Transistor

2.5 by 2.5 cm Si/SiO_2_ substrate was cleaned by ultrasonic cleaning with acetone and isopropanol (IPA) for 10 min and the Si is utilized as a back gate. The substrate was cleaned with deionized water and blown with N_2_ gas to remove any remaining water and residue. After blowing substrate, the parylene was fully coated at 20 nm as an insulating film (Obang Technology (Gimpo, Korea)). PTCDI‐C13 and DNTT were sequentially deposited on the fully coated parylene by thermal evaporation using a thermal evaporator as 30 and 20 nm (deposition rates were 0.1–0.3 and 0.1‐0.2 Ås^−1^, respectively). Au electrode was deposited using a thermal evaporator (deposition rate was 0.8–1.0 Ås^−1^) and the thickness was 70 nm.

### Fabrication of BHN‐NTC Transistor

2.5 by 2.5 cm Si/SiO_2_ substrate was cleaned by ultrasonic cleaning with acetone and isopropanol (IPA) for 10 min and the Si is utilized as a back gate. The substrate was cleaned with deionized water and blown with N_2_ gas to remove any remaining water and residue. After blowing substrate, the parylene was fully coated at 20 nm as an insulating film (Obang Technology (Gimpo, Korea)). PTCDI‐C13 and DNTT were sequentially deposited on the fully coated parylene by thermal evaporation using a thermal evaporator as 30 and 20 nm (deposition rates were 0.1–0.3 and 0.1–0.2 Ås^−1^, respectively). Subsequently, additional PTCDI‐C13 was deposited by thermal evaporation using a thermal evaporator as 30 nm (deposition rate was 0.1–0.3 Ås^−1^). Au electrode was deposited using a thermal evaporator (deposition rate was 0.8–1.0 Ås^−1^) and the thickness was 70 nm.

### Device Characterization

The electrical characteristics of all devices and stability were measured using a probe station and a Keithley 4200A‐SCS analyzer under an ambient atmosphere.

### Morphology, Band Structure and Contact Angle Analysis, and Elemental Analysis

The morphologies of PTCDI‐C13/DNTT, PTCDI‐C13/DNTT/PTCDI‐C13 were analyzed using an atomic force microscopy (AFM, Park NX10, Park system, Suwon, KOREA). Drop shape analyzer with a CM4200 lens modules was used to measure the contact angle of PTCDI‐C13/DNTT/PTCDI‐C13, PTCDI‐C13/DNTT, DNTT, PTCDI‐C13 (Contact angle, DSA25, Kruss, State of North Carolina, USA). The elemental analysis of PTCDI‐C13, DNTT were measured by X‐ray photoelectron spectroscopy (Figure S26, Supporting Information) (XPS, AXIS SUPRA, Kratos. Inc., California, USA) at the Yonsei Center for Research Facilities). Band diagrams of each material were analyzed with UV‐visible spectroscopy (UV–Vis, Lambda 750, PerkinElmer, The commonwealth of Massachusetts, USA).

### LFN Measurement

To systematically examine the stochastic behavior of both NTC and BHN‐NTC transistors, we performed LFN measurements at 25 °C using. The measurement setup included a low‐noise amplifier and filtering system (SR570) to accurately capture current fluctuations in the time domain. Subsequently, we applied a Fourier transform to the collected data to obtain the PSD using 35670A and analyzed its slope to evaluate the underlying noise mechanisms. By categorizing the operating bias into three distinct regions (*Region I*, *II*, *III*), we could investigate the changes in noise amplitude, correlation, and conduction pathways, which directly reveal the distinct stochastic properties of the NTC and BHN‐NTC transistors under different biases.

### TRNG Simulation

For TRNG implementation, the noisy drain current (*I*_noise_) generated by the NTC and BHN‐NTC transistor was converted into a voltage signal (*V*_out_) using an operational amplifier‐based circuit. A reference current (*I*_ref_) was similarly converted to a reference voltage (*V*_ref_), and a subtractor plus a precision rectifier stage were employed to produce a unipolar output that serves as the input to an *n*‐bit flash ADC. The experimentally recorded drain‑current traces were used as the input stimulus in a SPICE model that includes the current‑to‑voltage converters, differential subtractor, precision rectifier, and flash‑ADC. We acquired *I*_noise_, varying the sampling interval to optimize randomness. The output bit streams were evaluated via autocorrelation function (ACF) analysis and NIST statistical tests, which together quantify the randomness quality and confirm that the BHN‐NTC device in the NTC regime delivers enhanced noise suitable for high‐throughput TRNG applications.

### GAN Simulation

To demonstrate the applicability of our hardware‐based TRNG, we integrated it with a StyleGAN2 framework to generate images of flowers, cats, and other domains. The measured transient current signals were digitized to create 512‐dimensional latent vectors, which were then fed into the generator network of StyleGAN2. We assessed image quality and diversity using four metrics—Fréchet inception distance (FID), kernel inception distance (KID), inception score (IS), and multi‐scale structural similarity (MS‐SSIM). By comparing the outputs from different device configurations (NTC and BHN‐NTC) and operating regions (*Region I*, *II*, and *III*), we confirmed that higher noise amplitudes yield improved latent‐space exploration, producing high‐quality, diverse images with minimal risk of mode collapse.

## Conflict of Interest

The authors declare no conflict of interest.

## Supporting information



Supporting Information

## Data Availability

The data that support the findings of this study are available from the corresponding author upon reasonable request.
