# Supplementary material for: Heterojunction‐Driven Stochasticity: Bi‐Heterojunction Noise‐Enhanced Negative Transconductance Transistor in Image Generation
Source: Adv Mater. 2025 Jun 25;37(41):2505150. doi: 10.1002/adma.202505150 (PMC12531724; doi:10.1002/adma.202505150)
Supplement: Supplementary file 1 — Supporting Information [file ADMA-37-2505150-s001.docx]

Supporting Information

**Heterojunction-Driven Stochasticity: Bi-Heterojunction Noise-Enhanced Negative Transconductance Transistor in Image Generation**

Youngmin Han^1^, Ryun-Han Koo^2,3^, Jaechan Song^4^ , Chang-Hyun Kim^7 *^ Eun Kwang Lee^5,*^, Wonjun Shin^,6,*^, and Hocheon Yoo^1,*^

- Supplementary Figure -

**Figure S1.** Comparative investigation of the electrical characteristics, OM image, structure schematics, and equivalent circuit schematics of (a) AAT and (b) BHN-NTC transistors for understanding of the charge transport mechanism and electrical behavior in BHN-NTC transistors. **p. 48**

**Figure S2.** (a) Concept diagram of NTC transistor operating in *Region* *I, II and III* including valley current and peak current. Conceptual realization of NTC curves in (b) NTC and (c) BHN-NTC transistor using 3 type of devices. **p. 49**

**Figure S3.** Transconductance characteristics of (a) NTC (b) BHN-NTC transistor in *p*-type operating range. **p. 50**

**Figure S4.** Schematic energy band diagrams constructed based on UPS measurements of three DNTT and three PTCDI-C13 samples, used to investigate the mechanism of enhanced electron injection in the BHN-NTC transistor. **p. 51**

**Figure S5.** UV-vis measurement data of (a) PTCDI-C13 and (b) DNTT for bandgap extraction.

**p. 52**

**Figure S6.** Arrhenius plots of (a) NTC and (b) BHN-NTC transistors under varying *V*_GS_ at a fixed drain voltage (*V*_DS_ = -50 V). The extracted electron injection barrier (Φ_B_) is significantly reduced in the BHN-NTC structure due to the incorporation of an additional PTCDI-C13 layer, indicating improved electron injection efficiency. **p. 53**

**Figure S7.** Theoretically calculated electron concentration (*n*) inside the semiconductor region of the metal-insulator-semiconductor (MIS) structure included in the (a) single-heterojunction and (b) bi heterojunction negative transconductance transistor. Positions of all layers are drawn for illustrative purposes (thicknesses not to scale). **p. 54**

**Figure S8.** Schematic diagram and transfer curves of NTC and BHN-NTC transistors fabricated with different semiconductor materials to evaluate the general applicability of the BHN-NTC mechanism: (a, c) organic *p*-type semiconductor Ph-BTBT-10 based device (black line = Ph-BTBT-10-based NTC transistor, blue line = Ph-BTBT-10-based BHN-NTC transistor) and (b, d) oxide *n*-type semiconductor ZTO based device (black line = ZTO-based NTC transistor, red line = ZTO-based BHN-NTC transistor). The Ph-BTBT-10-based devices were fabricated by replacing DNTT, and the ZTO-based devices were fabricated by replacing the lower PTCDI-C13 layer. **p. 55**

**Figure S9.** Transfer curves of (a) PTCDI-C13 single transistors (b) DNTT single transistors with linear and log scale. **p. 56**

**Figure S10.** Ternary inverter circuits using NTC and BHN-NTC transistors as a PMOS. (a) VTC curves of ternary inverter utilizing NTC transistor as PMOS and PTCDI-C13 as the NMOS. (b) VTC curves of ternary inverter utilizing an BHN-NTC transistors as the PMOS and PTCDI-C13 as the NMOS. **p. 57**

**Figure S11.** TLM analysis to investigate the electron injection using ambipolar structure. (a) OM image of PTCDI-C13/DNTT TLM analysis and transfer curves. (b) OM image of PTCDI-C13/DNTT/PTCDI-C13 TLM analysis and transfer curves. **p. 58**

**Figure S12.** Contact angle analysis with deionized water and formamide of (a) BHN-NTC transistor, (b) NTC transistor, (c) DNTT single transistor, (d) PTCDI-C13 single structure.
 **p. 59**

**Figure S13.** Device schematics and electrical characteristics of PTCDI-C13 channel-based single and buffered transistors (DNTT and PTCDI-C13/DNTT hybrid buffers) for validation of the effect of the asymmetric PTCDI-C13 electron injection buffer layer. **p. 60**

**Figure S14.** 100 transfer curves of NTC transistors to evaluate the device uniformity, and we extract *g*_m_ and NTC region. **p. 61-64**

**Figure S15.** 100 transfer curves of BHN-NTC transistors to evaluate the device uniformity, and we extract *g*_m_ and NTC region.  **p. 65-68

Figure S16.** (a) 15 transfer curves of CYTOP interfacial trap prevention layer based BHN-NTC transistors to investigate the device uniformity, and we extract (b) peak voltage (c) peak current (d) valley voltage (e) valley current (f) NTC region voltage (g) on current. **p. 69

Figure S17.** The comparative analysis of BHN-NTC transistors with and without a CYTOP encapsulation layer was conducted. Schematic diagram of the BHN-NTC transistor (a) with a CYTOP (b) without CYTOP encapsulation layer uniformly covering the electrodes and semiconductor channel. Transfer curves of the BHN-NTC transistor (c) with a CYTOP (d) without CYTOP encapsulation layer measured at two-day intervals over a 11-day period. **p. 70**

**Figure S18.** Measured *S*_ID_/*I*_D_^2^ versus frequency graphs for NTC and BHN-NTC devices across different operating regions, collected from five distinct devices. (a) NTC, *Region* *I*, (b) NTC, *Region II*, (c) NTC, *Region III*, (d) BHN-NTC, *Region I*, BHN-NTC, *Region II*, (f) BHN-NTC, *Region III*. **p. 71**

**Figure S19.** (a) Schematic cross-sections illustrating two device configurations (#1 and #2) with a fixed bottom PTCDI-C13 layer length of 200 μm, comparing overlapped (#1) versus non-overlapped (#2) top PTCDI-C13 layer geometries. (b) Transfer curves (*I*_D_-*V*_GS_) of devices #1 and #2. (c) Normalized current noise spectra (*S*_ID_/*I*_D_^2^) of devices #1 and #2. **p. 72**  **Figure S20.** (a) Schematic cross-sections of devices (#3 and #4) with a bottom PTCDI-C13 length of 400 μm, comparing overlapped (#3) and non-overlapped (#4) top layers. (b) Transfer curves (*I*_D_-*V*_GS_) of devices #3 and #4. (c) Normalized current noise spectra (*S*_ID_/*I*_D_^2^) of devices #3 and #4. **p. 73**
**Figure S21.** (a) Device schematics for a larger bottom PTCDI-C13 length of 600 μm, comparing overlapped (#5) and non-overlapped (#6) configurations. (b) Transfer curves (*I*_D_-*V*_GS_) of devices #5 and #6. (c) Normalized current noise spectra (*S*_ID_/*I*_D_^2^) of devices #5 and #6.

**p. 74**
 **Figure S22.** Device-to-device consistency of noise characteristics. Top panel: *S*_ID_/*I*_D_^2^ spectra measured in *Region II* for five randomly chosen BHN-NTC transistors fabricated in the same run; despite visible differences in stripe overlap, the curves differ only slightly over the entire frequency range. Bottom panel: histograms of the 500 Hz transient drain-current traces recorded at the same bias for the same five devices; Gaussian fits give σ values of 0.119, 0.126, 0.118, 0.123, and 0.127, demonstrating that the current-fluctuation window varies by roughly ten percent across the set. The similarity in both frequency and time domains confirms that modest edge offsets introduced during fabrication do not materially affect the excess noise as long as the N-P-N junction is present. **p. 75

Figure S23.** Examples of images generated in various domains using the read noise from NTC *Region I*, NTC *Region II*, and BHN-NTC *Region II* as latent vectors. (a) Cat, (b) Flower, (c) Human, (d) Car. **p. 76**
**Figure S24.** Various evaluation metrics (KID, FID, MS-SSIM, IS) for 10,000 images generated in different domains using the read noise from NTC *Region I*, NTC *Region II*, and BHN-NTC *Region II* as latent vectors. (a) Flower, (b) Human, (c) Car. **p. 77

Figure S25.** (a) G model and D model losses over training epochs. (b) FD scores as a function of the epoch for NTC(R*_I_*), NTC(*R_II_*), and BHN-NTC(*R_II_*). (c) Classification results of the generated images. (d) Progression of the generated images over training epochs for each device.

**p. 78**

**Figure S26.** XPS analysis of (a) PTCDI-C13 (b) DNTT. **p. 79**

- Supplementary Table -

**Table S1.** Comparison table of NTC-based transistors and their corresponding implementation results in multi-valued logic (MVL) applications.  **p. 80**

**Table S2.** Benchmark of representative hardware TRNGs for contextualizing the present work and comparing device performance with prior studies. **p. 81**

- Supplementary Note -

**Note S1:** Analysis of the anti-ambipolar transistor conducted to understand the operating characteristics of the NTC and BHN-NTC. **p. 82, 83**

**Note S2:** Analysis of energy barrier differences based on Arrhenius plot analysis. **p. 84**

**Note S3:** Investigation of the extended NTC region and implications for ternary logic applications.  **p.85**

**Note S4:** Contact angle measurement for influence of additional PTCDI-C13 layer using OWRK model. **p. 86, 87**

**Note S5:** Increased surface roughness–induced expansion of contact area, improved charge injection efficiency, and reduced contact resistance. **p. 88**

**Note S6:** Length-dependence of low-frequency noise in BHN-NTC transistors. **p. 89, 90**

**Note S7:** Device-to-device noise variation. **p. 91**


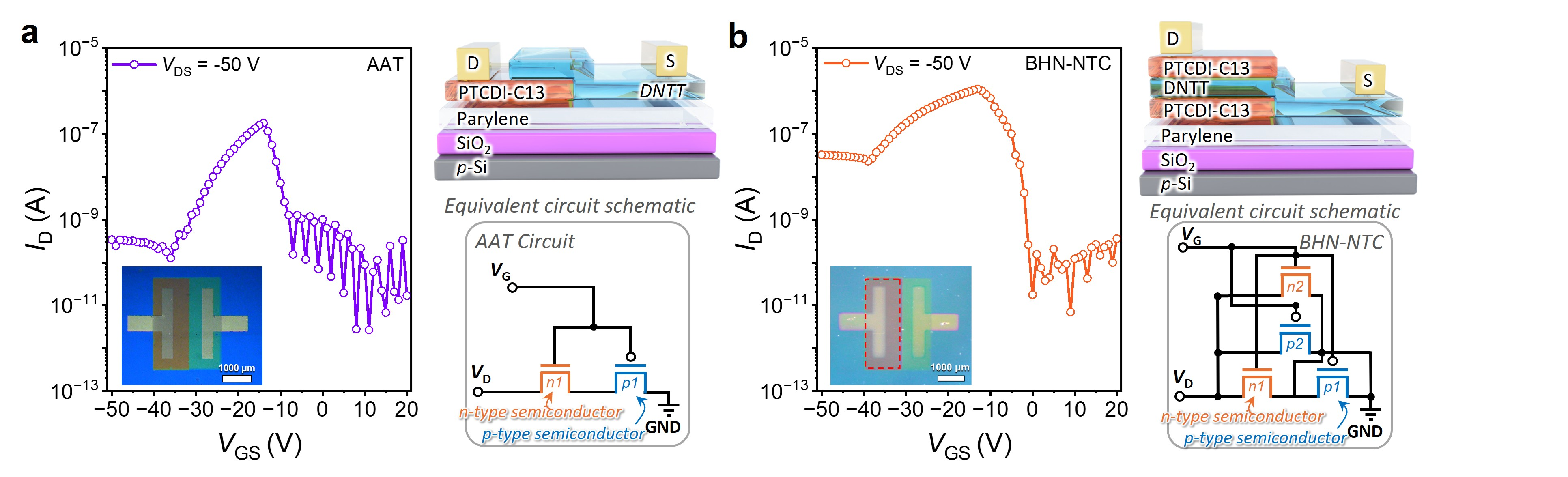


**Figure S1.** Comparative investigation of the electrical characteristics, OM image, structure schematics, and equivalent circuit schematics of (a) AAT and (b) BHN-NTC transistors for understanding of the charge transport mechanism and electrical behavior in BHN-NTC transistors.


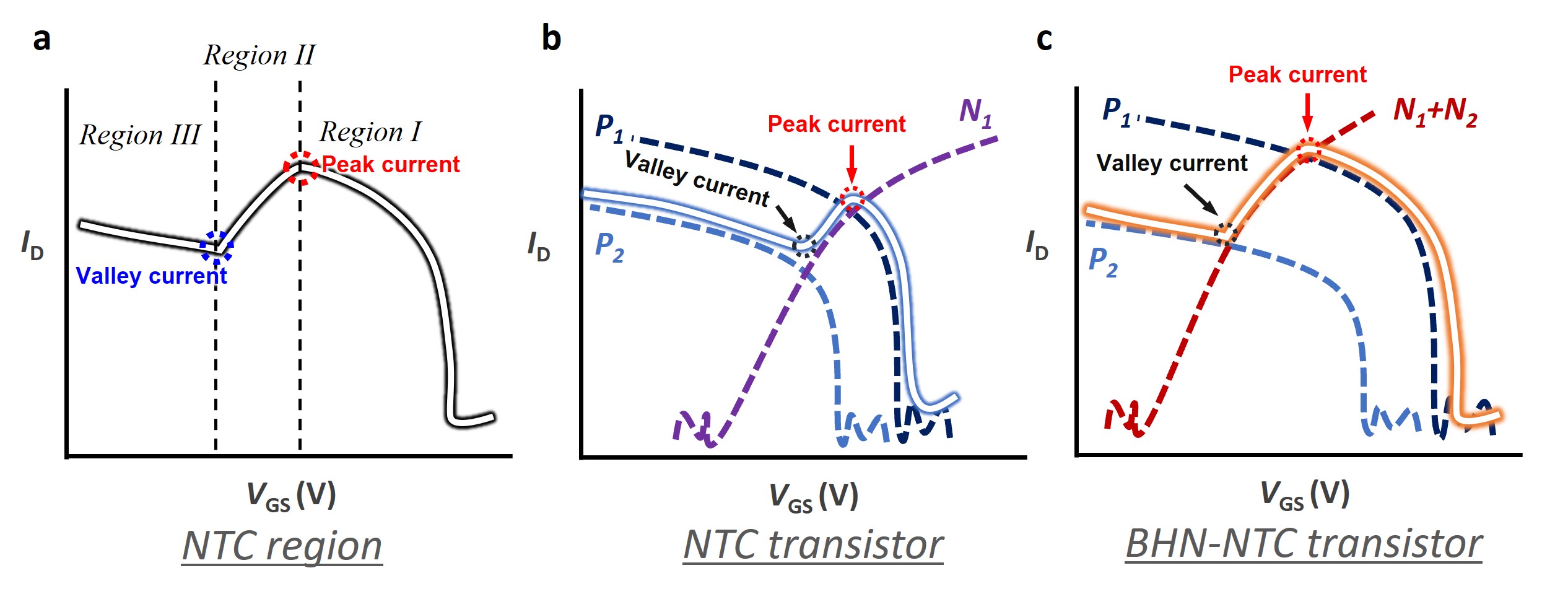


**Figure S2.** (a) Concept diagram of NTC transistor operating in *Region* *I, II and III* including valley current and peak current. Conceptual realization of NTC curves in (b) NTC and (c) BHN-NTC transistor using 3 type of devices.


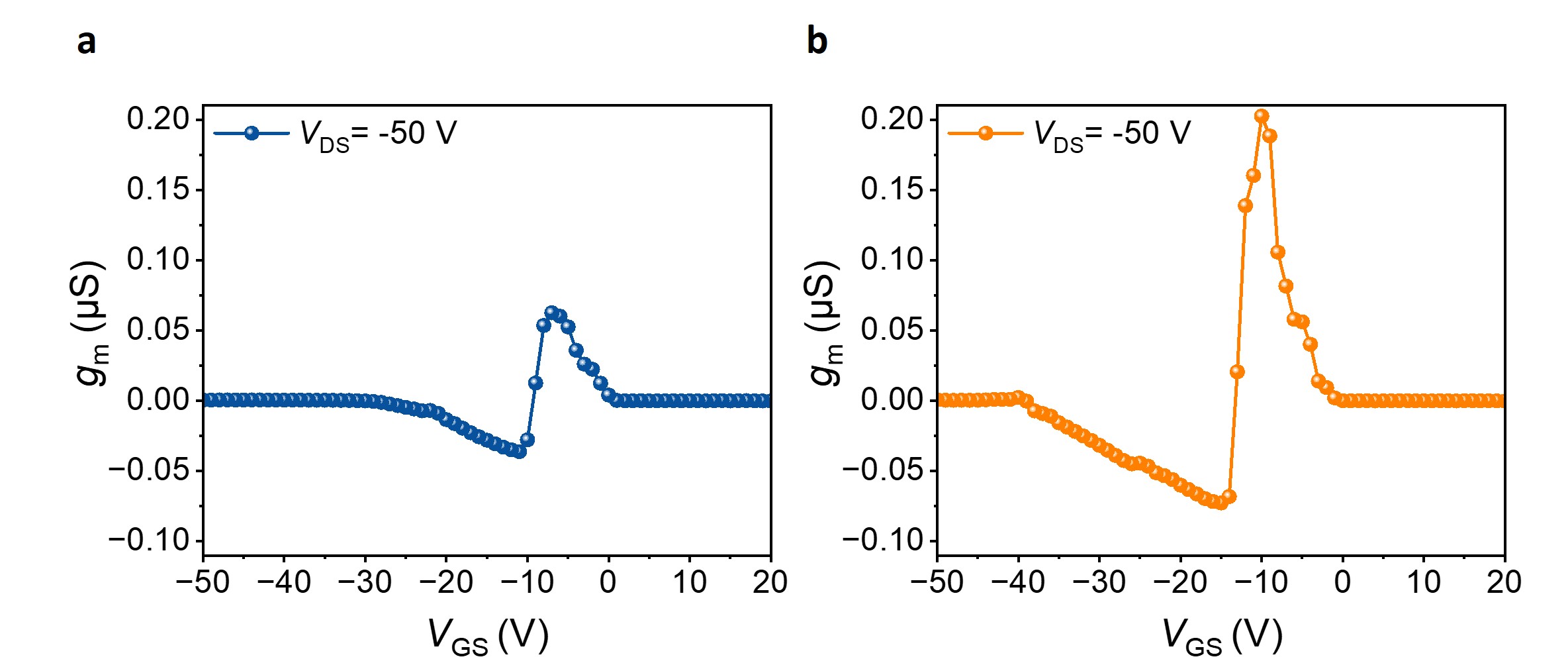


**Figure S3.** Transconductance characteristics of (a) NTC (b) BHN-NTC transistor in *p*-type operating range.

**
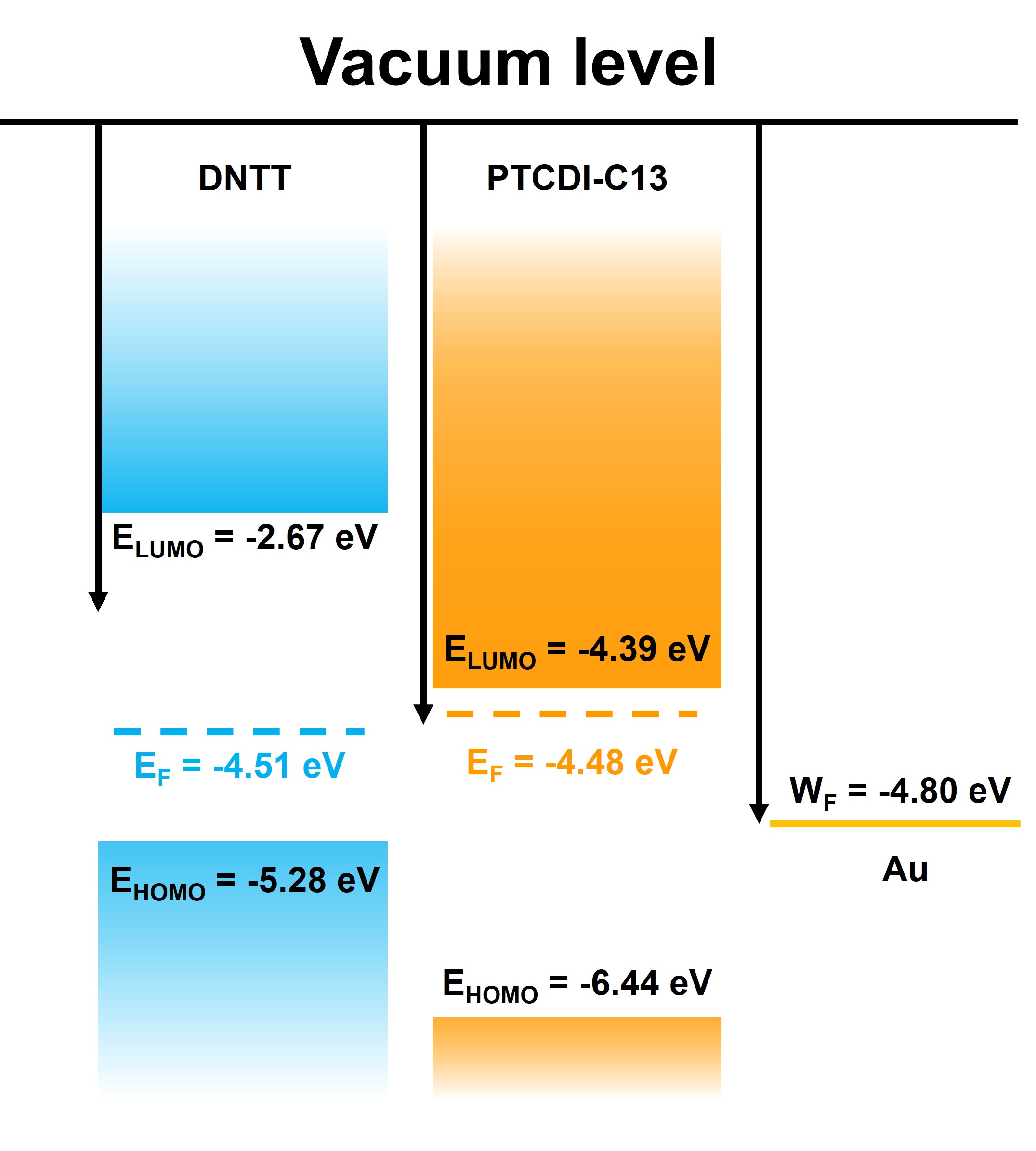
**

**Figure S4.** Schematic energy band diagrams constructed based on UPS measurements of three DNTT and three PTCDI-C13 samples, used to investigate the mechanism of enhanced electron injection in the BHN-NTC transistor.


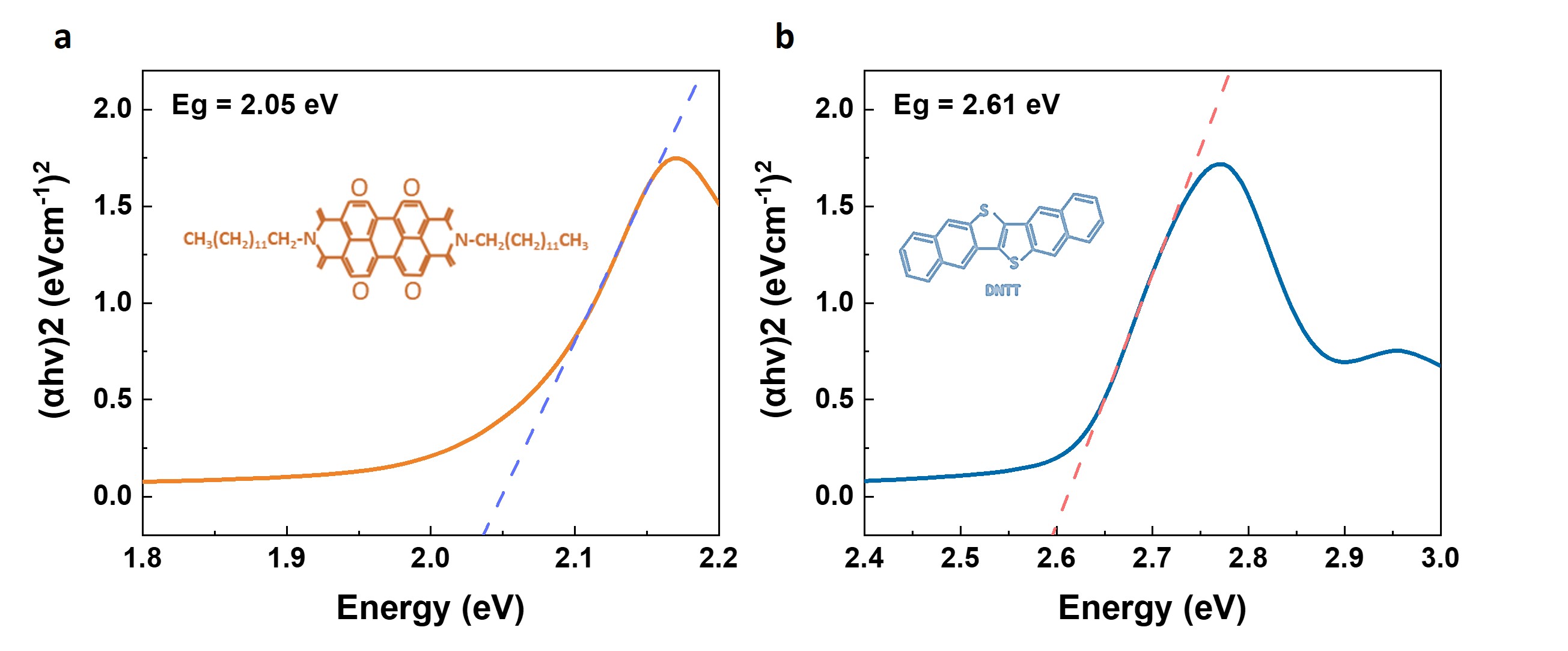


**Figure S5.** UV-vis measurement data of (a) PTCDI-C13 and (b) DNTT for bandgap extraction.


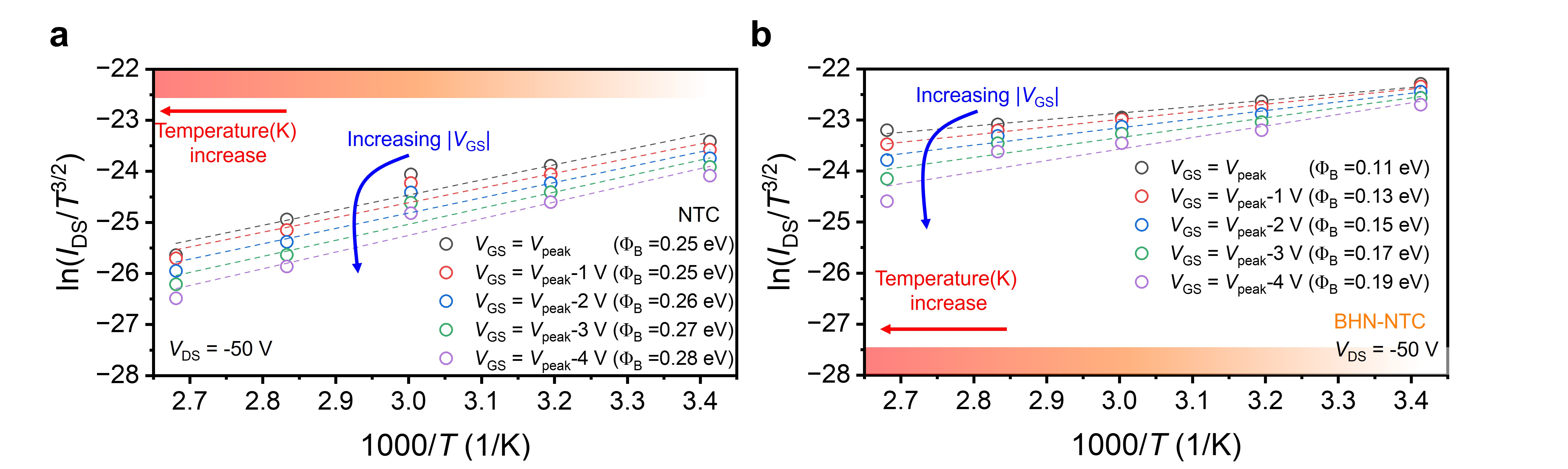


**Figure S6.** Arrhenius plots of (a) NTC and (b) BHN-NTC transistors under varying *V*_GS_ at a fixed drain voltage (*V*_DS_ = -50 V). The extracted electron injection barrier (Φ_B_) is significantly reduced in the BHN-NTC structure due to the incorporation of an additional PTCDI-C13 layer, indicating improved electron injection efficiency.


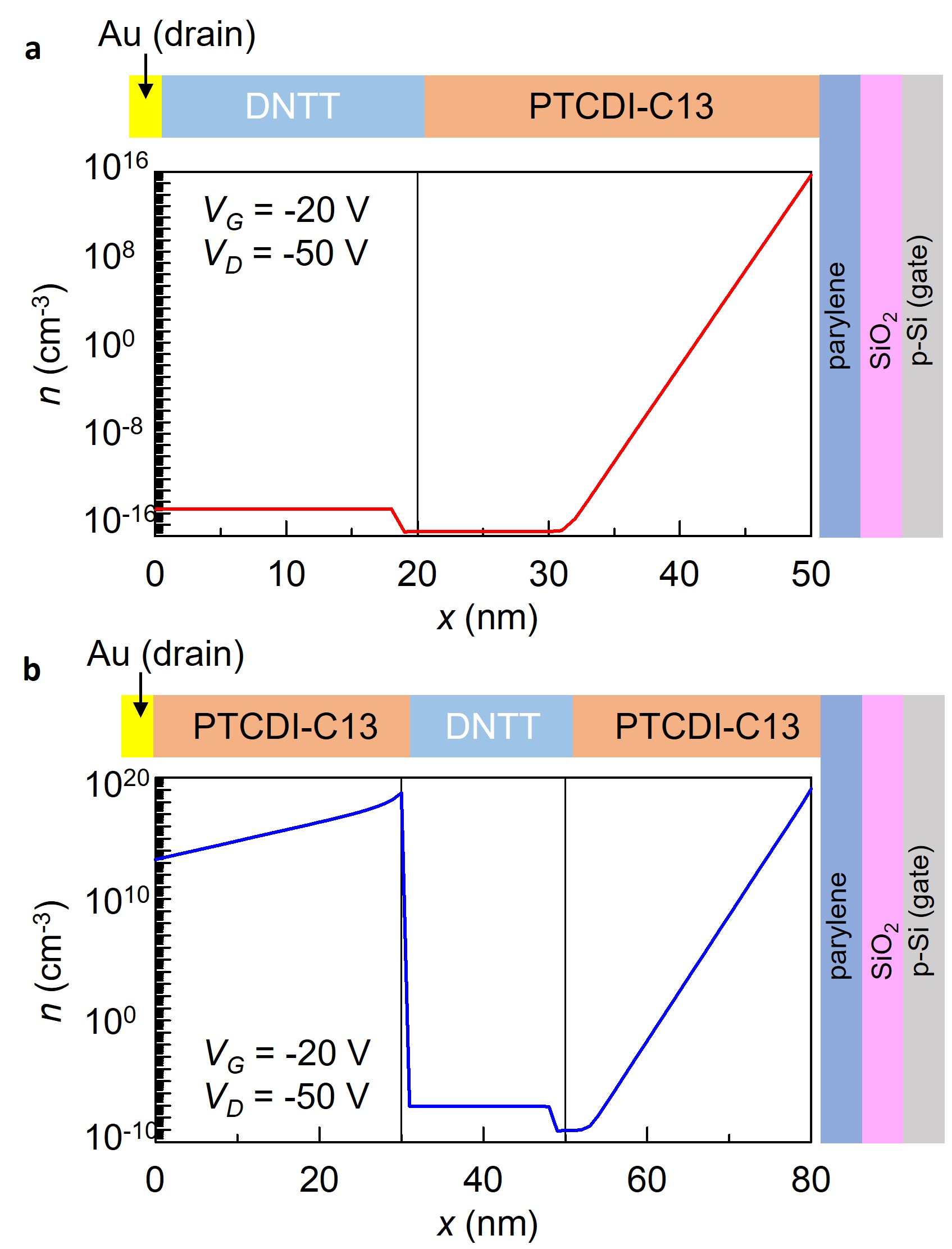


**Figure S7.** Theoretically calculated electron concentration (*n*) inside the semiconductor region of the metal-insulator-semiconductor (MIS) structure included in the (a) single-heterojunction and (b) bi heterojunction negative transconductance transistor. Positions of all layers are drawn for illustrative purposes (thicknesses not to scale).


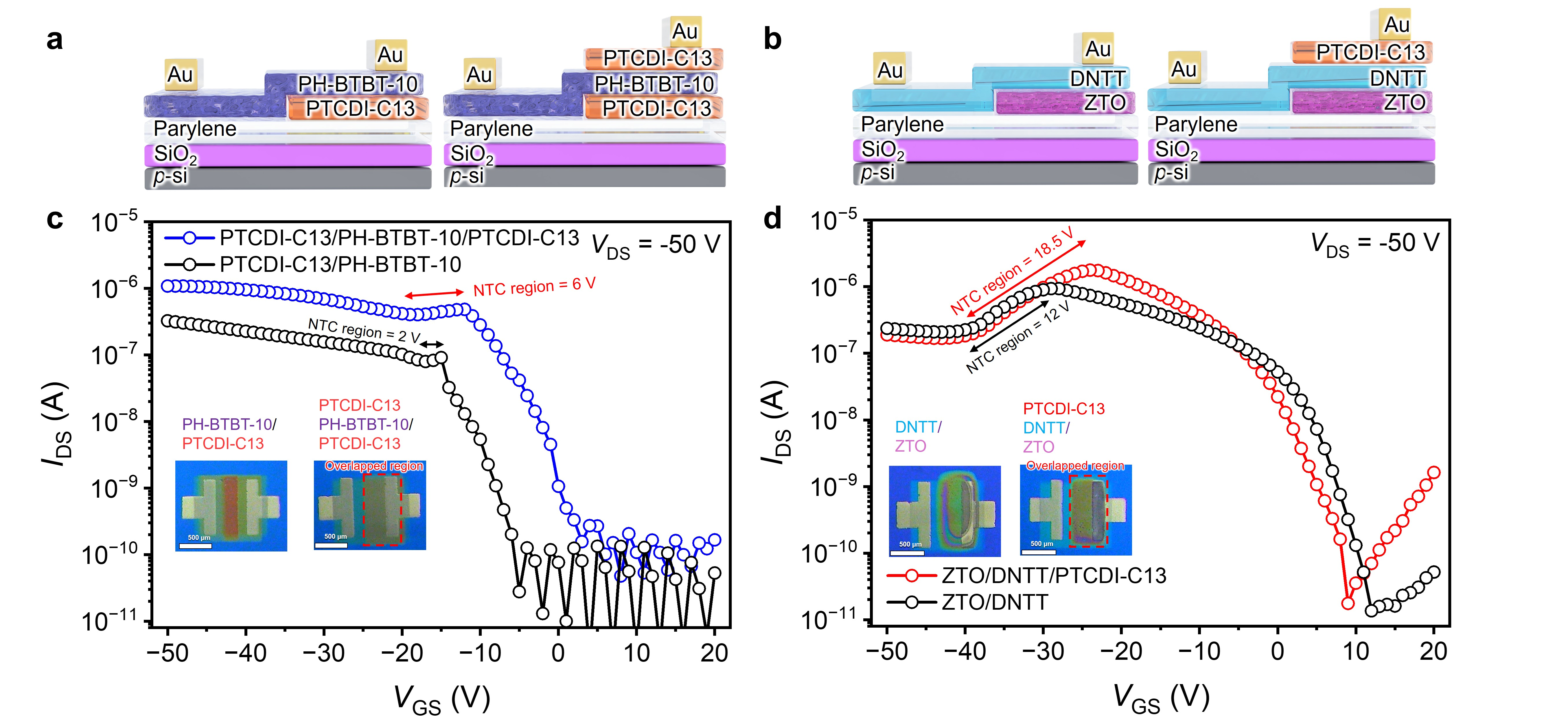


**Figure S8.** Schematic diagram and transfer curves of NTC and BHN-NTC transistors fabricated with different semiconductor materials to evaluate the general applicability of the BHN-NTC mechanism: (a, c) organic *p*-type semiconductor Ph-BTBT-10 based device (black line = Ph-BTBT-10-based NTC transistor, blue line = Ph-BTBT-10-based BHN-NTC transistor) and (b, d) oxide *n*-type semiconductor ZTO based device (black line = ZTO-based NTC transistor, red line = ZTO-based BHN-NTC transistor). The Ph-BTBT-10-based devices were fabricated by replacing DNTT, and the ZTO-based devices were fabricated by replacing the lower PTCDI-C13 layer.


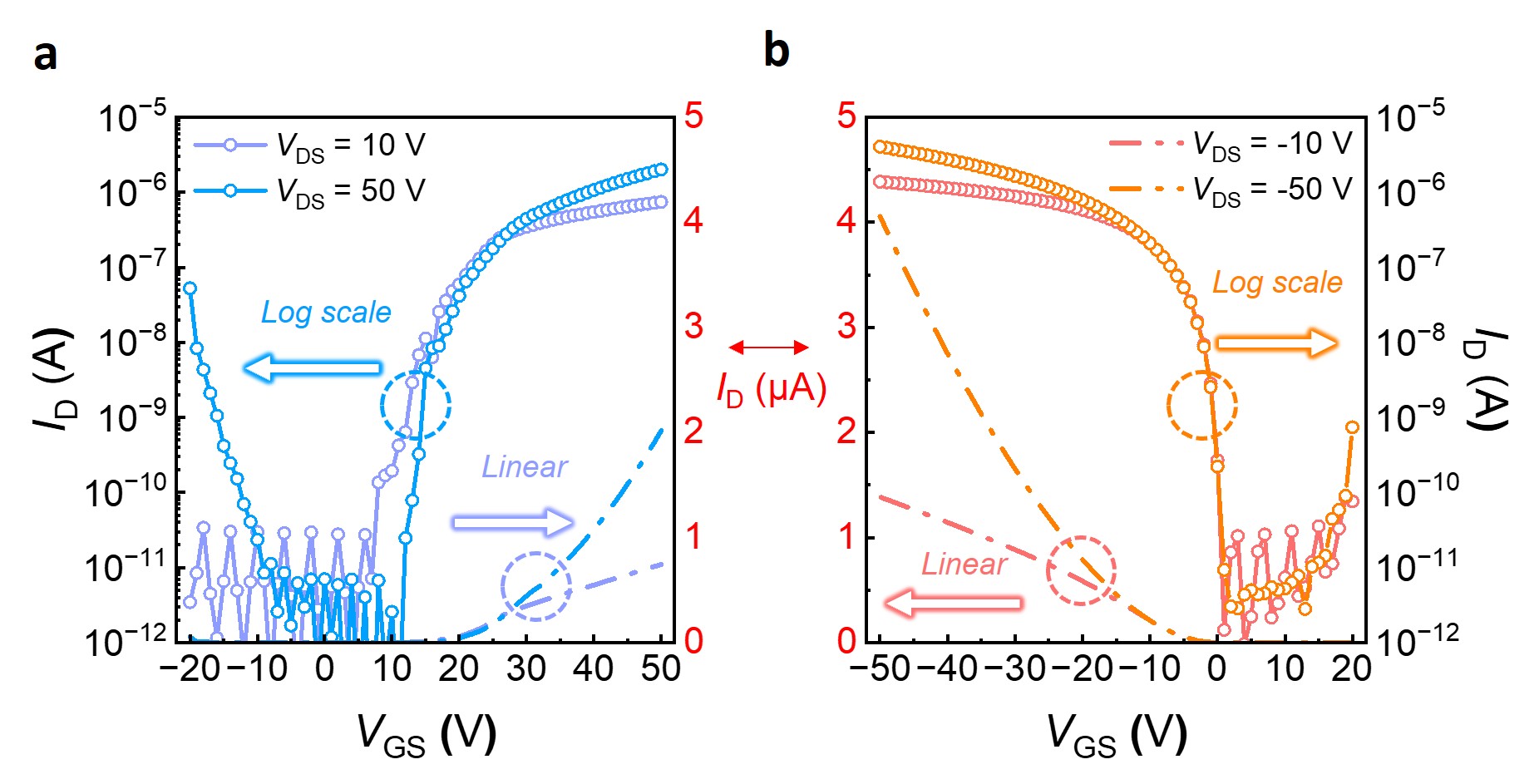


**Figure S9.** Transfer curves of (a) PTCDI-C13 single transistors (b) DNTT single transistors with linear and log scale.


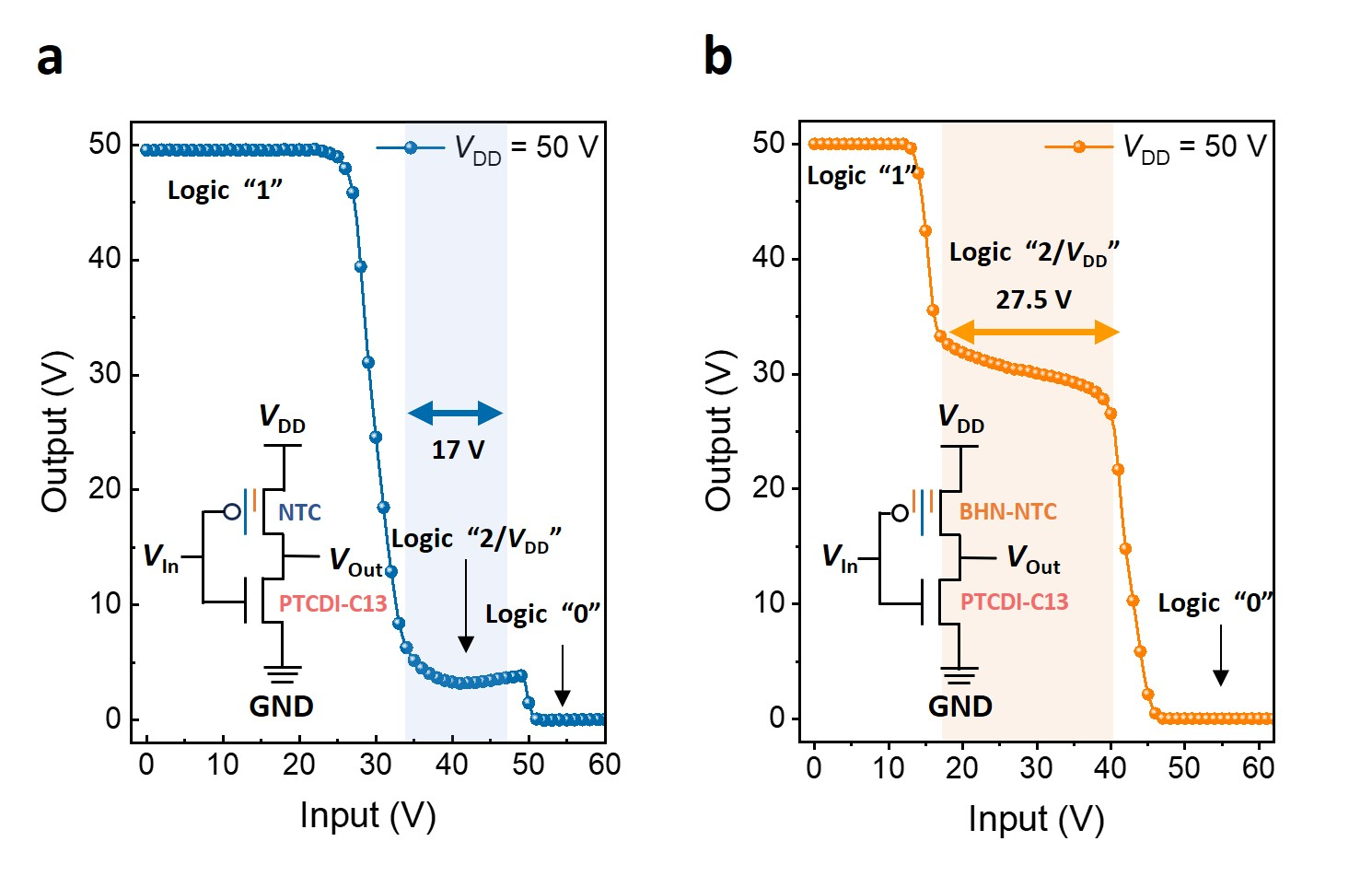


**Figure S10.** Ternary inverter circuits using NTC and BHN-NTC transistors as a PMOS. (a) VTC curves of ternary inverter utilizing NTC transistor as PMOS and PTCDI-C13 as the NMOS. (b) VTC curves of ternary inverter utilizing an BHN-NTC transistors as the PMOS and PTCDI-C13 as the NMOS.


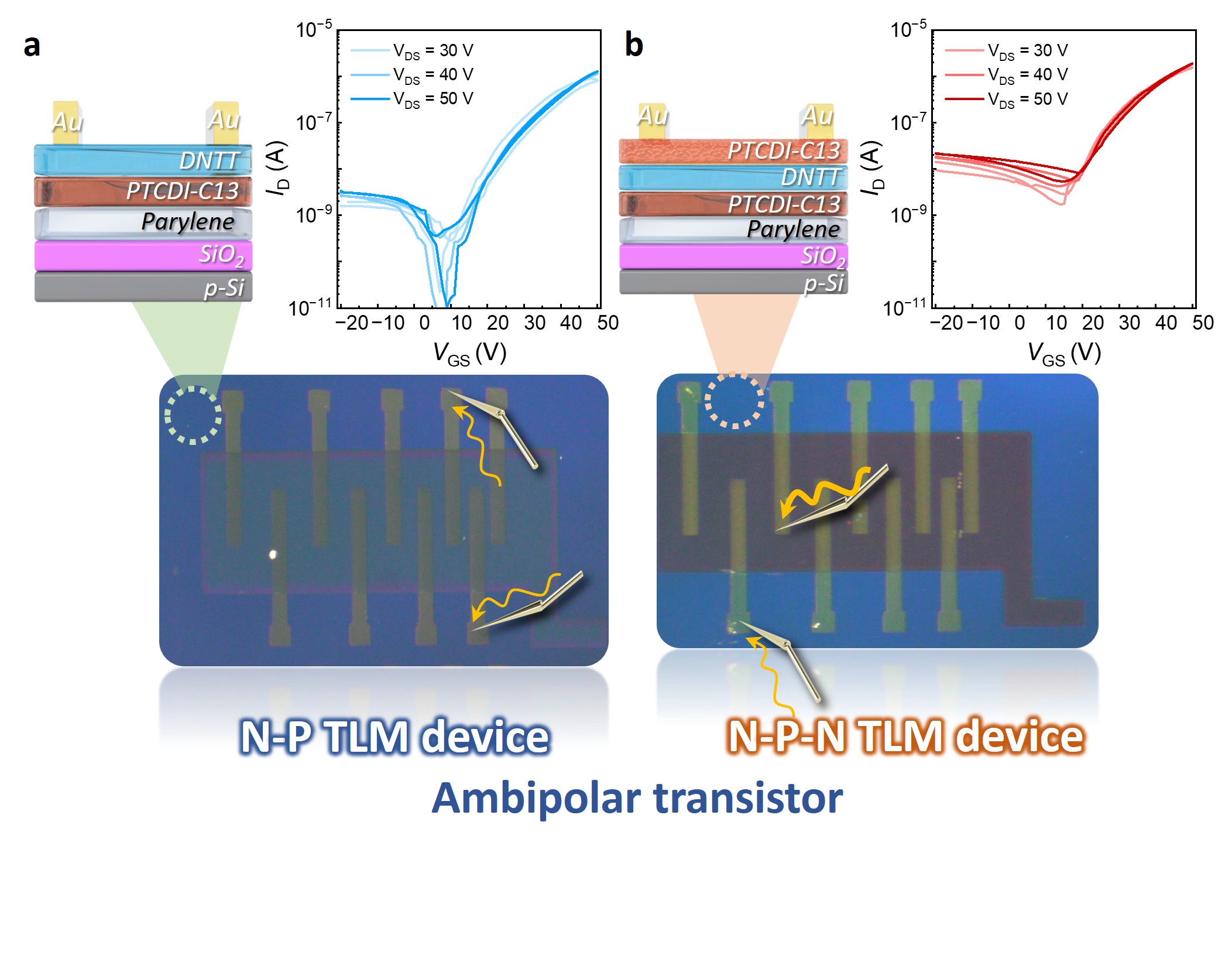


**Figure S11.** TLM analysis to investigate the electron injection using ambipolar structure. (a) OM image of PTCDI-C13/DNTT TLM analysis and transfer curves. (b) OM image of PTCDI-C13/DNTT/PTCDI-C13 TLM analysis and transfer curves.


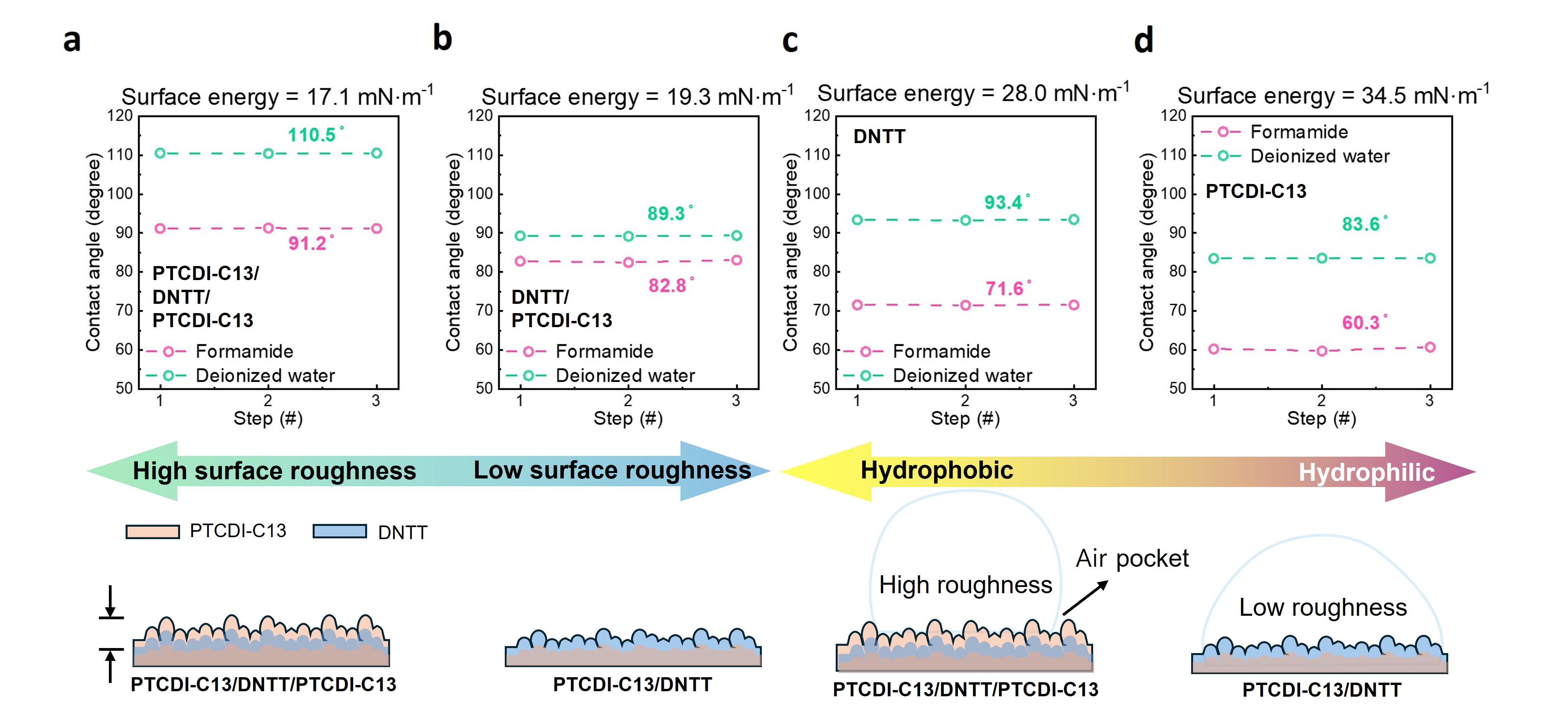


**Figure S12.** Contact angle analysis with deionized water and formamide of (a) BHN-NTC structure, (b) NTC structure, (c) DNTT single structure, (d) PTCDI-C13 singles structure.

**
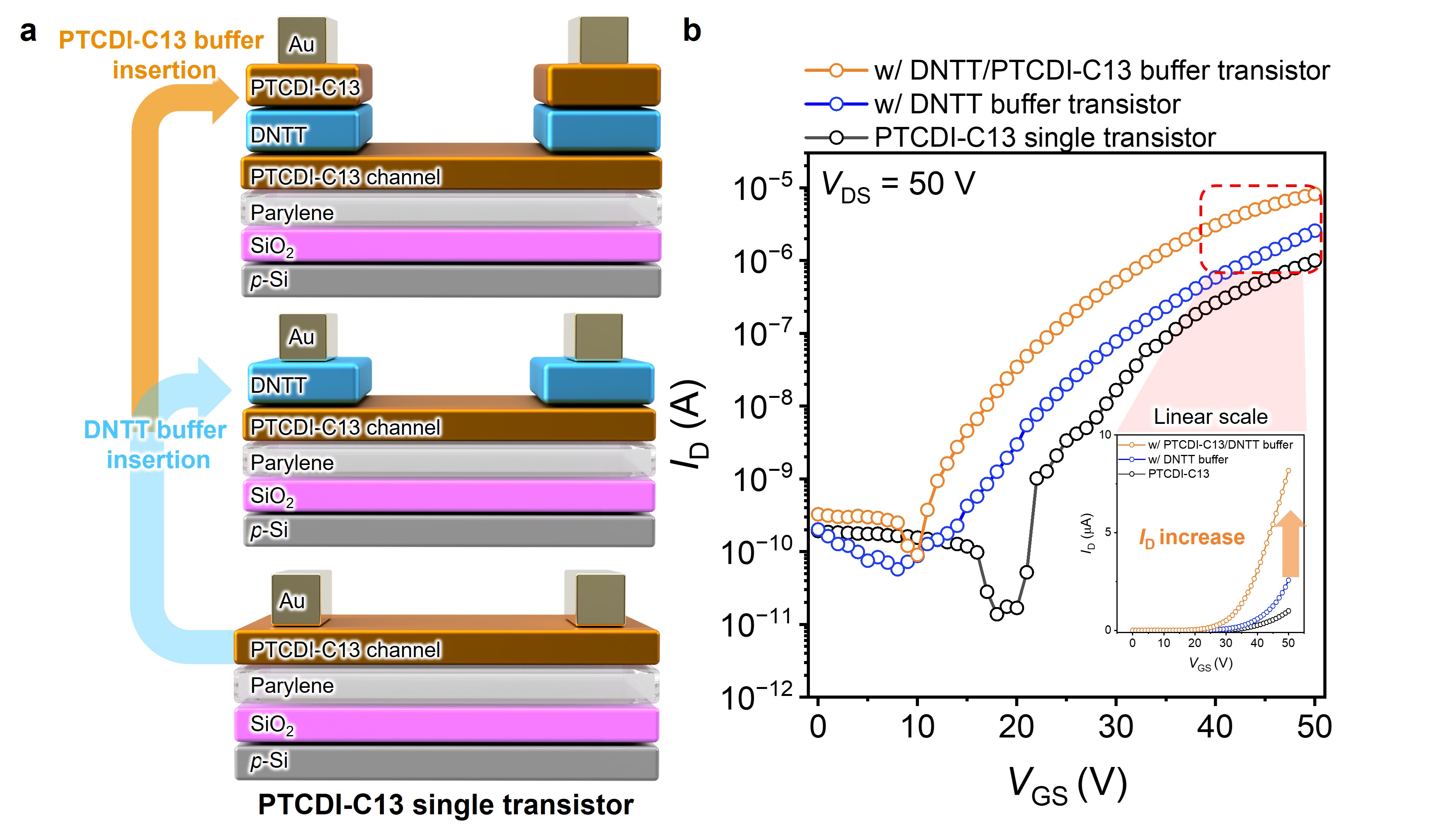
**

**Figure S13.** (a) Device schematics and (b) electrical characteristics of PTCDI-C13 channel-based single and buffered transistors (DNTT and DNTT/PTCDI-C13 hybrid buffers) for validation of the effect of the asymmetric PTCDI-C13 electron injection buffer layer.


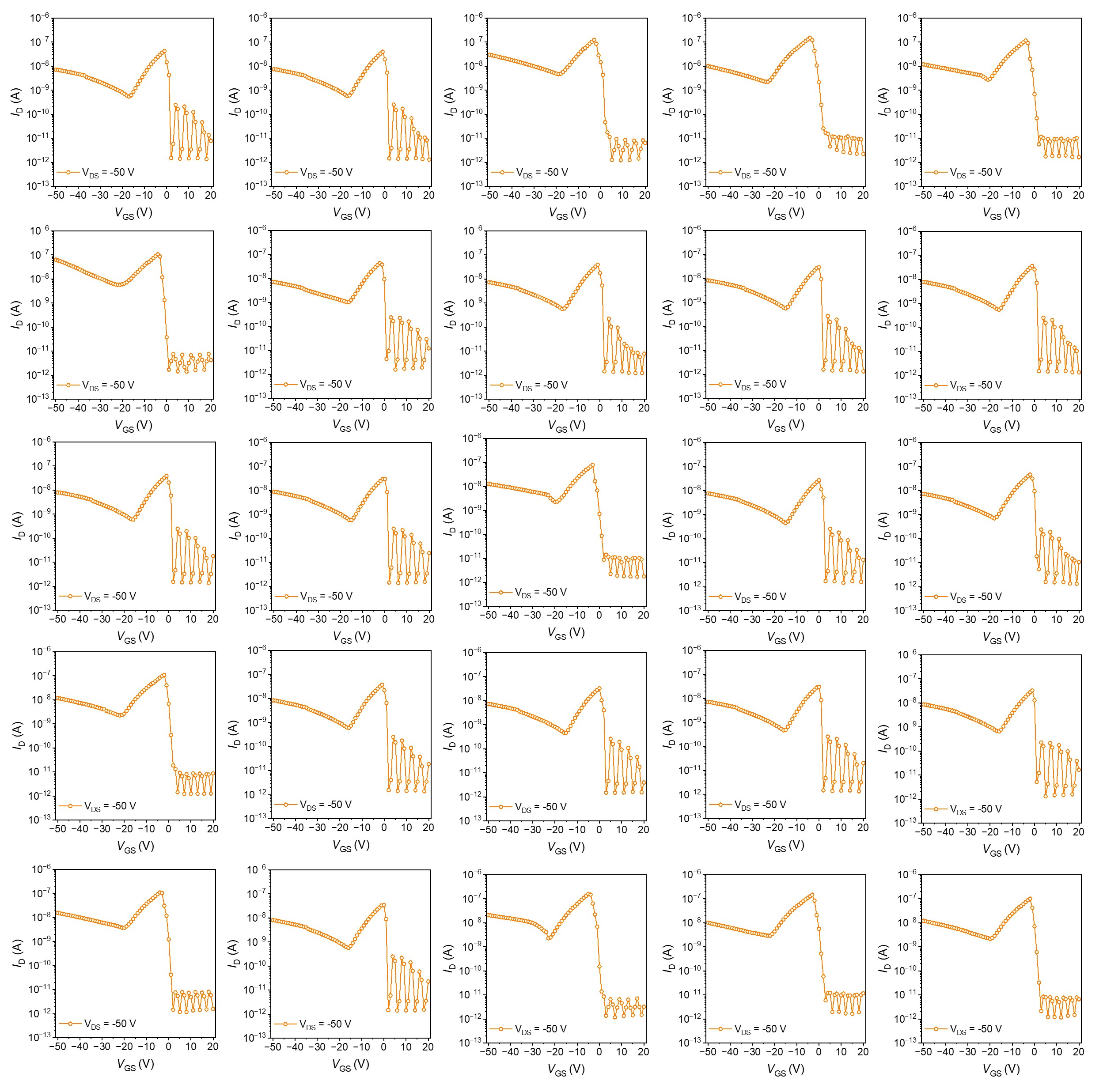


**Figure S14.** 100 transfer curves of NTC transistors to evaluate the device uniformity, and we extract *g*_m_ and NTC region.


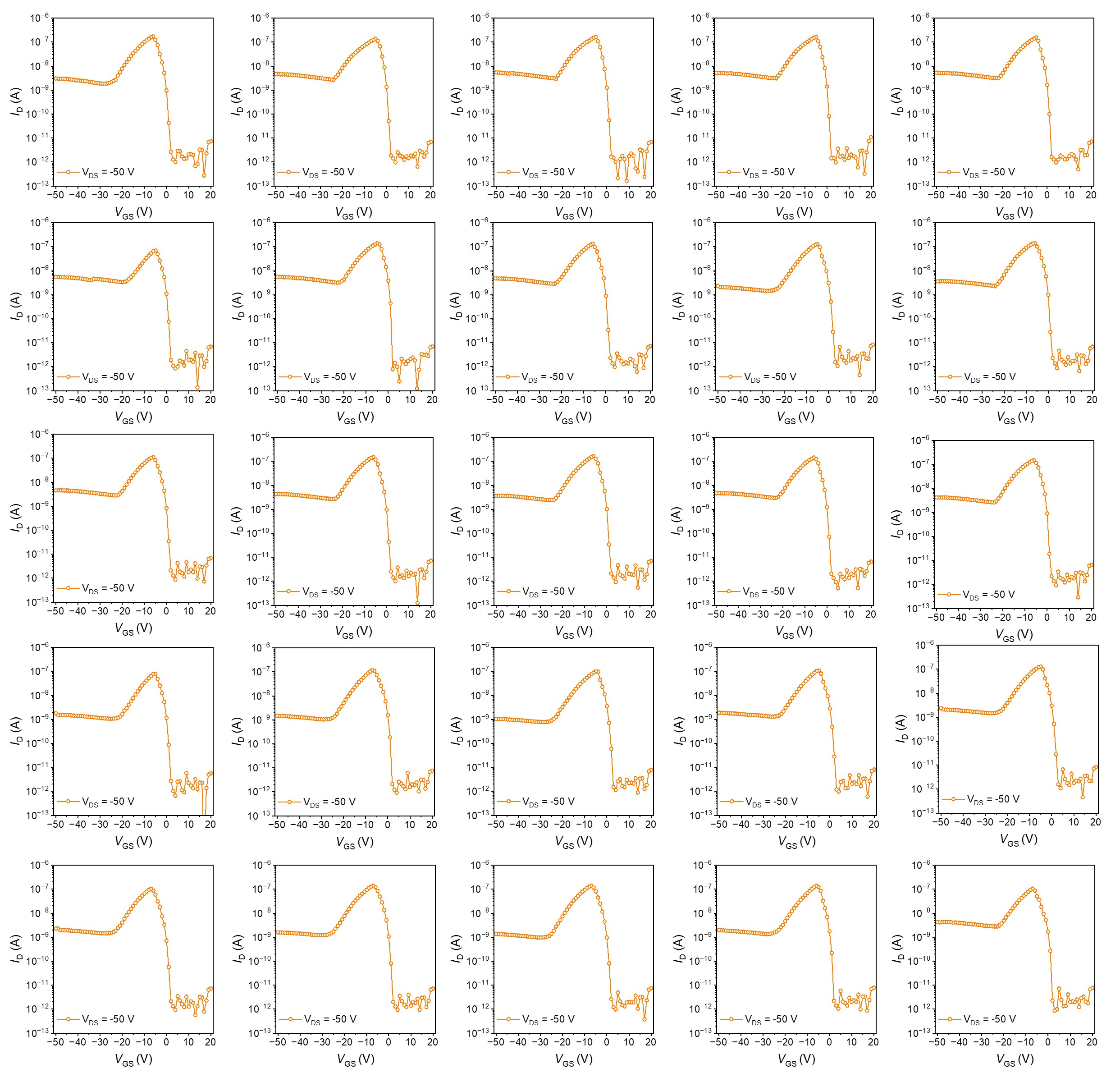

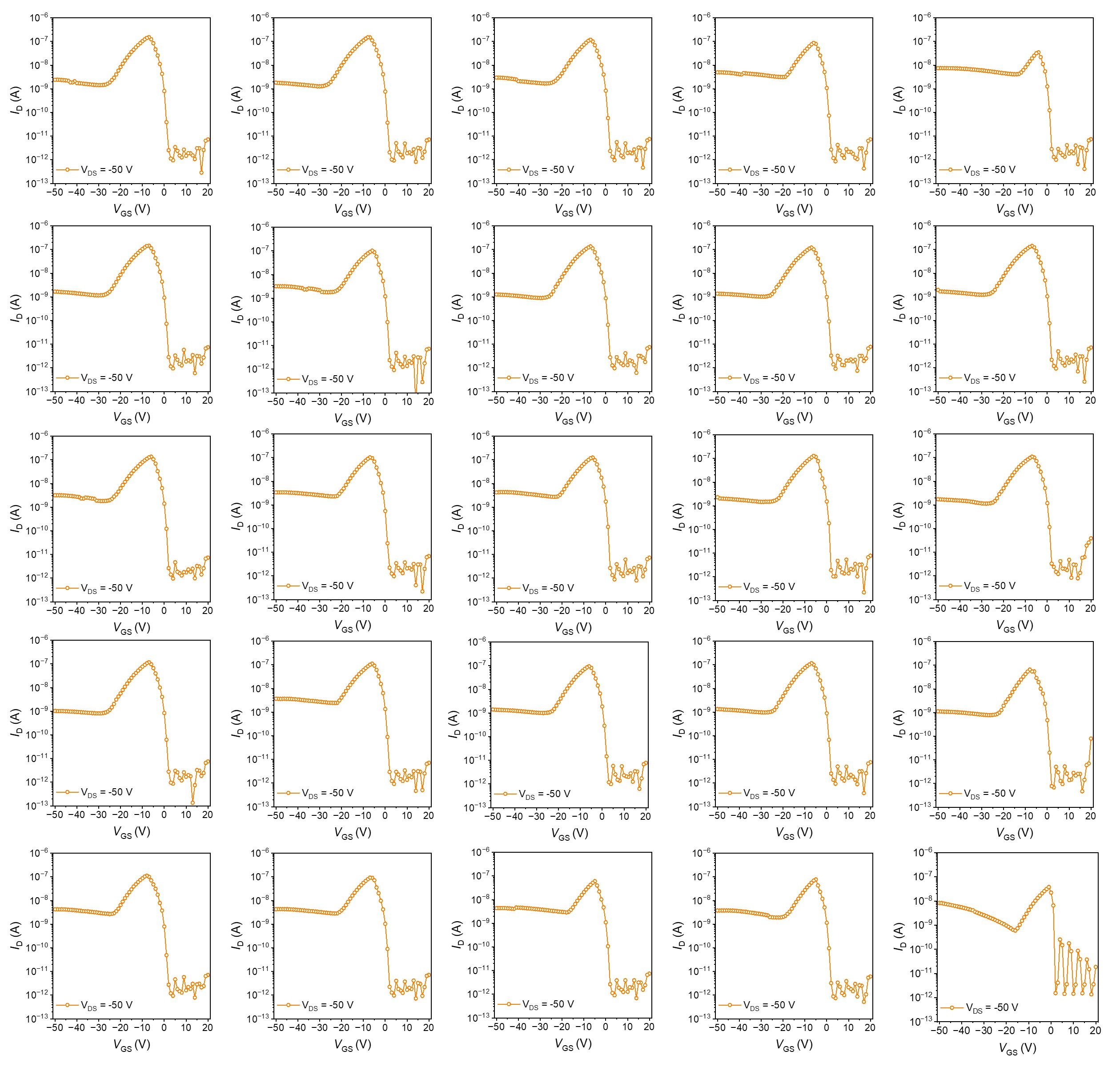


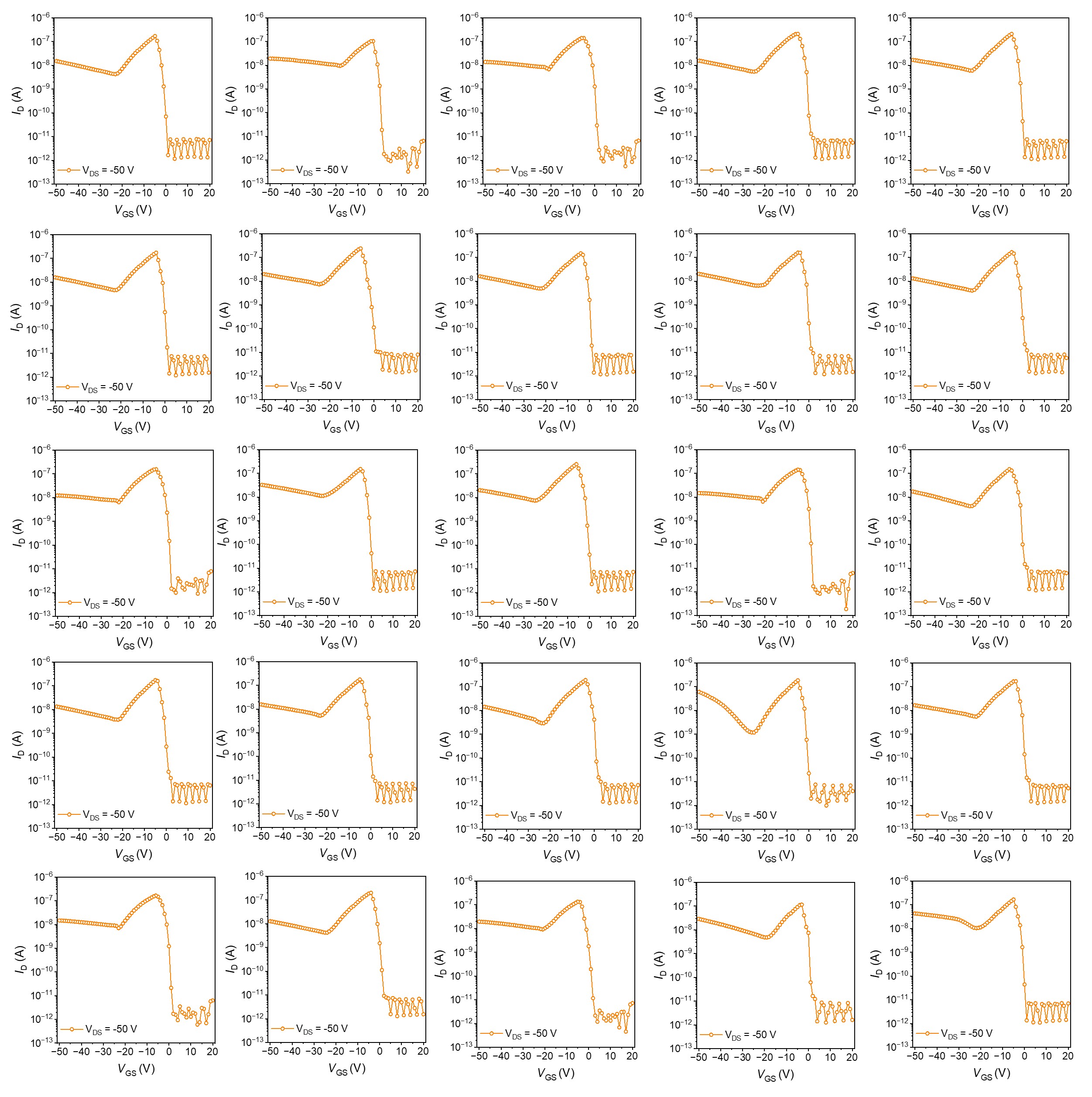


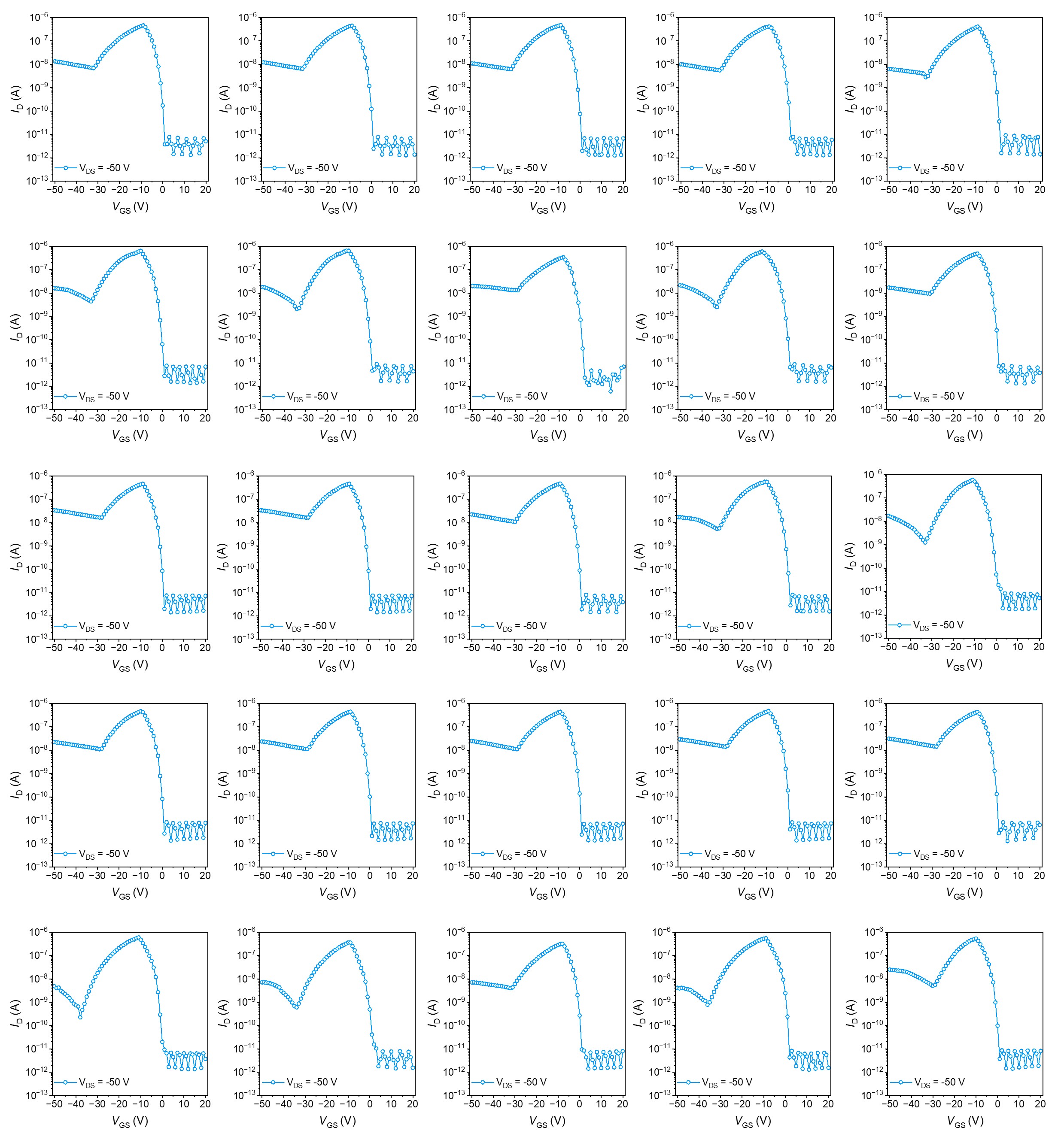


**Figure S15.** 100 transfer curves of BHN-NTC transistors to evaluate the device uniformity, and we extract *g*_m_ and NTC region.


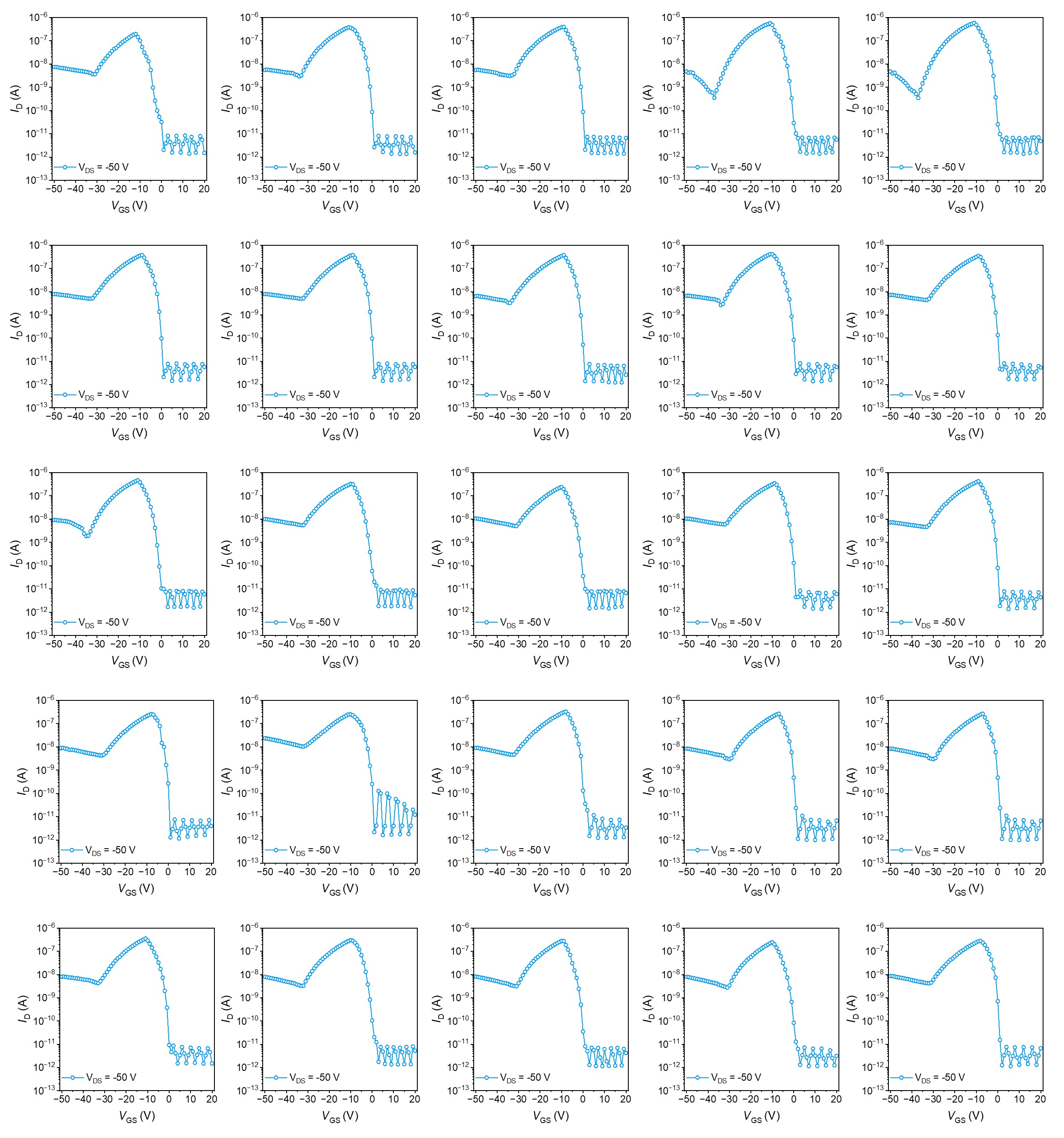

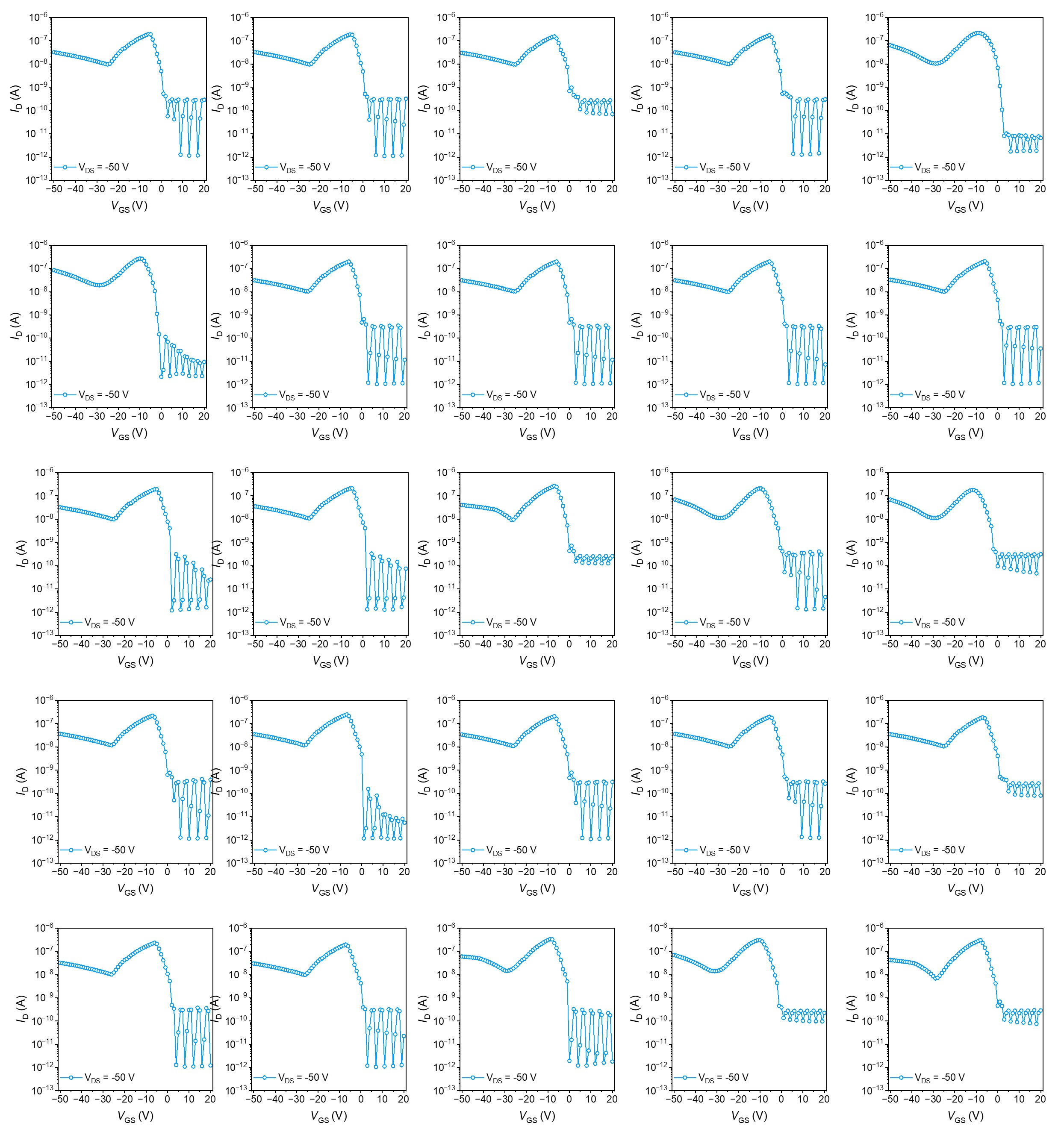


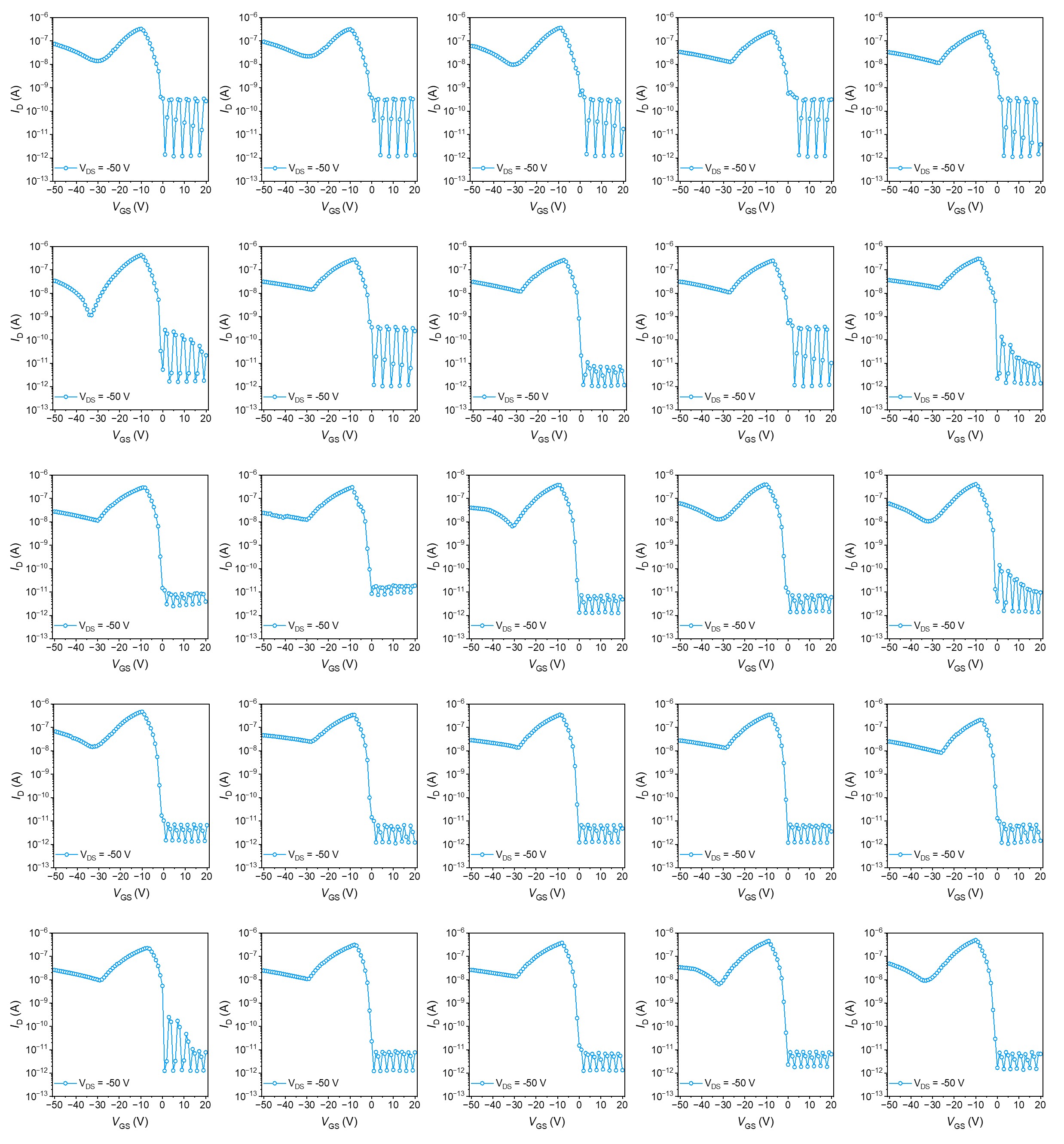


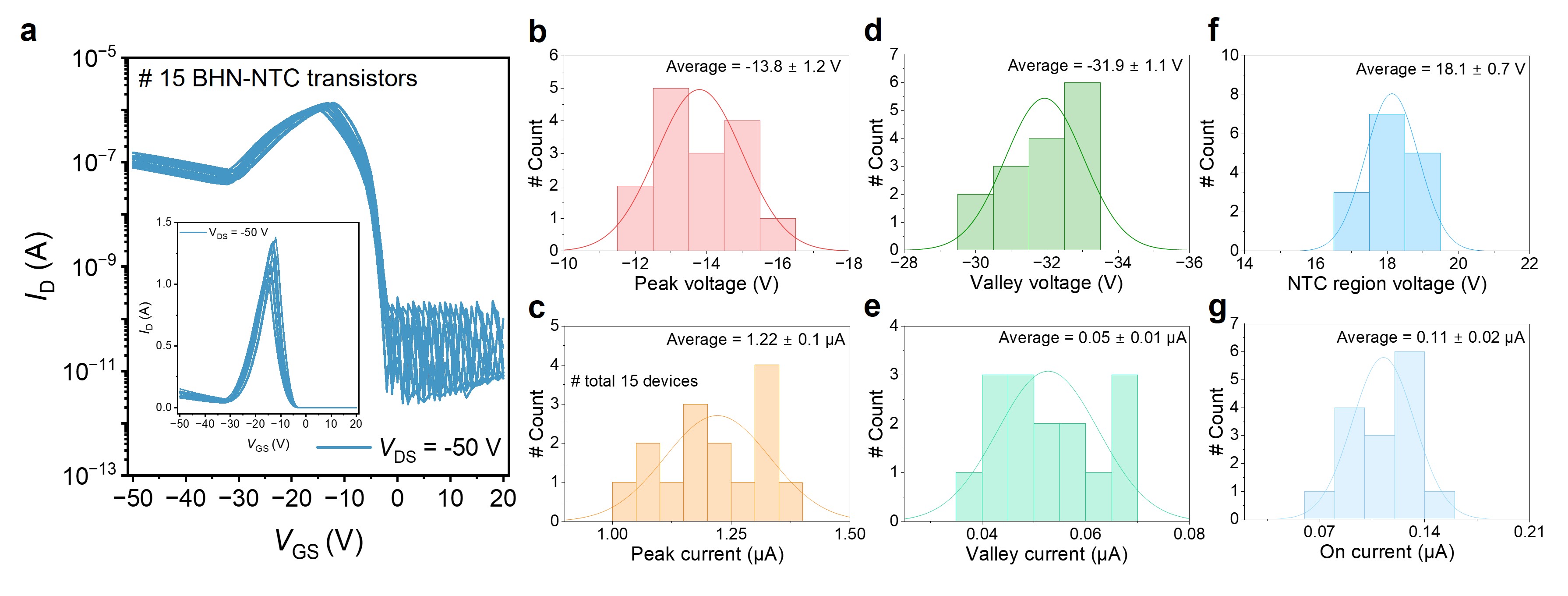


**Figure S16.** (a) 15 transfer curves of CYTOP interfacial trap prevention layer based BHN-NTC transistors to investigate the device uniformity, and we extract (b) peak voltage (c) peak current (d) valley voltage (e) valley current (f) NTC region voltage (g) on current.

**
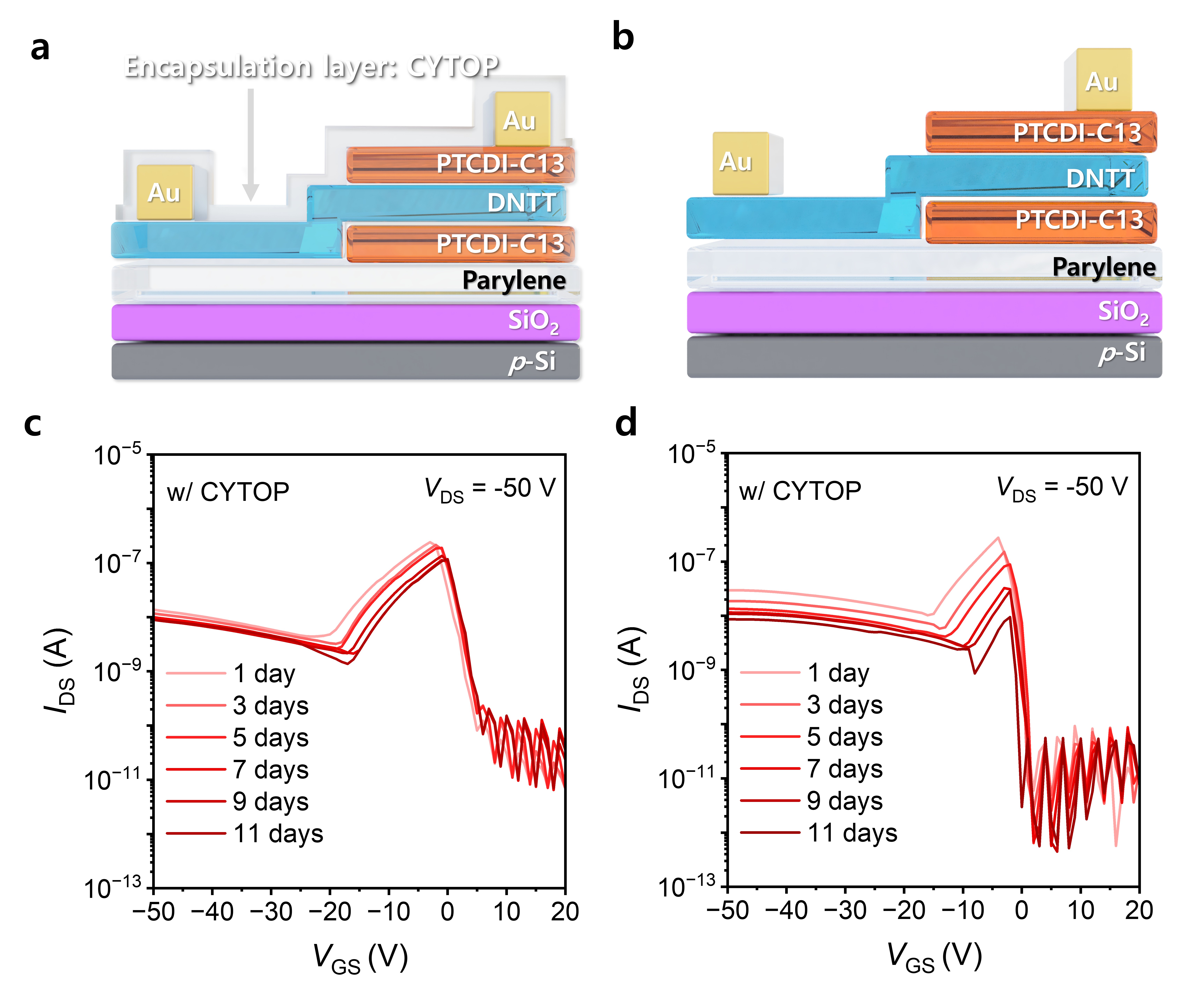
**

**Figure S17.** The comparative analysis of BHN-NTC transistors with and without a CYTOP encapsulation layer was conducted. Schematic diagram of the BHN-NTC transistor (a) with a CYTOP (b) without CYTOP encapsulation layer uniformly covering the electrodes and semiconductor channel. Transfer curves of the BHN-NTC transistor (c) with a CYTOP (d) without CYTOP encapsulation layer measured at two-day intervals over a 11-day period.


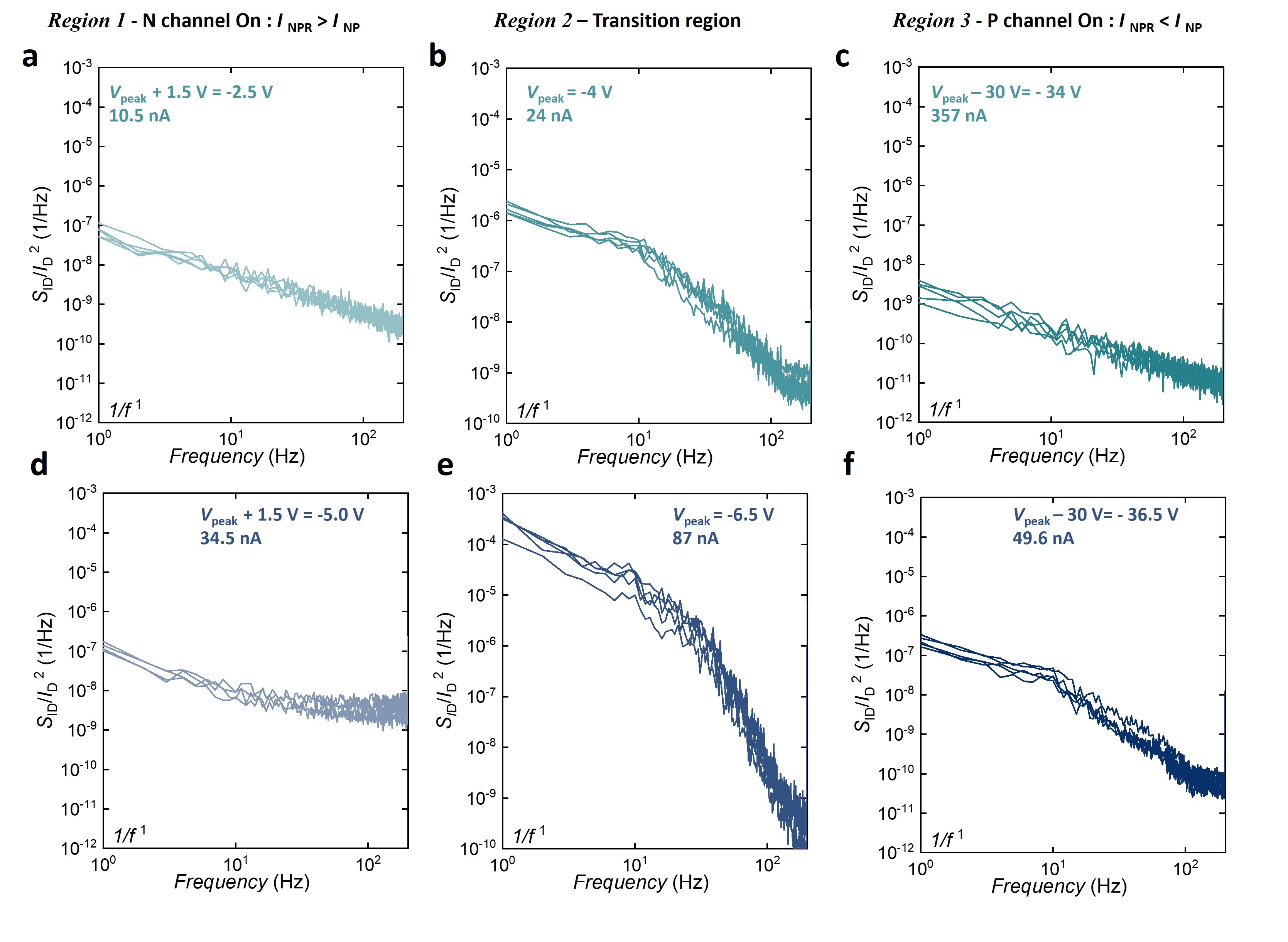


**Figure S18.** Measured *S*_ID_/*I*_D_^2^ versus frequency graphs for NTC and BHN-NTC devices across different operating regions, collected from five distinct devices. (a) NTC, *Region I*, (b) NTC, *Region II*, (c) NTC, *Region III*, (d) BHN-NTC, *Region I*, BHN-NTC, *Region II*, (f) BHN-NTC, *Region III*


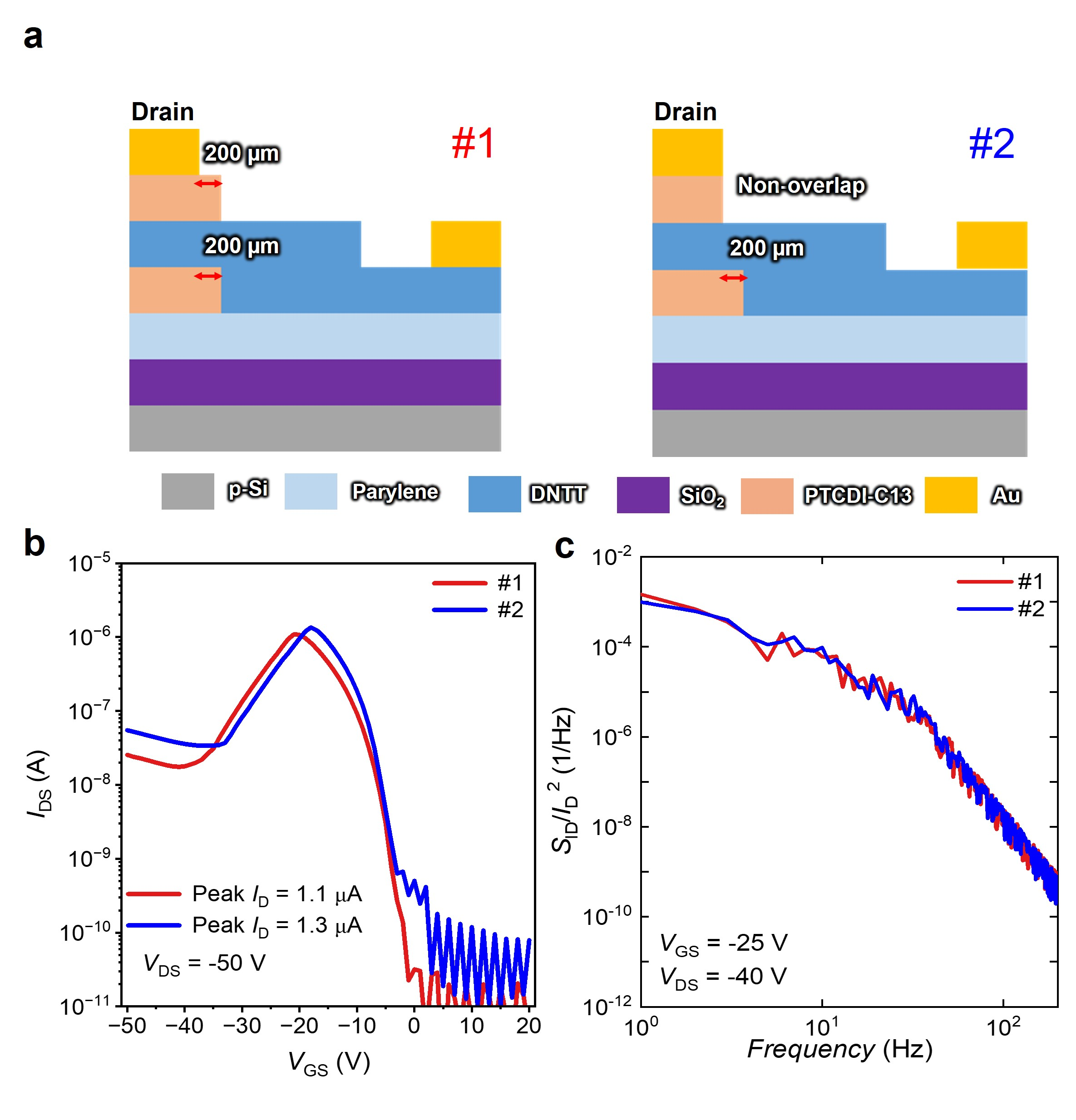
\

**Figure S19.** (a) Schematic cross-sections illustrating two device configurations (#1 and #2) with a fixed bottom PTCDI-C13 layer length of 200 μm, comparing overlapped (#1) versus non-overlapped (#2) top PTCDI-C13 layer geometries. (b) Transfer curves (*I*_D_-*V*_GS_) of devices #1 and #2. (c) Normalized current noise spectra (*S*_ID_/*I*_D_^2^) of devices #1 and #2.


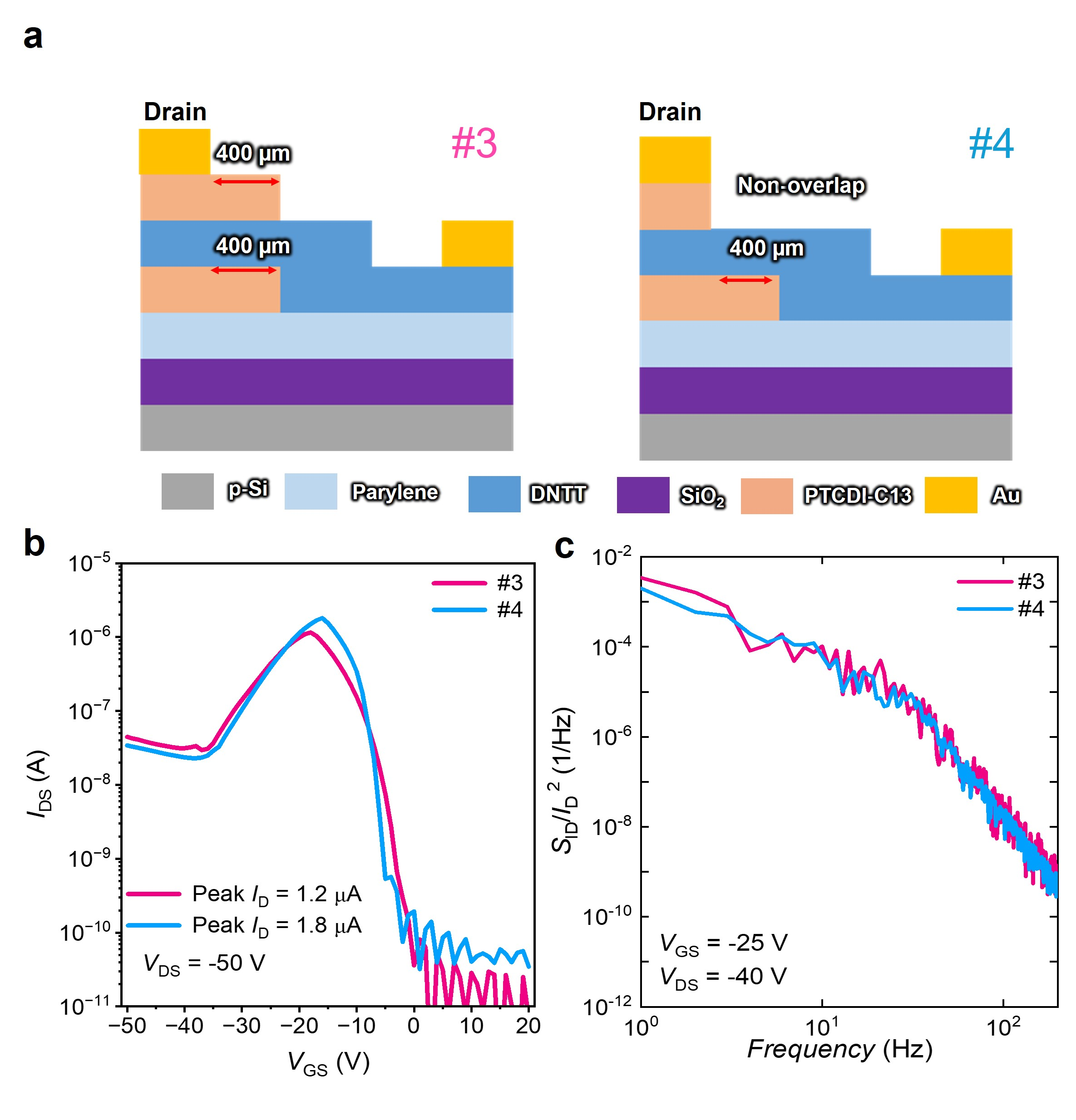


**Figure S20.** (a) Schematic cross-sections of devices (#3 and #4) with a bottom PTCDI-C13 length of 400 μm, comparing overlapped (#3) and non-overlapped (#4) top layers. (b) Transfer curves (*I*_D_-*V*_GS_) of devices #3 and #4. (c) Normalized current noise spectra (*S*_ID_/*I*_D_^2^) of devices #3 and #4.


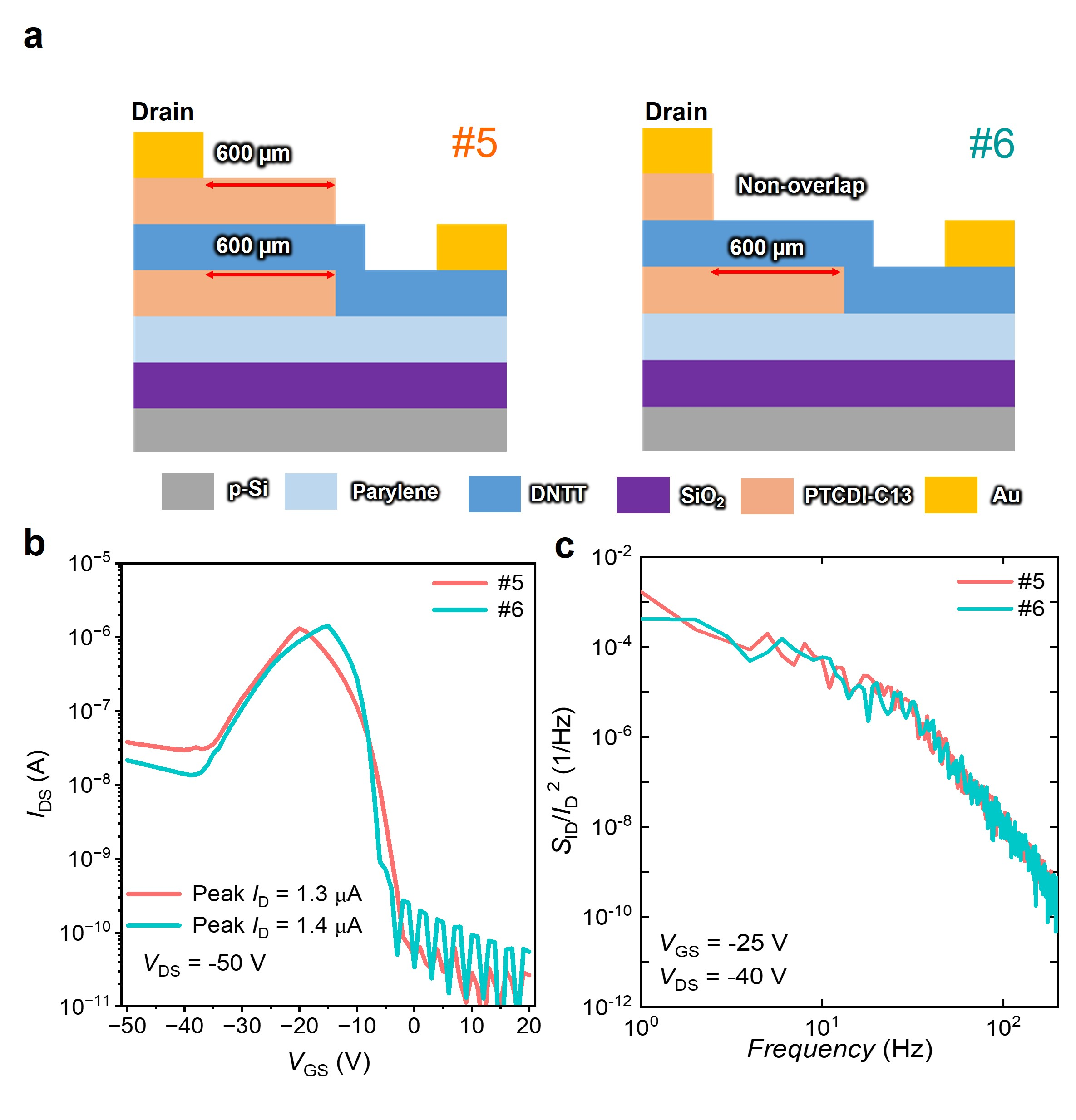


**Figure S21.** (a) Device schematics for a larger bottom PTCDI-C13 length of 600 μm, comparing overlapped (#5) and non-overlapped (#6) configurations. (b) Transfer curves (*I*_D_-*V*_GS_) of devices #5 and #6. (c) Normalized current noise spectra (*S*_ID_/*I*_D_^2^) of devices #5 and #6.


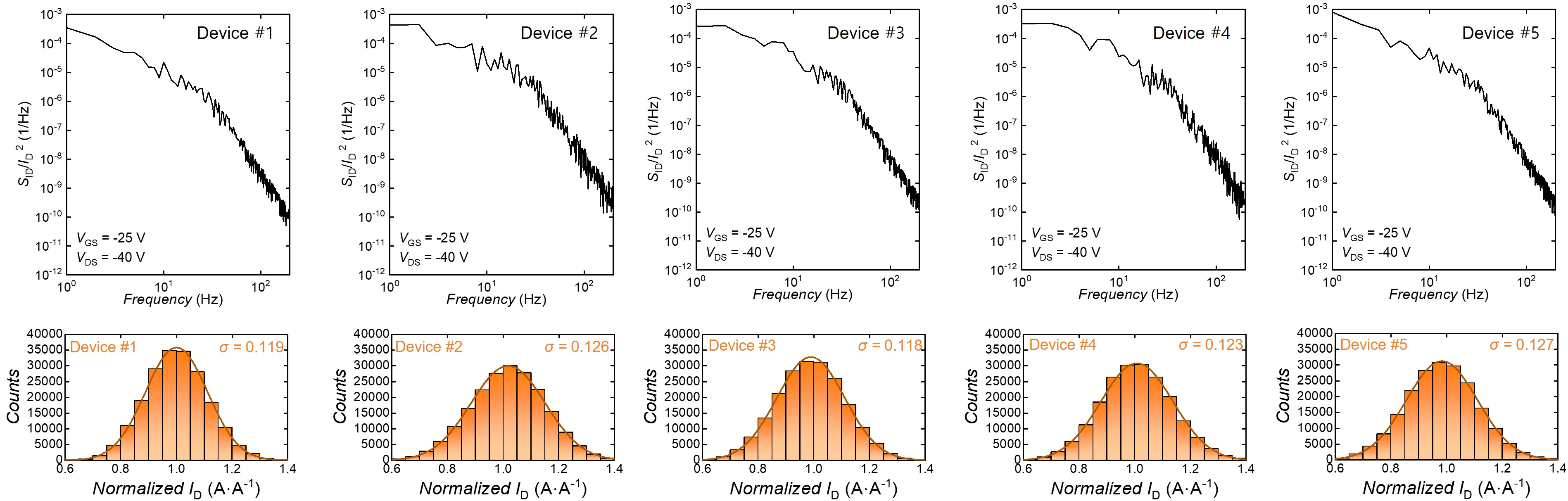


**Figure S22.** Device-to-device consistency of noise characteristics. Top panel: *S*_ID_/*I*_D_^2^ spectra measured in *Region II* for five randomly chosen BHN-NTC transistors fabricated in the same run; despite visible differences in stripe overlap, the curves differ only slightly over the entire frequency range. Bottom panel: histograms of the 500 Hz transient drain-current traces recorded at the same bias for the same five devices; Gaussian fits give σ values of 0.119, 0.126, 0.118, 0.123, and 0.127, demonstrating that the current-fluctuation window varies by roughly ten percent across the set. The similarity in both frequency and time domains confirms that modest edge offsets introduced during fabrication do not materially affect the excess noise as long as the N-P-N junction is present.


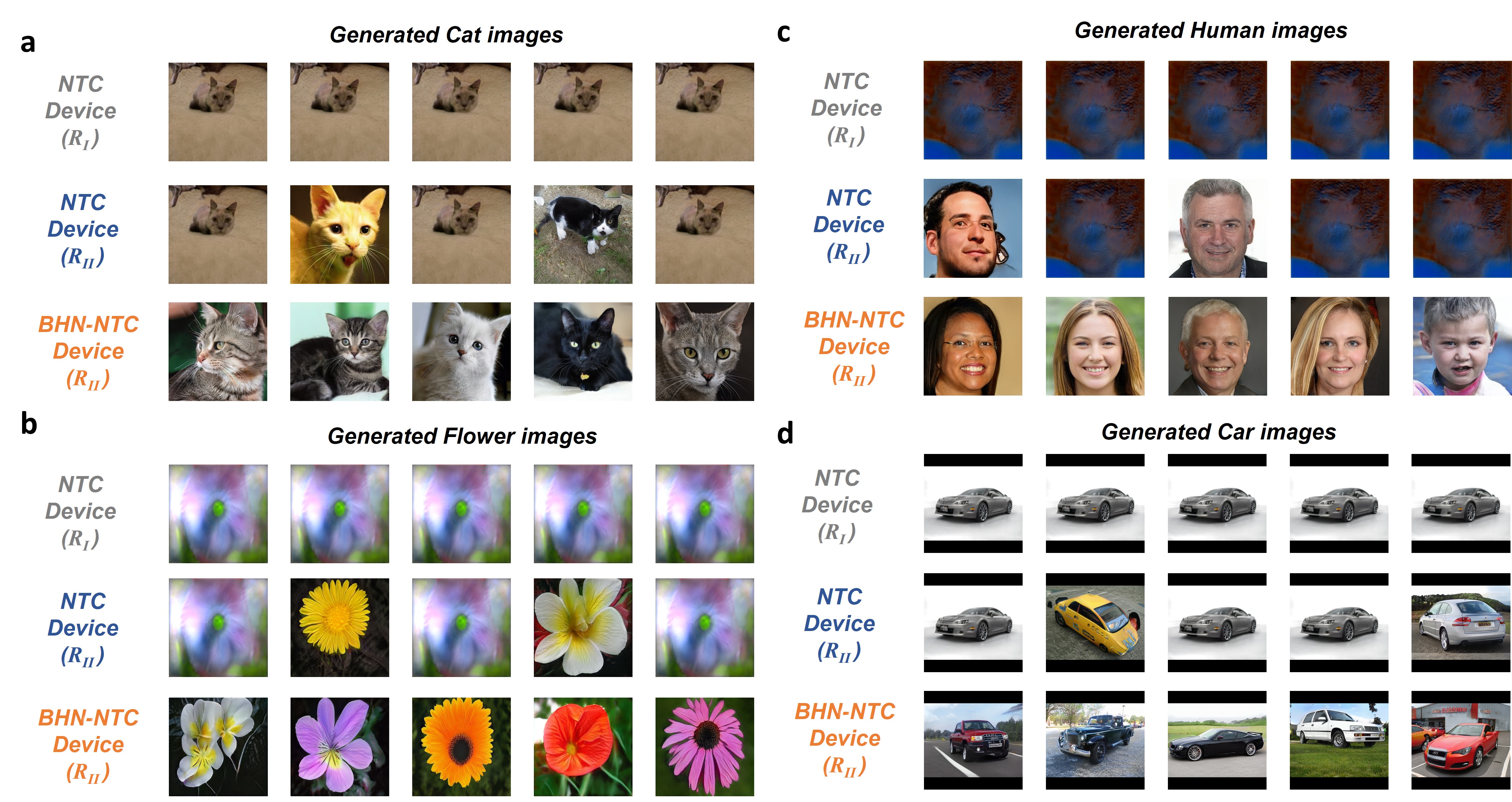


**Figure S23.** Examples of images generated in various domains using the read noise from NTC *Region I*, NTC *Region II*, and BHN-NTC *Region II* as latent vectors. (a) Cat, (b) Flower, (c) Human, (d) Car.


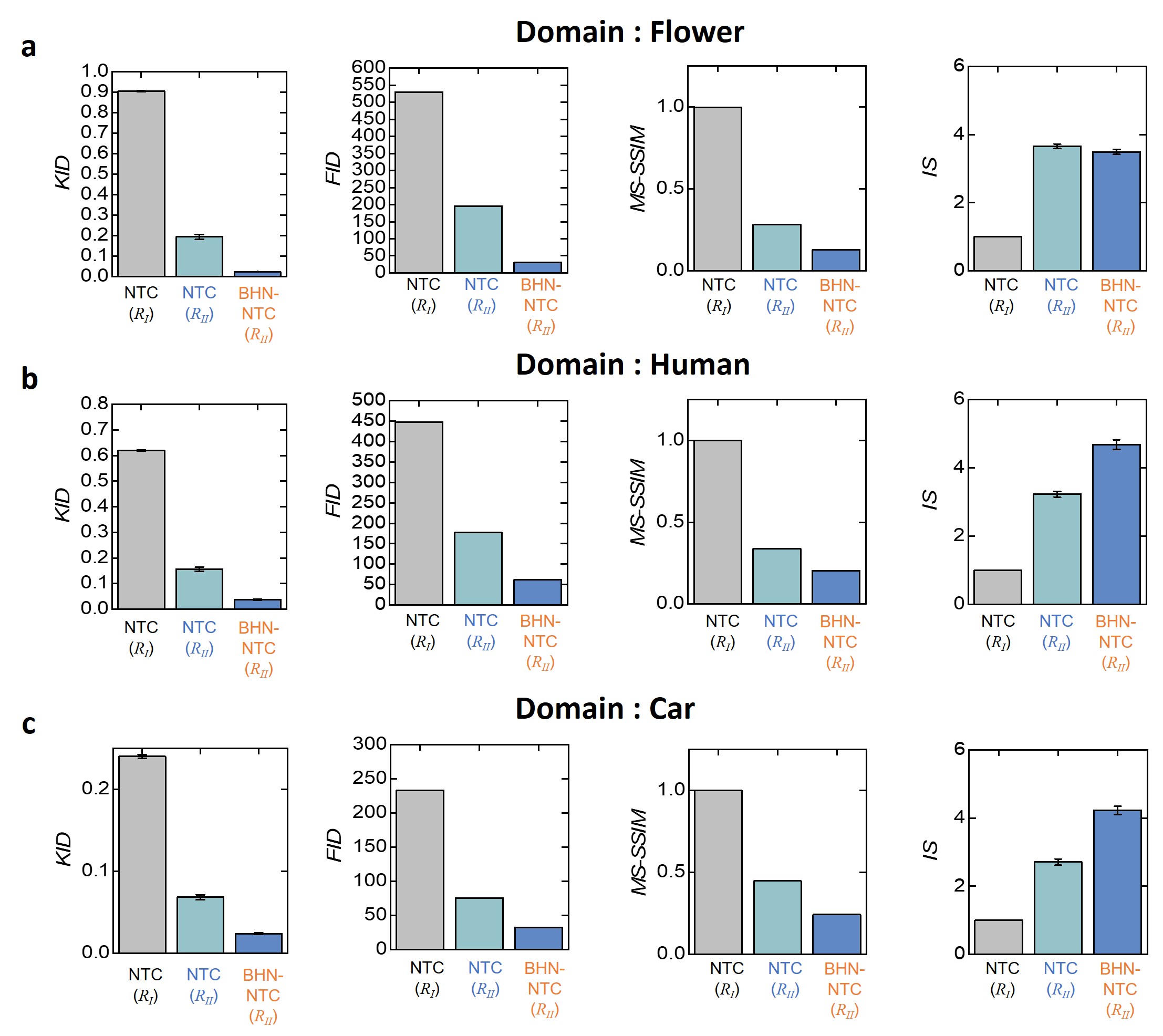


**Figure S24.** Various evaluation metrics (KID, FID, MS-SSIM, IS) for 10,000 images generated in different domains using the read noise from NTC region *I*, NTC *Region II*, and BHN-NTC *Region II* as latent vectors. (a) Flower, (b) Human, (c) Car.


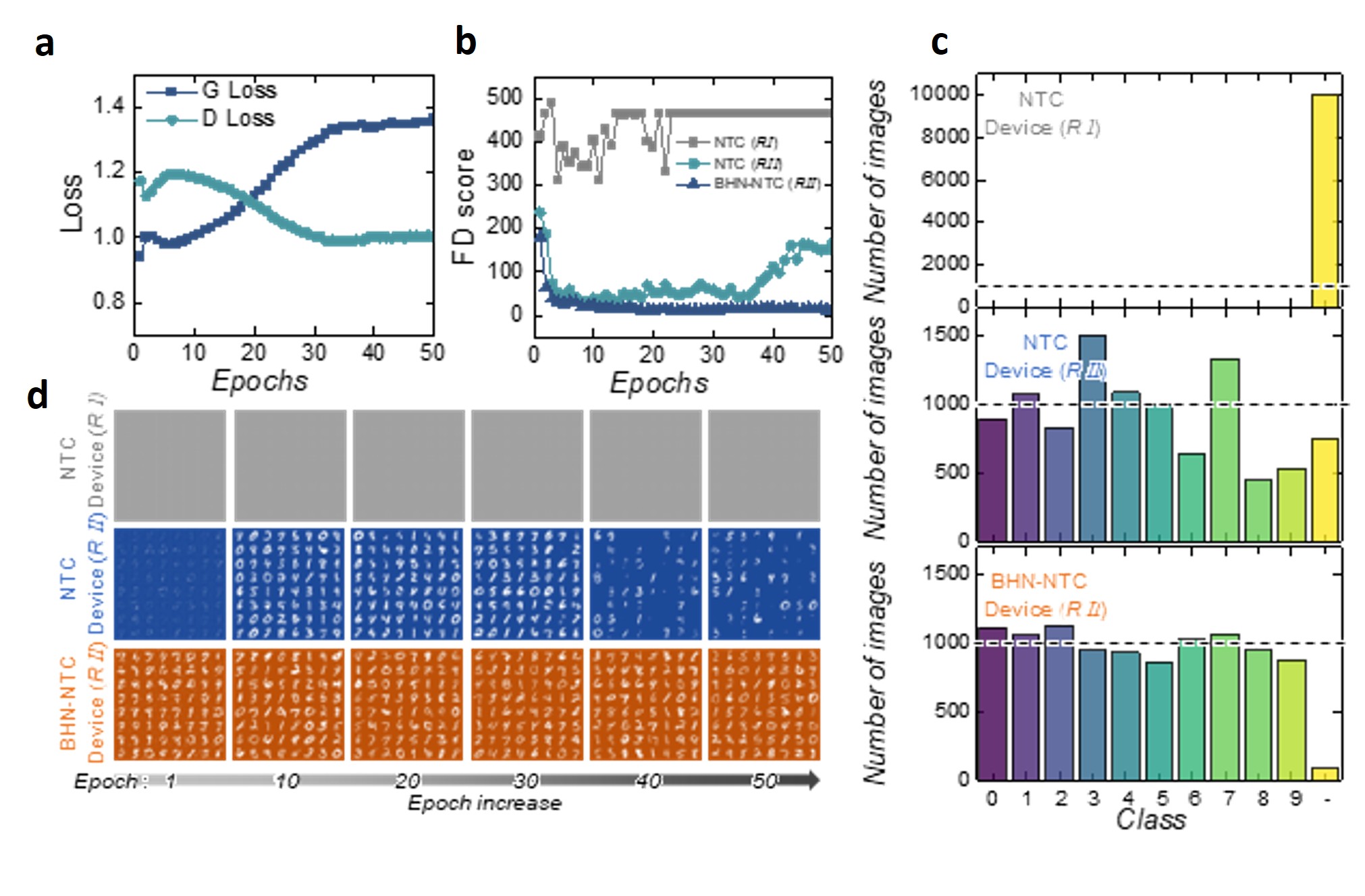


**Figure S25.** (a) G model and D model losses over training epochs. (b) FD scores as a function of the epoch for NTC(*R_I_*), NTC(*R_II_*), and BHN-NTC(*R_II_*). (c) Classification results of the generated images.(d) Progression of the generated images over training epochs for each device.


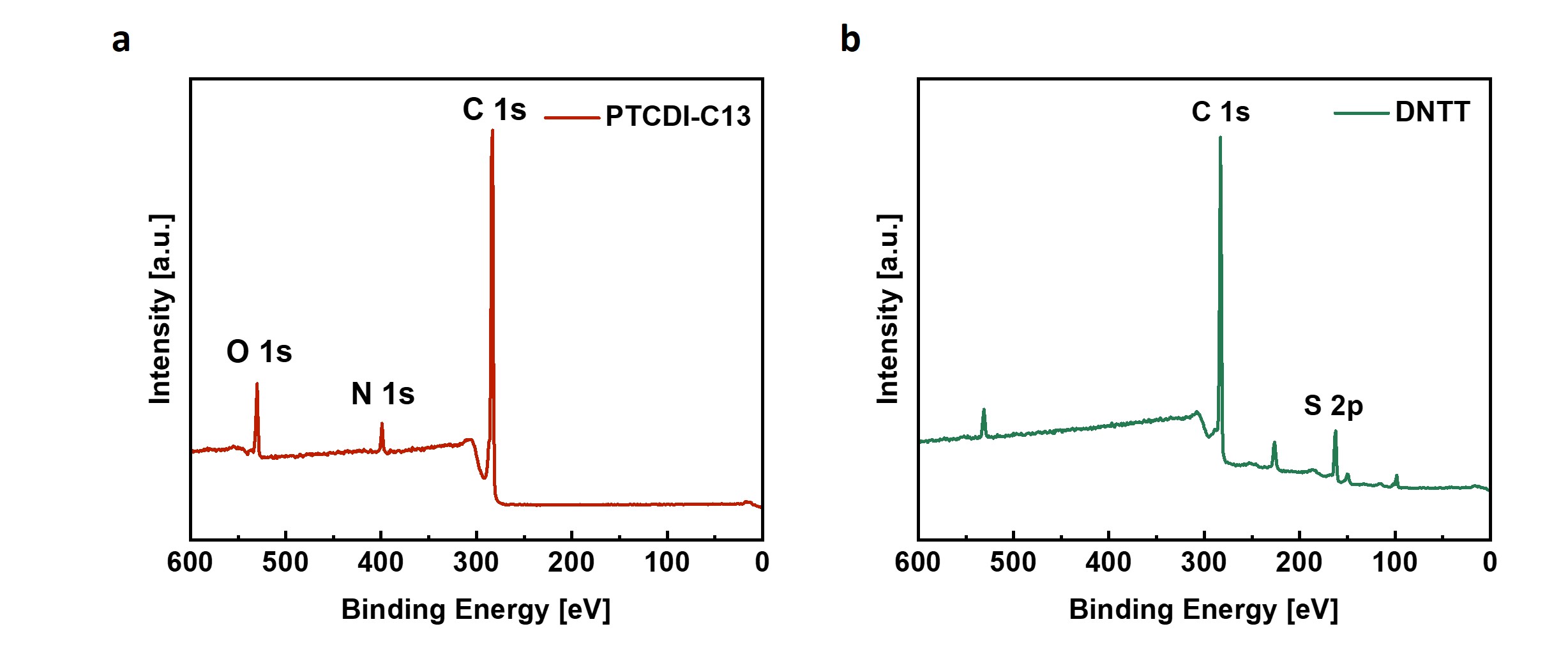


**Figure S26.** XPS analysis of (a) PTCDI-C13 (b) DNTT.

| Device schematic | Pull-up  transistor | Pull-down  transistor | Peak  current | NTC region | Middle logic length | Middle logic efficiency | Year | [ref] |
| --- | --- | --- | --- | --- | --- | --- | --- | --- |
| 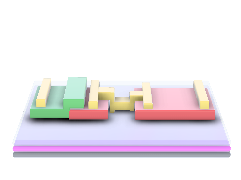 | PTCDI-C8/  α-6T | PTCDI-C8 | ≈ 0.1 μA | 2.9 V | 1.5 V | 37.5 % | 2018 | [S1] |
| 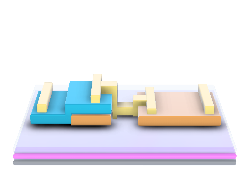 | DNTT/  PTCDI-C13 | PTCDI-C13 | 1.35 μA | 4 V | 4 V | 8 % | 2019 | [S2] |
| 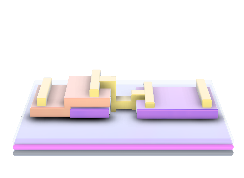 | C8-BTBT /PhC_2_-BQQDI | PhC2-BQQDI | 1.5 μA | 16 V | 17 V | 28.3 % | 2022 | [S3] |
| 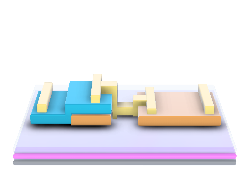 | DNTT/  PTCDI-C13 | PTCDI-C13 | 20 nA | 1.7 V | 2.2 V | 37 % | 2023 | [S4] |
| 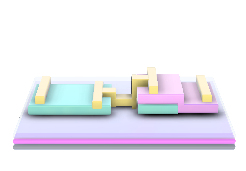 | Cu_2_O | IGZO/  Cu_2_O | ≈ 1 μA | 29 V | 7 V | 23 % | 2024 | [S5] |
| 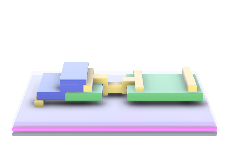 | MoS_2_/  SWCNT | MoS_2_ | ≈ 0.1 μA | ≈ 0.6 V | ≈ 0.5 V | 16.7 % | 2025 | [S6] |
| 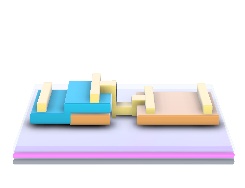 | PTCDI-C13/  DNTT | PTCDI-C13 | 0.35 μA | 19 V | 17 V | 34 % | This work |  |
| 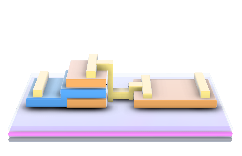 | **PTCDI-C13/**  **DNTT/**  **PTCDI-C13** | **PTC`DI-C13** | **1.11 μA** | **27 V** | **27.5 V** | **55 %** | **This work** |  |

**Table S1.** Comparison table of NTC-based transistors and their corresponding implementation results in multi-valued logic (MVL) applications.

| Device/Platform | External amplifier | Bits per sample | Key advantages | Main limitations | Image generation demonstration | [ref] |
| --- | --- | --- | --- | --- | --- | --- |
| Diffusive Ag:SiO₂ memristor TRNG | Sense resistor + comparator chain | 1 bit | -Bias free entropy,  - Simple  device stack | Extra analogue chain;  Single bit throughput | No | [S7] |
| h-BN RTN memristor TRNG | Low noise amplifier | 1 bit | -Low voltage  - Low power | Needs off chip gain; single-bit output | No | [S8] |
| Cryptoristor  (floating body FinFET) | On‑board noise‑coupled SAR‑ADC (8‑bit, includes SAR logic & FIA) | 2 bits (2LSB) | - CMOS compatible | Still limited to one bit per cycle | No | [S9] |
| **BHN NTC transistor  (This work)** | **None**  **(Direct flash-ADC)** | **3 bits** | **- Intrinsic noise amplification**  **- Multi bit entropy**  **- Improves StyleGAN2 (FID ↓ 18.7→8.3)** | **High voltage** | **Yes – StyleGAN2** | **This work** |

**Table S2.** Benchmark of representative hardware TRNGs for contextualizing the present work and comparing device performance with prior studies.

**Note S1:** Analysis of the anti-ambipolar transistor conducted to understand the operating characteristics of the NTC and BHN-NTC transistors.

We fabricated a separate AAT device and measured the electrical characteristics of a *p*-type based AAT structure (Figure S1, Supporting Information). To clarify the operation mechanism of the AAT device composed of DNTT and PTCDI-C13, the gate voltage range can be divided into three distinct regions based on the current flow behavior under a fixed drain-to-source voltage (*V*_DS_ = −50 V):

(i) First, in the range of −5 V < *V*_GS_ < 20 V, DNTT remains in the off-state due to the insufficient gate bias to induce hole accumulation. Although the gate-to-drain voltage (*V*_GD_ = *V*_G_ − *V*_D_) in this region ranges from 70 V to 45 V—sufficient to turn on the *n*-type PTCDI-C13 channel—current cannot flow through the AAT structure because the two semiconductors are connected in series. Since DNTT is not conducting, the entire channel path is depleted.

(ii) Second, in the region of −35 V < *V*_GS_ < −5 V, DNTT gradually turns on as the *V*_GS_ becomes more negative, leading to increased current flow. The current reaches a peak at *V*_GS_ = −15 V, which we define as the peak voltage. The peak voltage corresponds to the condition where the combined resistance of DNTT and PTCDI-C13 is minimized, and the resulting current, defined as the peak current, was measured to be 0.2 μA. As *V*_GS_ is swept more negatively from −15 V to −35 V, hole accumulation in the *p*-type DNTT is enhanced, promoting its channel conductivity. However, as *V*_GS_ is swept in the negative direction, *V*_GD_ decreases from 35 V to 15 V, reducing the gate-to-drain electric field in PTCDI-C13 and resulting in a gradual decrease in the overall current.

(iii) Third, in the region of −50 V < *V*_GS_ < −35 V, DNTT remains turned on, allowing for substantial hole transport. However, *V*_GD_ continues to decrease from 15 V to 0 V, which severely limits the ability of PTCDI-C13 to conduct electrons. As a result, the current flow through the AAT structure is once again depleted due to the lack of conduction in the PTCDI-C13 channel. These observations collectively result in a distinct ∧-shaped current–voltage characteristic, which is a hallmark of anti-ambipolar behavior in the AAT structure.

**Note S2:** Analysis of energy barrier differences based on Arrhenius plot analysis.

To provide evidence supporting the mechanism that the reduced energy injection barrier in the BHN-NTC transistor enables more efficient charge injection, we performed a detailed Arrhenius analysis based on the peak current measured at various temperatures (293.15 K, 313.15 K, 333.15 K, 353.15 K, 373.15 K). The Arrhenius equation used for this analysis is as follows ^[S10]^:

$$ln(\frac{I_{DS}}{T^{\frac{3}{2}}})= -\frac{q\Phi_{B}}{k_{B}T}+\ln A^{*}$$

*I*_DS_^​^ is the drain current measured at a given temperature, *T* is the absolute temperature, *q* is the elementary charge, Φ_B_​ is the Schottky barrier height, *k*_B_​ is the Boltzmann constant, A^*^ is the effective Richardson constant. As a result, the Arrhenius plots were constructed using drain currents measured at various gate voltages, and the injection barriers were extracted from the slopes of the fitted linear curves. The NTC transistor exhibited consistently higher injection barriers of 0.25–0.28 eV (Figure S6a), while the BHN-NTC transistor showed significantly lower values ranging from 0.11 eV to 0.19 eV (Figure S6b). These results demonstrate that, across the NTC region, the BHN-NTC transistor maintains lower electron injection barriers compared to the conventional NTC transistor.

**Note S3:** Investigation of the extended NTC region and implications for ternary logic applications.

Due to the enhanced electron injection, the BHN-NTC transistor achieved a wider NTC region compared to conventional *pn* junction-based NTC transistors. We intended to demonstrate that the extended NTC region of the BHN-NTC transistor, when implemented for MVL, enables an expanded middle logic state, thereby improving MVL state margin. Therefore, we implemented MVL by using NTC and BHN-NTC transistors for the pull-up transistor and a PTCDI-C13 transistor for the pull-down transistor (Figure S9). Based on the voltage transfer curve (VTC) of the complementary ternary circuit, we confirmed that the middle logic length of the BHN-NTC transistor is extended to 27.5 V, which is approximately 10 V wider than that of the conventional NTC transistor (Figure S10). Originating from enhanced charge injection enabled by the asymmetrical PTCDI-C13 structure, this improvement leads to increased *g*_m_ and peak-to-valley current ratio, thereby expanding the NTC region and extending the middle logic state range for ternary logic applications. To further support our findings and provide a quantitative comparison with previous studies, we extracted and evaluated key parameters such as the NTC region, middle logic length, and middle logic efficiency (defined as the middle logic length/maximum input voltage), (Table S1). These results demonstrate that the enhanced electron injection efficiency due to the asymmetric PTCDI-C13 layer contributes to the expansion of the NTC region, thereby improving the overall performance of the BHN-NTC transistor.

**Note S4:** Contact angle measurement for influence of additional PTCDI-C13 layer using OWRK model.

We conducted contact angle measurements using deionized water (DI-water) and formamide as probe liquids on single layer PTCDI-C13 and DNTT, as well as NTC and BHN-NTC heterostructure. The surface energy (γ_S_) was calculated through the contact angle analysis method using the Owens–Wendt–Rabel–Kaelble (OWRK) model, which decomposes γ_S_ into its dispersive ($\gamma_{S}^{d}$) and polar ($\gamma_{S}^{p}$) components. The model is expressed by the following equation ^[S11, S12]^:​

${\text{ }\text{γ}}_{\text{L}}\text{(1+}\cos\text{θ}\text{)=2(}\sqrt{\text{γ}_{\text{S}}^{\text{d}}\text{∙}\text{γ}_{\text{L}}^{\text{d}}}\text{+}\sqrt{\text{γ}_{\text{S}}^{\text{p}}\text{∙}\text{γ}_{\text{L}}^{\text{p}}}$)

Where, γ_L_ represents the total surface tension of the liquid, $\gamma_{L}^{d}$ and $\gamma_{L}^{p}$ are its dispersive and polar components, respectively, and θ is the measured contact angle on the solid surface. Surface energy measurements (Figure S12) revealed that stacking DNTT and PTCDI-C13 layers progressively reduces surface energy, from 34.5 mN·m⁻¹ (PTCDI-C13) and 28.0 mN·m⁻¹ (DNTT) to 19.3 mN·m⁻¹ and finally 17.1 mN·m⁻¹ in the PTCDI-C13/DNTT/PTCDI-C13 structure. This reduction is attributed to increased surface roughness, which introduces air gaps during contact angle measurements and results in lower apparent surface energy^[S13]^. Although such roughness typically degrades device performance due to interfacial disorder and trap states, the BHN-NTC transistor exhibited improved electrical characteristics and an increase in low-frequency noise. These effects are explained by (1) an expanded electron injection area due to rough interfaces and (2) more favorable energy level alignment provided by the PTCDI-C13 buffer. As a result, despite the increased density of trap states introduced by the roughened interfaces, the enhanced carrier injection enables a greater portion of carriers to successfully reach the channel, thereby improving the overall electrical performance. Concurrently, the elevated trap density promotes more frequent trapping and detrapping events, which in turn amplifies the low-frequency noise characteristics.

**Note S5:** Increased surface roughness–induced expansion of contact area, improved charge injection efficiency, and reduced contact resistance.

The observed reduction in contact resistance is attributed to the complementary effect of two mechanisms: improved electron injection enabled by the asymmetric PTCDI-C13 layer and an expanded charge injection area resulting from increased surface roughness. To clarify the role of surface roughness in enhancing electron injections in the BHN-NTC structure, we fabricated three types of PTCDI-C13 channel transistors: PTCDI-C13 single-channel transistor, a PTCDI-C13 channel transistor with a DNTT buffer layer, and with a DNTT/PTCDI-C13 bilayer buffer (Figure S13). Through the comparison of measured electrical characteristics, it was confirmed that, despite DNTT being a *p*-type semiconductor with intrinsically limited electron transport, its incorporation as a buffer layer enhanced device performance. This result indicated that increased interfacial roughness expanded the injection area and enabled more efficient electron injection. Additionally, the addition of a DNTT/PTCDI-C13 bilayer buffer further enhanced on-current and brought the turn-on voltage closer to 0 V. These findings provided experimental evidence for the complementary mechanisms of injection area expansion and enhanced electron injection via reduced energy barriers.

**Note S6:** Length-dependence of low-frequency noise in BHN-NTC transistors.

To determine whether the magnitude of the LFN in a BHN-NTC transistor depends on the physical width of the heterojunction, we fabricated six additional devices that differed only in the lateral length of their PTCDI-C13 stripes. In every case, the process flow, layer thicknesses, and contact geometry were identical to those described in the main text; only the overlap area was adjusted.

Devices were fabricated in three pairs with bottom PTCDI-C13 layers fixed at lengths of 200 µm (devices #1 and #2, Figure S19), 400 µm (devices #3 and #4, Figure S20), and 600 µm (devices #5 and #6, Figure S21), respectively. Each pair consisted of one device with overlap (symmetric structure) and one without overlap (asymmetric structure) of the top PTCDI-C13 layer. Each device was biased in *Region II*, and power-spectral densities and transfer curves (*I*_D_-*V*_GS_, *V*_DS_ = –40 V) were recorded under identical conditions. The results are presented in Figures S19-21. In all three pairs, neither the peak current nor the *S*_ID_/*I*_D_^2^ spectrum showed systematic changes as the overlap was varied. Specifically, Figures S19-21 demonstrate that reducing or eliminating lateral overlap between top and bottom PTCDI-C13 layers does not meaningfully alter the electrical characteristics or the LFN amplitude.

These observations demonstrate that the excess LFN is not set by the absolute lateral area of the N-P-N overlap. Once the heterojunction is wide enough for electrons and holes to meet at the same defect sites, further widening simply replicates similar microscopic environments; local fluctuations average out, leaving the macroscopic spectrum constant. This insensitivity aligns well with the correlation model proposed in the main text: the large noise emerges only when trapping/detrapping and generation/recombination occur simultaneously at dual junctions, a condition governed primarily by the existence of overlapping interfaces rather than by their precise dimensions.

Finally, we emphasize that all six dual-junction devices consistently exhibit the same high-order spectral roll-off previously identified as characteristic of the BHN-NTC architecture, confirming that the enhanced stochastic behavior is intrinsic to the device structure and robust against moderate variations in overlap geometry.

**Note S7:** Device-to-device noise variation.

We measured five BHN-NTC transistors taken at random from the same fabrication run. Figure S22 plots the normalized noise spectra (*S*_ID_/*I*_D_^2^, *Region II*) for the set in the upper panel and the 500 Hz transient-current histograms in the lower panel. The spectra overlap almost completely, and the fitted standard deviations of the time-domain data fall in a narrow 0.118–0.127 range. These results confirm that normal alignment variations have no appreciable effect on either the frequency- or time-domain noise once the N-P-N junction is present.

**Reference**

[S1] K. Kobashi, R. Hayakawa, T. Chikyow, Y. Wakayama, *Nano Lett.* **2018**, 18, 4355.

[S2] H. Yoo, S. On, S. B. Lee, K. Cho, J. J. Kim, *Adv. Mater.* **2019**, 31, 1808265.

[S3] D. Panigrahi, R. Hayakawa, Y. Wakayama, *J. Mater. Chem. C* **2022**, 10, 5559.

[S4] C. Lee, C. Lee, S. Lee, J. Choi, H. Yoo, S. G. Im, *Nat.Commun.* **2023**, 14, 3757.

[S5] J. C. Shin, J. H. Lee, M. Jin, H. Lee, J. Kim, J. Lee, C. Lee, W. You, H. Yang, Y. S.

Kim, *ACS Nano* **2024**, 18, 1543.

[S6] H. Y. Lee, Y.-J. Oh, E. Joo, S. Jeong, J. Pyo, S. Cha, S. Pak, B. Kim, *ACS Appl. Mater.*

*Interfaces* **2025**.

[S7] H. Jiang, D. Belkin, S. E. Savel’ev, S. Lin, Z. Wang, Y. Li, S. Joshi, R. Midya, C. Li,

M. Rao, *Nat.Commun.* **2017**, 8, 882.

[S8] S. Pazos, W. Zheng, T. Zanotti, F. Aguirre, T. Becker, Y. Shen, K. Zhu, Y. Yuan, G.

Wirth, F. M. Puglisi, *Nanoscale* **2023**, 15, 2171.

[S9] S.-I. Kim, H.-J. You, M.-S. Kim, U.-S. An, M.-S. Kim, D. H. Lee, S.-T. Ryu, Y.-K.

Choi, *Sci. Adv.* **2024**, 10, eadk6042.

[S10] J. E. Seo, M. Gyeon, J. Seok, S. Youn, T. Das, S. Kwon, T. S. Kim, D. K. Lee, J. Y.

Kwak, K. Kang, *Adv. Funct. Mater* **2024**, 34, 2407382.

[S11] L. Jing, P. Yang, M. G. Moloney, Z. Zhang, Y. Wang, J. Li, F. Ma, J. Li, *Appl. Surf.*

*Sci.***2022**, 581, 152394.

[S12] J. Avossa, A. Bifulco, E. Amendola, F. Gesuele, S. L. Oscurato, Y. Gizaw, G.

Mensitieri, F. Branda, *Appl. Surf. Sci.* **2019**, 465, 73.

[S13] H. H. Kim, S. K. Lee, S. G. Lee, E. Lee, K. Cho, *Adv. Funct. Mater* **2016**, 26, 2070.
